# Visible Light-Promoted β-Functionalization of Carbonyl Compounds in the Presence of Organic Dyes

**DOI:** 10.1021/acs.joc.3c00890

**Published:** 2023-10-04

**Authors:** Luigi Dolcini, Tommaso Gandini, Riccardo Castiglioni, Alberto Bossi, Marta Penconi, Alberto Dal Corso, Cesare Gennari, Luca Pignataro

**Affiliations:** † Dipartimento di Chimica, Università degli Studi di Milano, via C. Golgi 19, Milano 20133, Italy; ‡ Istituto di Scienze e Tecnologie Chimiche “Giulio Natta” (SCITEC) del Consiglio Nazionale delle Ricerche (CNR), via Fantoli 16/15; SmartMatLab Center, via C. Golgi 19, Milano 20138, Italy

## Abstract

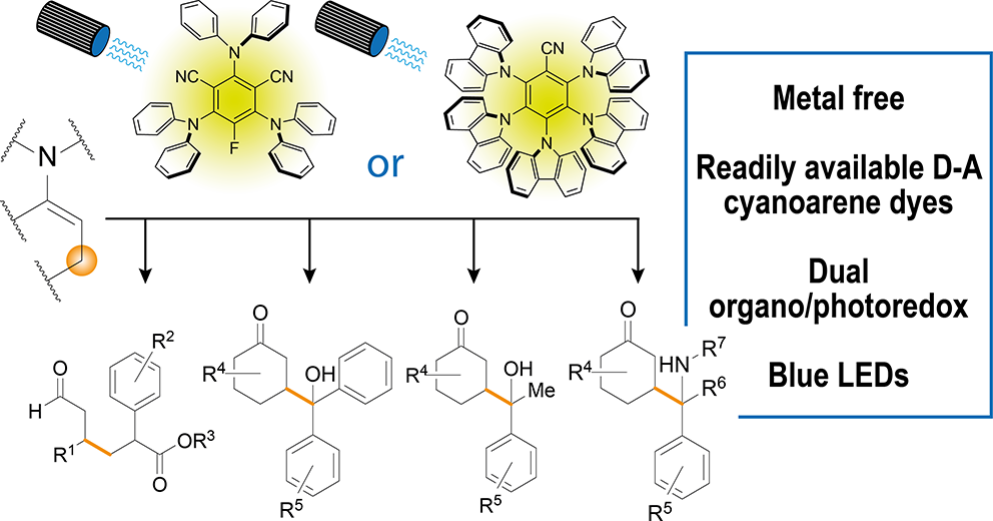

Herein, we investigate the use of organic photocatalysts in the visible light-promoted β-functionalization of carbonyl compounds. In particular, we studied the addition of aliphatic aldehydes to α,β-unsaturated compounds (β-Michael addition), and the reaction of cyclic ketones with either ketones (β-aldol condensation) or imines (β-Mannich reaction). Among the dyes tested, donor–acceptor cyanoarenes gave the best results, promoting the transformations of interest in moderate to good yields. The reaction scope was investigated on substrates with different steric and electronic properties. Fluorescence quenching analysis (Stern–Volmer experiments) led us to propose for these reactions a reductive quenching mechanism involving a transient 5πe^–^ activation mode.

## Introduction

Visible light photoredox catalysis^1^ has rapidly become a hot topic in organic synthesis because it provides access to reaction manifolds involving single-electron transfer events under mild conditions with a simple experimental setup.^2^ The controlled formation of radical intermediates discloses a range of transformations that cannot be realized with traditional ionic chemistry. Moreover, since the seminal paper by MacMillan on the enantioselective α-alkylation of aldehydes,^3^ it has been shown that the photocatalytic cycle can be effectively combined with other types of catalysis such as organo-^4^ or transition metal catalysis.^4a,5^ Photoredox catalysts are fluorescent or phosphorescent compounds absorbing light^6^ and displaying, among other properties (e.g., suitable redox potential values), an excited state lifetime (τ) sufficiently long to allow quenching by other molecules in solution. Given the crucial importance of τ, this research area has been dominated by phosphorescent Ru- and Ir-complexes, whose photoredox properties may be tuned with careful choice of ligand(s). However, the high cost and limited availability of precious metals soon stimulated the use of organic dyes as an alternative, which was pursued by several groups^1c,5d^ including ours.^7^ The main challenges associated with the use of organic dyes are (i) their excited state lifetime τ, which is quite short (<10 ns) in many cases, and (ii) the difficulty to modulate their photophysical properties. Indeed, many organic dyes are ionic molecules, difficult to purify, and offer limited room for systematic modification. A notable exception is represented by donor–acceptor (D–A) cyanoarenes, a class of compounds originally designed as OLED emitters^8^ and later employed in photocatalysis. These neutral molecules can be prepared in 1–2 steps from commercial products and feature a modular structure in which the number, type, and disposition of donor substituents may be easily varied.^9^

The present study aims at investigating the use of organic dyes in the β-functionalization of carbonyl compounds—complementary to the well-known α-functionalization—which clearly exemplifies the potential of dual organo/photoredox catalysis. This type of reactivity exploits a 5π-electron (5πe^–^) activation mode involving the formation of a transient β-enaminyl radical that reacts with a Michael acceptor (β-Michael^10^) or a persistent radical (β-arylation,^11^ β-aldol,^12^ and β-Mannich reaction^13^). This reactivity was discovered and developed by MacMillan and co-workers using Ir-photocatalysts^10–13^ and implemented by Krauss, Weix, and co-workers using CdSe semiconductor quantum dots.^14^ However, to the best of our knowledge, no examples have been reported using organic dye photocatalysts.

## Results and Discussion

**Scheme 1:**
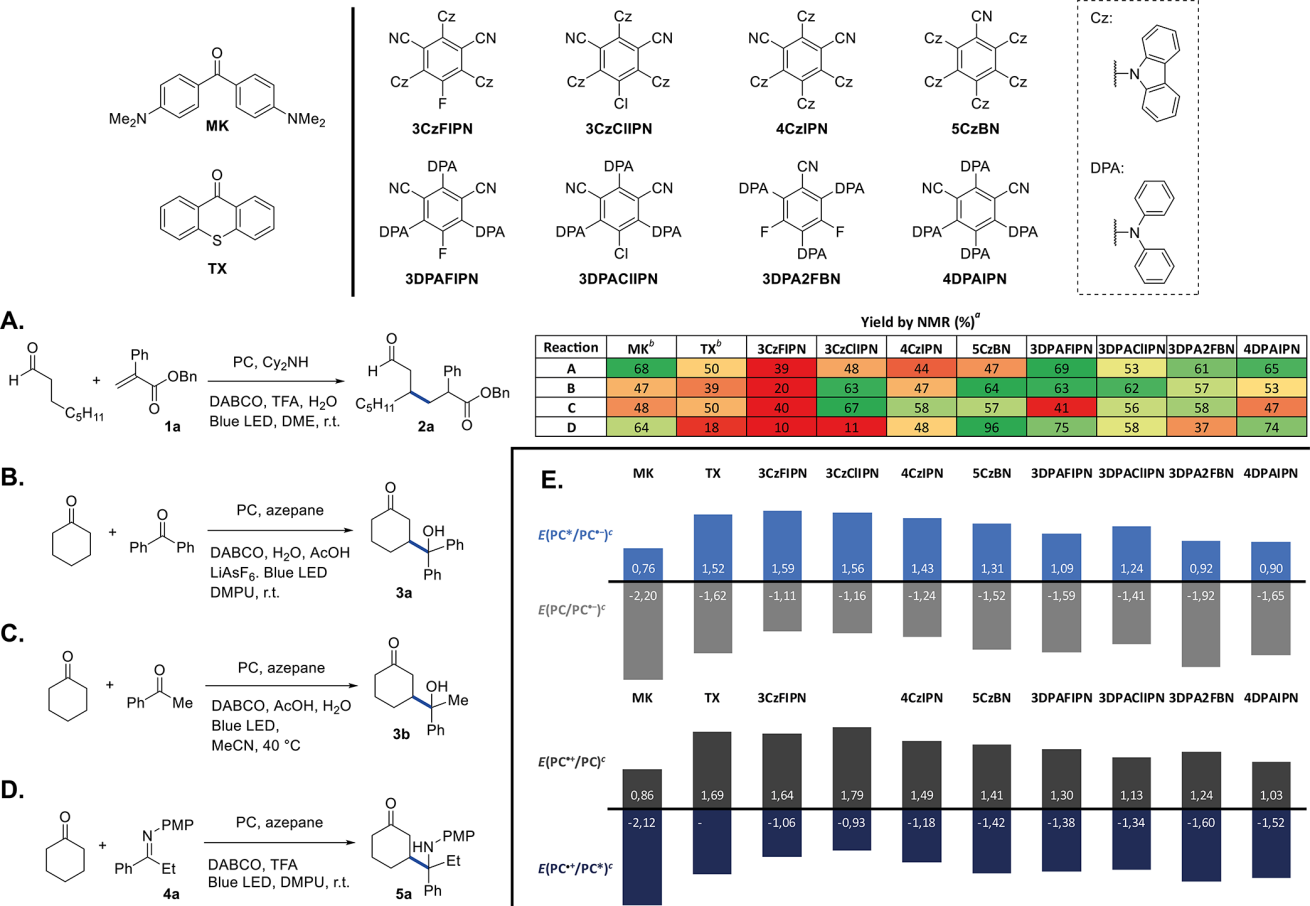
Screening of the Organic Dyes in the β-Michael (A), β-Aldol (B,C), and β-Mannich (D) Reactions, and Redox Potentials of the Photocatalysts (E) Irradiation performed at 427 nm with a 40 W LED lamp. Reaction time: 16 h. Reaction A: **1a** (1 equiv)/octanal (2 equiv)/PC (2 mol %)/Cy_2_NH (20 mol %)/DABCO (1 equiv)/TFA (20 mol %)/H_2_O (3 equiv)/DME (*C*_0,**1a**_ = 0.50 M); reaction B: benzophenone (1 equiv)/cyclohexanone (5 equiv)/PC (2 mol %)/azepane (20 mol %)/DABCO (2 equiv)/AcOH (20 mol %)/H_2_O (2 equiv)/LiAsF_6_ (1 equiv)/DMPU (*C*_0,benzophenone_ = 0.50 M); Reaction C: acetophenone (1 equiv)/cyclohexanone (10 equiv)/PC (2 mol %)/azepane (40 mol %)/AcOH (40 mol %)/DABCO (3 equiv)/H_2_O (2 equiv); MeCN (*C*_0,acetophenone_ = 0.17 M); Reaction D: **4a** (1 equiv)/cyclohexanone (5 equiv)/PC (2 mol %)/azepane (20 mol %)/TFA (20 mol %)/DABCO (1 equiv)/DMPU (*C*_0,**4a**_ = 0.75 M). Reaction yields determined by ^1^H NMR using 1,3,5-trimethoxybenzene as an internal standard. 390 nm LED light (40 W) employed. Potentials are expressed in V versus SCE.

We started our investigation by screening a series of organic dyes in the β-Michael of octanal with benzyl 2-phenyl acrylate **1a** (Scheme 1A) under conditions inspired by the protocol reported by MacMillan and co-workers.^10^ In these experiments, donor–acceptor cyanoarenes (D–A cyanoarenes) were found better than the Michler’s ketone (**MK**) and thioxanthone (**TX**), and clearly superior to the other dyes tested (see Table S1 in the Supporting Information for the full set of experiments). Examination of the redox potentials of the employed dyes (Scheme 1E) suggests that the most reducing photocatalysts [i.e., with strongly negative *E* (PC^•+^/PC*) and *E* (PC/PC^•–^) values] perform best. The most promising dyes were also screened in other reactions, such as the β-functionalization of cyclic ketones by reaction with ketones (β-aldol, Scheme 1B,C) and imines (β-Mannich, Scheme 1D), again using the reaction conditions reported by MacMillan.^12,13^ In these transformations, the D–A cyanoarenes generally performed better than **MK** and **TX**, although no clear correlation between redox potentials (Scheme 1E) and reaction yields could be established.

The photocatalysts performing best in terms of yield and selectivity were used for further investigations on each of the transformations studied: **3DPAFIPN** for the β-Michael, **3CzClIPN** for the β-aldol with alkyl-aryl ketones, **5CzBN** for the β-aldol with diaryl ketones and for the β-Mannich. For each model reaction shown in Scheme 1, we carried out a series of control experiments to assess the effect of the various experimental parameters. As expected, the β-Michael addition did not proceed in the absence of light, whereas some α-Michael addition took place (Table 1, entry 2). Varying the irradiation wavelength marginally affected the yield (see Table S2 in the Supporting Information). In the absence of photocatalyst, trace product formation was observed alongside some α-Michael adduct (entry 3), whereas reducing the amount of **3DPAFIPN** to 0.5 mol % left the yield unaffected (entry 4 vs 1). No desired product was observed when dicyclohexylamine was replaced by pyrrolidine (entry 5), thus confirming the crucial importance of using a bulky secondary amine.^10^ Still in agreement with MacMillan’s report,^10^ DABCO was found indispensable for conversion (entry 6). Notably, the reaction also proceeded with good yield when it was run under air (entry 7). For the β-aldol reaction, we ran the control experiments under two distinct sets of conditions optimized, respectively, for diaryl ketones (reaction I in Table 2) and for alkyl-aryl ketones (reaction II in Table 2).^12^ Indeed, the latter substrates are intrinsically more challenging, given the more difficult formation of the corresponding ketyl radicals [*E* (acetophenone/ketyl radical) = −2.14 V vs SCE; *E* (benzophenone/ketyl radical) = −1.83 V vs SCE].^12^ As expected, reaction II is less tolerant than the reaction I to modifications of the experimental conditions: while both transformations did not proceed in the absence of light (Table 2, entries 2 and 10), running the reaction with less or no photocatalyst had a stronger impact on reaction II (entries 3, 4 vs 11, 12),^15^ just as replacing azepane with pyrrolidine (entry 5 vs 13) or running the reaction under air (entry 8 vs 17). Working in the absence of DABCO led to a drastic drop in the yield in both reactions (entries 7 and 15). Finally, reaction II also benefited from the gentle heating produced by the LED lamp in the absence of a cooling fan (entry 9 vs 16).

**Table 1:**
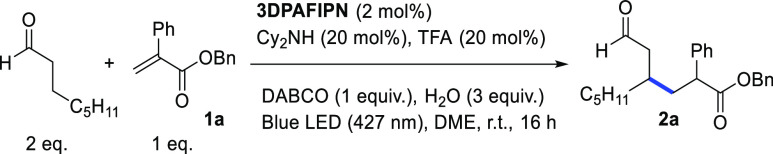
β-Michael Reaction of Aldehydes—Control Experiments^a^

#	deviation from the conditions above	yield (%)
1	none	69
2	no light	0^b^
3	no dye	8^b^
4	0.5 mol % dye	68
5	pyrrolidine instead of Cy_2_NH	0^b^
6	no DABCO	0
7	run under air	66

a Irradiation performed at 427 nm with a 40 W LED lamp. *C*_0,**1a**_ = 0.5 M. Before visible light irradiation, the reaction mixture was subjected to three freeze/pump/thaw cycles, backfilling with dry nitrogen. Yields were determined by ^1^H NMR using 1,3,5-trimethoxybenzene as internal standard.

b α-Michael product detected (10–30% yield).

**Table 2:**
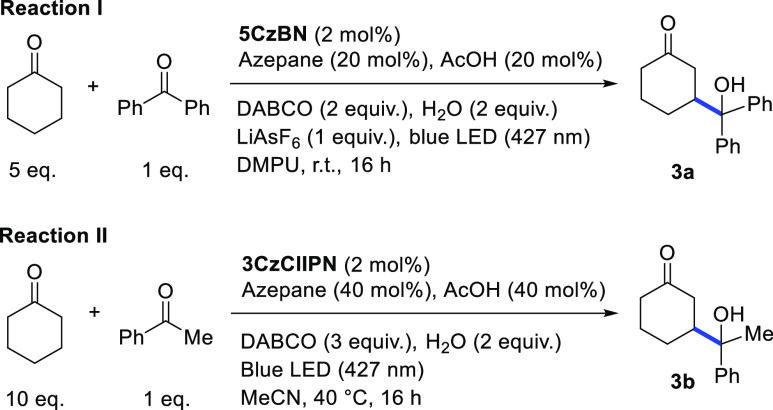
β-Aldol Reaction of Cyclic Ketones—Control Experiments^a^

#	reaction	deviation from the conditions above	yield (%)
1	I	none	64
2	I	no light	0
3	I	no dye	20
4	I	0.5 mol % dye	63
5	I	pyrrolidine instead of azepane	64
6	I	no AcOH	31
7	I	no DABCO	12
8	I	run under air	61
9	II	none	67
10	II	no light	0
11	II	no dye	0
12	II	0.5 mol % dye	32
13	II	pyrrolidine instead of azepane	<5
14	II	20 mol % azepane	48
15	II	no DABCO	14
16	II	run at r.t^b^	53
17	II	run under air	44

a Irradiation performed at 427 nm with a 40 W LED lamp. Reaction I: *C*_0,sub._ = 0.5 M; reaction II: *C*_0,sub._ = 0.17 M. Before visible light irradiation, the reaction mixture was subjected to three freeze/pump/thaw cycles, backfilling with dry nitrogen. Yields were determined by ^1^H NMR using 1,3,5-trimethoxybenzene as internal standard. Reaction I was irradiated in the presence of a cooling fan, whereas no fan was used for reaction II.

b Cooling fan used.

Control experiments on the β-functionalization of cyclic ketones with imines (β-Mannich reaction) showed that light, photocatalyst, and DABCO are all indispensable in order to observe any conversion (Table 3, entries 2, 3, and 7). Yet less dramatically, also the other modifications of the reaction conditions tested (lower dye loading, replacement of azepane with pyrrolidine, no TFA added, reaction run under air) had a negative effect on the yields (Table 3, entries 4–6 and 8).

**Table 3:**
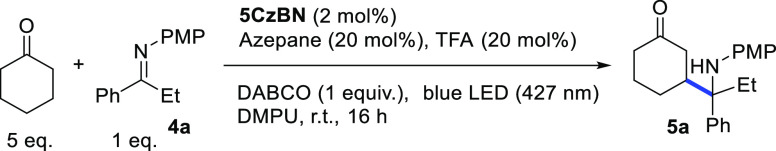
β-Mannich Reaction of Cyclic Ketones—Control Experiments^a^

#	deviation from the conditions above	yield (%)
1	none	96
2	no light	0
3	no dye	0
4	0.5 mol % dye	49
5	pyrrolidine instead of azepane	38
6	no TFA	32
7	no DABCO	0
8	run under air	66

a Irradiation performed at 427 nm with a 40 W LED lamp. *C*_0,**4a**_ = 0.75 M Before visible light irradiation, the reaction mixture was subjected to three freeze/pump/thaw cycles, backfilling with dry nitrogen. Yields were determined by ^1^H NMR using 1,3,5-trimethoxybenzene as internal standard.

**Scheme 2:**
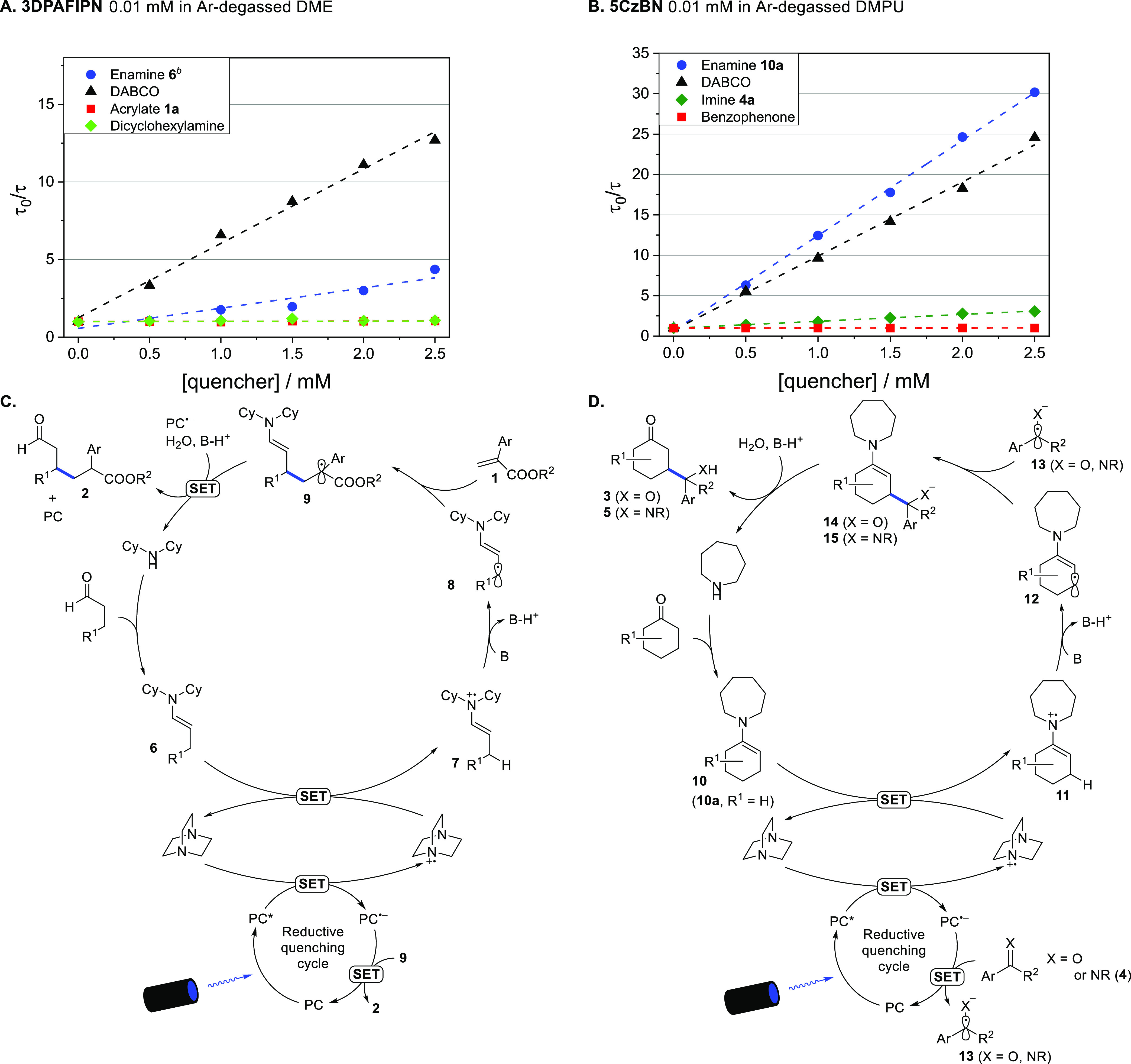
Stern–Volmer of Delayed Lifetimes with Photocatalysts 3DPAFIPN (A) and 5CzBN (B). Proposed Catalytic Cycle of β-Michael (C) and β-Aldol/β-Mannich Reactions (D) Excitation performed at 375 nm, emission detected, respectively, at 532 nm (A) and at 520 nm (B). Enamine formed in situ (assuming complete conversion) from a 15:1 mixture of octanal and dicyclohexylamine.

We ran a series of Stern–Volmer experiments to identify the main PC* quenching pathway in the transformations considered above and propose a mechanistic interpretation. A first set of experiments with **3DPAFIPN** in DME (Scheme 2A) showed that DABCO is the strongest quencher among the β-Michael reaction components. This is consistent with a PC* reductive quenching cycle in which DABCO mediates the oxidation of the electron-rich enamine (see Scheme 2C) to the corresponding radical cation (**7**),^16^ which readily undergoes β-deprotonation generating a 5πe^–^-activated intermediate (**8**). We propose that, consistent with MacMillan’s report,^10^ radical **8** is intercepted by the Michael acceptor,^17^ leading to a 3πe^–^ α-acyl radical intermediate (**9**). The latter is finally reduced by PC^•–^ to the corresponding enolate, whose protonation affords the β-Michael product (**2**). The yield trend observed (see Scheme 1E) suggests that the reduction of **9** [*E* (radical/enolate) = −0.59 to −0.73 V vs SCE]^10^ is a critical step, requiring strongly reducing dyes.

A second series of experiments was run using 5CzBN in the presence of the components of the β-aldol condensation with diaryl ketones and the β-Mannich reaction in DMPU (Scheme 2B). It was found that the competent enamine [1-(cyclohex-1-en-1-yl)azepane **10a**] is the strongest quencher of PC*, and DABCO is a close second. Considering that, in the catalytic reaction environment of the reactions considered, the concentration of DABCO (*C*_DABCO_) is 5–10 times higher than the maximum possible *C*_**10a**_ value, the dye is more favorably quenched by DABCO (see Table S5 in the Supporting Information) and thus we consider a DABCO-mediated enamine oxidation more likely than the direct reduction to radical cation **11** (Scheme 2D).^18^ After β-deprotonation, intermediate **11** evolves into the 5πe^–^-activated radical **12**, which is then trapped by the ketone- or imine-derived radical anion (**13**)—generated by the reaction of ketone or imine with PC^•–^ exploiting proton-coupled electron transfer^19^—to afford the reaction product (**3** or **4**). A similar scenario also emerged from the Stern–Volmer experiment run with 5CzBN—one of the best performing dyes in the β-aldol condensation with alkyl-aryl ketones^20^—in acetonitrile (see Figure S3 and Scheme S1 in the Supporting Information). Notably, the *k*_q_ values of PC’s prompt fluorescence quenching by DABCO (see Figure S4 and Table S7 in the Supporting Information) were found to be substantially similar to those of delayed fluorescence, which is in keeping with the fact that reaction yields are only moderately affected by the presence of air (see above).

**Scheme 3:**
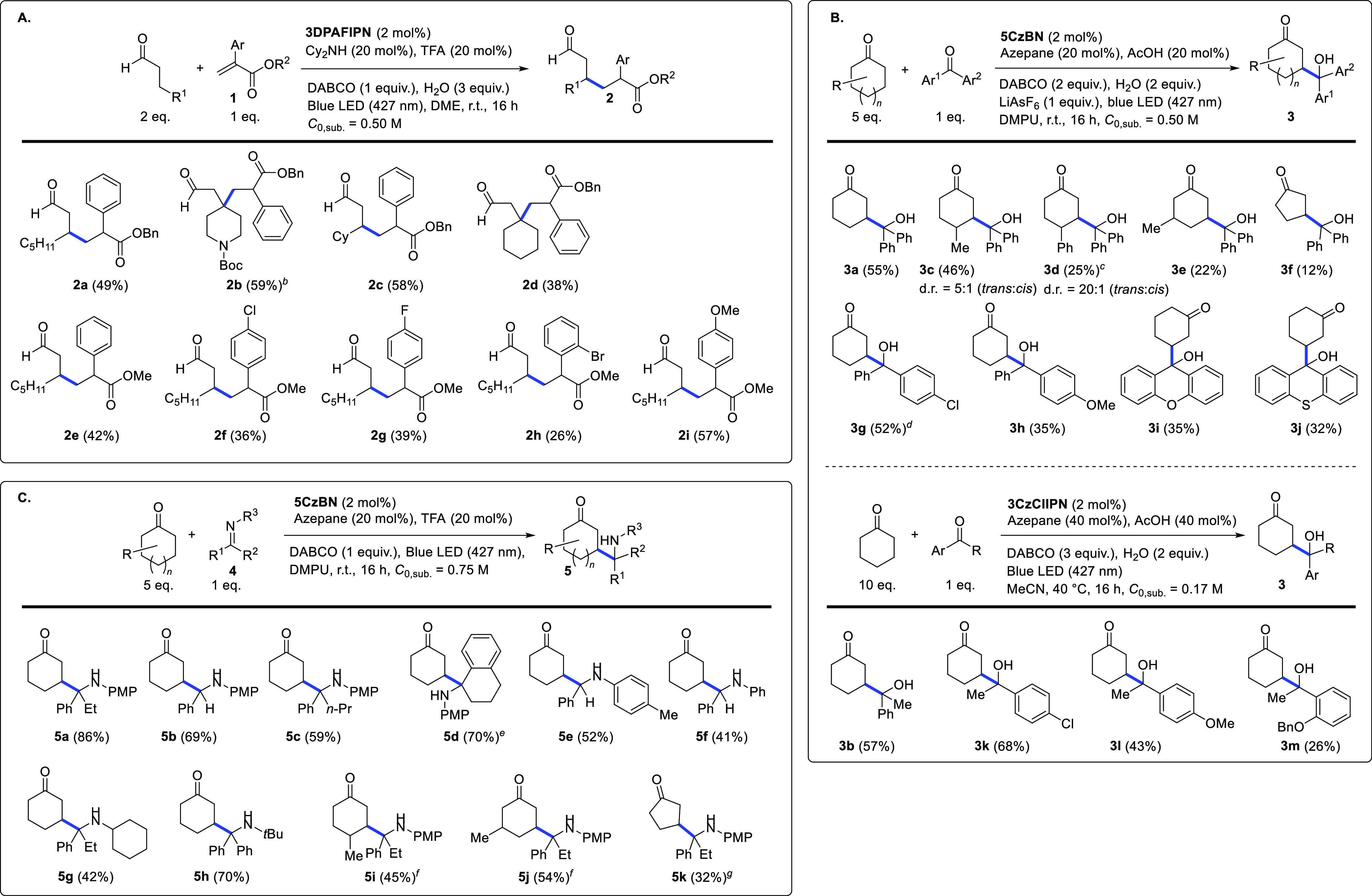
Substrate Scope Investigation in the β-Michael (A), β-Aldol (B), and β-Mannich (C) Reactions Irradiation performed at 427 nm with a 40 W LED lamp. Before visible light irradiation, the reaction mixture was subjected to three freeze/pump/thaw cycles, backfilling with dry nitrogen. The reactions were irradiated in the presence of a cooling fan. Isolated yields are displayed. For products with two (or three) stereocenters, diastereoisomeric ratio ∼1:1(:1:1), unless otherwise stated. AcOH was used in place of TFA. Reaction carried out without cooling fan. **3DPA2FBN** used as photocatalyst. **3DPAFIPN** used as photocatalyst. 10 equiv cyclic ketone, 40 mol % azepane, and 1 equiv LiBF_4_ were used. 10 equiv cyclic ketone, 40 mol % morpholine, and 1 equiv LiBF_4_ were used.

With the best reaction conditions available for each type of β-functionalization, we investigated the reaction scope obtaining the results shown in Scheme 3. For the β-Michael reaction (Scheme 3A), several aldehyde substrates were first screened with benzyl 2-phenyl acrylate (**1a**), affording the corresponding products **2a**–**d** in fair to good isolated yields (38–59%), irrespective of the steric bulk around the β-carbon. The β-alkylated aldehydes possessing two stereocenters were obtained as inseparable diastereoisomeric mixtures (∼1:1 ratio). The reaction of octanal with 2-phenyl acrylates differently substituted at the aromatic ring showed a trend of increasing yield with more electron-rich substrates, the 4-MeO-substituted product **2i** being obtained in better yield (57%) than electron-neutral **2e** (42%) and electron-deficient **2f**–**c** (36% and 39%, respectively). Unfortunately, acrylates devoid of a 2-aryl group failed to afford the desired product.

The β-aldol reaction was screened using several different cyclic ketones (Scheme 3B) that reacted with benzophenone in quite diverse yields the cyclohexanone- and 4-methylcyclohexanone-derived adducts **3a** and **3c** were obtained, respectively, in 58 and 46% yield, whereas other cyclic ketones were more sluggish to afford the corresponding products (**3d**–**e**). Cyclohexanone was successfully reacted with several diaryl ketones to afford products **3g**–**i** and, under the dedicated reaction conditions, with alkyl-aryl ketones: remarkably, the latter substrates were converted to the corresponding products (**3b**, **3j**–**k**) in yields generally higher than those of the former (affording compounds **3g**–**i**). Product **3k** was obtained in lower yield, seemingly due to the presence of the bulky –OBn substituent in the *ortho* position of its aromatic ring.

Finally, the scope of the β-Mannich reaction was investigated (Scheme 3C). To our delight, under the optimized conditions, cyclohexanone showed good reactivity with several imines, irrespective of the steric hindrance around the C=N bond. Products derived from both ketimines (**5a,c,d,g,h**) and aldimines (**5b,e,f**) were obtained in generally good isolated yields (up to 86%). Some different cyclic ketones were also screened in the reaction with propiophenone-derived PMP-imine **4a**, affording the corresponding products (**5i**–**j**) in moderate to good yields.

## Conclusions

In this contribution, we have shown that dual organo/photoredox catalysis for the β-functionalization of carbonyl compounds can be successfully carried out using readily available organic dyes (D–A cyanoarenes) instead of noble metal complexes. The feasibility of this approach was demonstrated in the β-Michael reaction of aldehydes and the β-aldol and β-Mannich reaction of cyclic ketones, which proceeded in moderate to good yields on several substrates. Fluorescence quenching experiments showed that these reactions involve a reductive quenching cycle producing a transient β-enaminyl radical, which is intercepted by a Michael acceptor (β-Michael) or by persistent radical (β-aldol and β-Mannich).

## Experimental Section

### General Section

The catalytic tests were performed in septum-sealed 10 mL microwave vials (borosilicate glass 3.3 according to ISO 3585). All reactions were performed with the Schlenk technique,^21^ under nitrogen or argon atmosphere unless otherwise specified. Irradiation was performed using Kessil PR160L lamps of the specified wavelength while cooling with a fan unless otherwise specified. Analytical thin-layer chromatography was carried out using commercial silica gel plates; spots were detected with UV light and revealed either with cerium-ammonium molybdate, ninhydrin, or 2,4-dinitrophenylhydrazine solution. Flash column chromatography was performed using silica gel (60 Å, particle size 40–64 μm) as stationary phase, following the procedure by Still and co-workers.^22^^1^H NMR spectra were recorded on a 400 MHz spectrometer. Proton chemical shifts are reported in ppm (δ) with the solvent reference relative to tetramethylsilane (TMS) employed as the internal standard (CDCl_3_, δ = 7.26 ppm).^23^ The following abbreviations are used: s = singlet, d = doublet, t = triplet, q = quartet, dd = doublet of doublets, dt = doublet of triplets, dq = doublet of quartets, tt = triplet of triplets, tq = quartet of triplets, and m = multiplet, broad signals are indexed br. (broad). ^13^C{^1^H} NMR spectra were recorded on a 400 MHz spectrometer operating at 100.56 MHz, with complete proton decoupling. Carbon chemical shifts are reported in ppm (δ) relative to TMS with the respective solvent resonance as the internal standard (CDCl_3_ δ = 77.16 ppm).^23^ Yield by NMR was determined by adding the internal standard (1,3,5-trimethoxybenzene) after the reaction time, before the workup, in a stock solution in ethyl acetate. All coupling constants are expressed in Hertz (Hz). IR spectra were recorded using a Jasco FT/IR-4600 ATR spectrometer. Mass spectrometry analyses were performed at the Mass Spectrometry facility of the Unitech COSPECT at the University of Milan (Italy).

### Materials

Dry solvents were either purchased from Acros Organics and Sigma-Aldrich (1,2-dimethoxyethane, *N*,*N*-dimethylpropylene urea) or distilled under nitrogen from calcium hydride (acetonitrile) or sodium/benzophenone (THF). Chemicals were purchased from Sigma-Aldrich, Fluorochem, and TCI or synthesized with reported and adapted literature procedures. Deuterated solvents were purchased from Deutero GmbH, EurisoTop, Sigma-Aldrich, or VWR. Photocatalysts **4CzIPN**,^9a^**3CzFIPN**,^24^**3CzClIPN**,^9b^**3DPAFIPN**,^9b^**3DPAClIPN**,^9b^**4DPAIPN**,^25^**5CzBN**,^9b^ and **3DPA2FBN**(9b) were synthesized following the procedures reported in the literature. 2-Substituted acrylates,^26^ noncommercial aldehydes,^27^ 2′-benzyloxyacetophenone,^26a^ and the imines^28^ were synthesized according to previously reported procedures.

### General Procedure 1 (β-Michael of Aldehydes)

To a vial containing a stirring bar, DABCO (28.0 mg, 0.25 mmol, 1 equiv) and **3DPAFIPN** (3.2 mg, 0.005 mmol, 2 mol %) were added. The vial was sealed and put under N_2_. A 0.5 M solution of acrylate **1** (0.25 mmol, 1 equiv, 0.50 mL) in DME was added, followed by dicyclohexylamine (10 μL, 0.05 mmol, 20 mol %), water (14 μL, 0.75 mmol, 3 equiv), trifluoroacetic acid (5 μL, 0.05 mmol, 20 mol %), and aldehyde (0.50 mmol, 2 equiv). The vial was then cooled into a liquid nitrogen bath and degassed via vacuum evacuation. The reaction vial was backfilled with N_2_ and warmed to room temperature. This purge-backfill procedure was repeated three times. The reaction vial was then placed at ca. 2 cm from a 427 nm Kessil lamp and stirred under a nitrogen atmosphere at room temperature (a fan was used to dissipate the heat generated by the lamp). After 16 h, the reaction was quenched with ethyl acetate and concentrated in vacuo. The crude was purified by column chromatography on silica gel (eluent: 95:5 to 8:2 hexane/diethyl ether) to afford the pure product.

### General Procedure 2 (β-Aldol Reaction of Cyclic Ketones and Aryl–Aryl Ketones)

To a vial containing a stirring bar **5CzBN** (7.0 mg, 7.5 μmol, 2 mol %), DABCO (84.1 mg, 0.75 mmol, 2 equiv), LiAsF_6_ (73.4 mg, 0.375 mmol, 1 equiv) and the aryl–aryl ketone (0.375 mmol, 1 equiv) were added. The vial was sealed and put under N_2_. Azepane (9 μL, 0.075 mmol, 20 mol %), cyclic ketone (1.85 mmol, 5 equiv), acetic acid (5 μL, 0.075 mmol, 0.2 equiv), water (14 μL, 0.75 mmol, 2 equiv), and DMPU (0.75 mL, *C*_0,sub._ = 0.5 M) were added to the reaction vial. The vial was then cooled into a liquid nitrogen bath and degassed via vacuum evacuation. The reaction vial was backfilled with N_2_ and warmed to room temperature. This purge-and-backfill procedure was repeated three times. The reaction vial was then placed ca. 2 cm from a 427 nm Kessil lamp and stirred under a nitrogen atmosphere at room temperature (a fan was used to dissipate the heat generated by the lamp). After 16 h, the reaction mixture was diluted with EtOAc and then washed with brine, water, and brine. The combined aqueous washings were extracted three times with EtOAc. The combined organic extracts were dried over Na_2_SO_4_, filtered, and concentrated in vacuo. The crude was purified by column chromatography on silica gel (eluent: 9:1 to 6:4 hexane/ethyl acetate) to afford the pure product.

### General Procedure 3 (β-Aldol Reaction of Cyclic Ketones and Alkyl–Aryl Ketones)

To a vial containing a stirring bar were added **3CzClIPN** (4.9 mg, 7.5 μmol, 2 mol %) and DABCO (126.2 mg, 1.13 mmol, 3 equiv) were added. The vial was sealed and put under N_2_. The alkyl–aryl ketone (0.375 mmol, 1 equiv), azepane (17 μL, 0.15 mmol, 40 mol %), the cyclic ketone (3.70 mmol, 10 equiv), acetic acid (9 μL, 0.15 mmol, 0.4 equiv), water (14 μL, 0.75 mmol, 2 equiv), and MeCN (2.2 mL, *C*_0,sub._ = 0.17 M) were added to the reaction vial. The vial was then cooled into a liquid nitrogen bath and degassed via vacuum evacuation. The reaction vial was backfilled with N_2_ and warmed to room temperature. This purge-and-backfill procedure was repeated three times. The reaction vial was then placed about 2 cm from a 427 nm Kessil lamp and stirred under a nitrogen atmosphere, without employing a cooling fan. After 16 h, the reaction was quenched with ethyl acetate and concentrated in vacuo. The crude was purified by column chromatography on silica gel (eluent: 9:1 to 6:4 hexane/ethyl acetate) to afford the pure product.

### General Procedure 4 (β-Mannich Reaction of Cyclic Ketones)

To a vial containing a stirring bar were added **5CzBN** (9.4 mg, 10 μmol, 2 mol %) and DABCO (56.2 mg, 0.50 mmol, 1 equiv) were added. The vial was sealed and put under N_2_. A 0.75 M solution of imine **4** (0.50 mmol, 1 equiv, 0.67 mL) in DMPU, the cyclic ketone (2.50 mmol, 5 equiv), azepane (12 μL, 100 μmol, 20 mol %), and trifluoroacetic acid (8 μL, 200 μmol, 0.2 equiv) were added to the reaction vial. The vial was then cooled into a liquid nitrogen bath and degassed via vacuum evacuation. The reaction vial was backfilled with N_2_ and warmed to room temperature. This purge-and-backfill procedure was repeated three times. The reaction vial was then placed about 2 cm from the 427 nm Kessil lamp and stirred under a nitrogen atmosphere at room temperature (a fan was used to dissipate the heat generated by the lamp). After 16 h, the reaction mixture was diluted with EtOAc and then washed with brine, water, and brine. The combined aqueous washings were extracted three times with EtOAc. The combined organic extracts were dried over Na_2_SO_4_, filtered, and concentrated in vacuo. The crude product was purified by column chromatography on silica gel (eluent: 95:5 to 8:2 hexane/ethyl acetate) to afford the pure product.

Characterization of new β-functionalization products is shown below (see Supporting Information for the NMR spectra of known β-functionalization products).

#### 3-(Hydroxydiphenylmethyl)-4-phenylcyclohexan-1-one (**3d**)

General procedure 2 was followed using DABCO (84.1 mg, 0.75 mmol, 2 equiv), **5CzBN** (7.0 mg, 0.0075 mmol, 2 mol %), LiAsF_6_ (73.4 mg, 0.375 mmol, 1 equiv), benzophenone (68.3 mg, 0.375 mmol, 1 equiv), azepane (9 μL, 0.075 mmol, 20 mol %), water (14 μL, 0.75 mmol, 2 equiv), acetic acid (5 μL, 0.075 mmol, 20 mol %), and 4-phenylcyclohexanone (322.3 mg, 1.85 mmol, 5 equiv) in DMPU (0.75 mL) for 16 h, without employing a cooling fan. Purification by flash chromatography (9:1 hexane/ethyl acetate) afforded compound **3d** as a pale yellow powder (20:1 mixture of trans/cis diastereoisomers,^29^ 3:1 mixture with the corresponding hemiacetal for the *trans* isomer; yield: 33.0 mg; 25%). mp 62–67 °C; ^1^H NMR (400 MHz, CDCl_3_): δ 7.74–7.61 (m, 3H), 7.43–7.00 (m, 11.3H), 6.89–6.71 (m, 0.7H), 3.65–3.55 (m, 0.25H), 3.44–3.36 (m, 0.75H), 3.21–3.13 (m, 0.75H), 3.05–2.96 (m, 0.25H, hemiacetal peak), 2.88–2.75 (m, 0.75H), 2.68 (dd, *J* = 15.4, 7.4 Hz, 0.25H), 2.57 (dd, *J* = 15.4, 4.2 Hz, 0.25H), 2.51–2.37 (m, 0.75H), 2.17–1.67 (m, 5H); ^13^C{^1^H} NMR (101 MHz, CDCl_3_): δ 214.3, 148.6, 147.4 (hemiacetal peak), 145.6 (hemiacetal peak), 145.5, 145.1 (hemiacetal peak), 144.0, 128.8, 128.6, 128.5, 128.3, 128.0, 127.7, 127.3, 127.2, 126.7, 126.6, 126.5, 126.1, 126.0, 125.7, 125.0, 107.0, 89.2 (hemiacetal peak), 82.2 (hemiacetal peak), 52.2, 47.9 (hemiacetal peak), 40.8 (hemiacetal peak), 40.6 (hemiacetal peak), 38.4, 38.0 (hemiacetal peak), 36.9, 36.5, 30.8 (hemiacetal peak), 22.7; IR (ATR): ν 3384, 3087, 3060, 3028, 2957, 2931, 2242, 1709, 1601, 1491, 1447, 1153, 990, 694 cm^–1^; HRMS (ESI): *m*/*z* calcd for [C_25_H_24_NaO_2_]^+^: 379.1674 [M + Na]^+^; found, 379.1674.

#### 3-(9-Hydroxy-9H-thioxanthen-9-yl)cyclohexan-1-one (**3j**)

General procedure 2 was followed using DABCO (84.1 mg, 0.75 mmol, 2 equiv), **5CzBN** (7.0 mg, 0.0075 mmol, 2 mol %), LiAsF_6_ (73.4 mg, 0.375 mmol, 1 equiv), thioxanthone (79.6 mg, 0.375 mmol, 1 equiv), azepane (9 μL, 0.075 mmol, 20 mol %), water (14 μL, 0.75 mmol, 2 equiv), acetic acid (5 μL, 0.075 mmol, 20 mol %), and cyclohexanone (195 μL, 1.85 mmol, 5 equiv) in DMPU (0.75 mL) for 16 h. Purification by flash chromatography (8:2 hexane/ethyl acetate) afforded compound **3j** as a yellow powder (yield: 36.7 mg; 32%). mp 177–185 °C; ^1^H NMR (400 MHz, CDCl_3_): δ 7.79 (dd, *J* = 7.8, 1.5 Hz, 1H), 7.70 (dd, *J* = 7.8, 1.5 Hz, 1H), 7.46–7.39 (m, 2H), 7.36–7.21 (m, 4H), 2.52–2.43 (m, 1H), 2.39 (m, 1H), 2.30 (br s, 1H), 2.27–2.12 (m, 2H), 2.00–1.91 (m, 1H), 1.90–1.84 (m, 1H), 1.65–1.54 (m, 1H), 1.40–1.28 (m, 2H); ^13^C{^1^H} NMR (101 MHz, CDCl_3_): δ 212.7, 139.2, 138.7, 130.7, 130.5, 127.5, 127.4, 127.0, 126.4, 126.3, 126.2, 126.1, 77.0, 42.3, 41.2, 40.9, 25.2, 24.6; IR (ATR): ν 3384, 2939, 2865, 1688, 1609, 1510, 1246, 1026 cm^–1^; HRMS (ESI): *m*/*z* calcd for [C_19_H_18_NaO_2_S]^+^: 333.0925 [M + Na]^+^; found, 333.0928.

#### 3-(1-Hydroxy-1-(4-methoxyphenyl)ethyl)cyclohexan-1-one (**3l**)

General procedure 3 was followed using DABCO (126.2 mg, 1.125 mmol, 3 equiv), **3CzClIPN** (7.0 mg, 0.0075 mmol, 2 mol %), azepane (17 μL, 0.15 mmol, 40 mol %), water (14 μL, 0.75 mmol, 2 equiv), acetic acid (9 μL, 0.15 mmol, 40 mol %), 4′-methoxyacetophenone (56.3 mg, 0.375 mmol, 1 equiv), and cyclohexanone (390 μL, 3.70 mmol, 10 equiv) in MeCN (2.2 mL) for 16 h. Purification by flash chromatography (93:7 dichloromethane/ethyl acetate) afforded compound **3l** as a pale brown powder (yield: 40.0 mg; 43%). mp 82–85 °C; ^1^H NMR (400 MHz, CDCl_3_): δ 7.34–7.27 (m, 2H), 6.93–6.84 (m, 2H), 3.81 (s, 1.5H, diast. 1), 3.80 (s, 1.5H, diast. 2), 2.53–2.45 (m, 0.5H), 2.36–2.28 (m, 1H), 2.26–1.97 (m, 5H), 1.72–1.65 (m, 0.5H), 1.58 (s, 1.5H, diast. 1), 1.54 (s, 1.5H, diast. 2), 1.52–1.33 (m, 2H); ^13^C{^1^H} NMR (101 MHz, CDCl_3_): δ 212.8, 158.4, 138.9, 138.6, 126.3, 113.5, 75.5, 75.4, 55.3, 49.7, 49.7, 43.1, 43.0, 41.2, 41.2, 27.5, 27.4, 25.8, 25.5, 25.0; IR (ATR): ν 3384, 2939, 2865, 2835, 1688, 1609, 1510, 1246, 1026 cm^–1^; HRMS (ESI): *m*/*z* calcd for [C_15_H_20_NaO_3_]^+^: 271.1310 [M + Na]^+^; found, 271.1310.

#### 3-(1-(2-(Benzyloxy)phenyl)-1-hydroxyethyl)cyclohexan-1-one (**3m**)

General procedure 3 was followed using DABCO (126.2 mg, 1.125 mmol, 3 equiv), **3CzClIPN** (7.0 mg, 0.0075 mmol, 2 mol %), azepane (17 μL, 0.15 mmol, 40 mol %), water (14 μL, 0.75 mmol, 2 equiv), acetic acid (9 μL, 0.15 mmol, 40 mol %), 1-(2-(benzyloxy)phenyl)ethan-1-one^30^ (84.8 mg, 0.375 mmol, 1 equiv) and cyclohexanone (390 μL, 3.70 mmol, 10 equiv) in MeCN (2.2 mL) for 16 h. Purification by flash chromatography (7:3 hexane/ethyl acetate) afforded compound **3m** as a yellowish oil (1:1 mixture of diastereoisomers; yield: 31.4 mg; 26%). ^1^H NMR (400 MHz, CDCl_3_): δ 7.45–7.33 (m, 5H), 7.32–7.28 (m, 1H), 7.25–7.19 (m, 1H), 7.02–6.94 (m, 2H), 5.12 (s, 2H), 3.99 (s, 0.5H, diast. 1), 3.91 (s, 0.5H, diast. 2), 2.51–2.40 (m, 1H), 2.40–2.27 (m, 2H), 2.26–2.22 (m, 0.5H), 2.21–2.15 (m, 1H), 2.08–1.99 (m, 1H), 1.93–1.85 (m, 0.5H), 1.78–1.67 (m, 1H), 1.59 (s, 1.5H, diast. 1), 1.54 (s, 1.5H, diast. 2), 1.50–1.41 (m, 2H); ^13^C{^1^H} NMR (101 MHz, CDCl_3_): δ 212.9, 212.6, 155.8, 155.8, 136.2, 134.1, 129.0, 128.5, 127.7, 127.6, 121.3, 121.3, 112.9, 77.0, 76.7, 70.8, 47.6, 43.9, 43.1, 41.5, 41.4, 26.3, 25.8, 25.4, 25.3, 24.7, 23.8; IR (ATR): ν 3482, 2939, 2864, 1670, 1446, 1222, 730 cm^–1^; HRMS (ESI): *m*/*z* calcd for [C_21_H_24_NaO_3_]^+^: 347.1623 [M + Na]^+^; found, 347.1624.

#### 3-(1-((4-Methoxyphenyl)amino)-1-phenylpropyl)-4-methylcyclohexan-1-one (**5i**)

General procedure 4 was followed using DABCO (56.1 mg, 0.50 mmol, 1 equiv), **5CzBN** (9.3 mg, 0.01 mmol, 2 mol %), LiBF_4_ (46.9 mg, 0.50 mmol, 1 equiv), azepane (24 μL, 0.20 mmol, 40 mol %), trifluoroacetic acid (8 μL, 0.10 mmol, 20 mol %), imine **4a**(28a) (119.7, 0.50 mmol, 1 equiv) and 4-methylcyclohexanone (610 μL, 5.0 mmol, 10 equiv) in DMPU (0.67 mL) for 16 h. Purification by flash chromatography (85:15 hexane/ethyl acetate) afforded two fractions containing, respectively, a mixture of 3 diastereoisomers of compound **5i** (diast. 1, 2, 3/fraction 1) and the fourth pure diastereoisomer (diast. 4/fraction 2). Total yield (4 diastereoisomers in a 1:1:1:1 ratio): 79.6 mg; 45%. mp (fraction 1) = mp (fraction 2) = 45–50 °C.

Fraction 1 (diast. 1, 2, 3 as 1:1:1 mixture, white foam): ^1^H NMR (400 MHz, CDCl_3_): δ 7.56–7.49 (m, 1.33H), 7.46–7.41 (m, 0.67H), 7.39–7.27 (m, 3H), 6.63–6.57 (m, 1.33H), 6.55–6.51 (m, 0.67H), 6.33–6.22 (m, 1.33H), 6.13–6.07 (m, 0.67H), 3.68 (s, 1H), 3.67 (s, 1H), 3.64 (s, 1H), 2.94–2.87 (m, 0.33H), 2.56–2.36 (m, 2H), 2.36–2.06 (m, 5.67H), 1.99–1.91 (m, 0.67H), 1.85–1.70 (m, 2H), 1.18 (d, *J* = 7.0 Hz, 1.33H), 1.15–0.95 (m, 1.33H), 0.82–0.71 (m, 2.67H), 0.68–0.63 (m, 1H); ^13^C{^1^H} NMR (101 MHz, CDCl_3_): δ 213.6, 212.3, 211.3, 151.8, 151.8, 151.7, 142.7, 142.6, 141.3, 139.9, 139.8, 139.4, 128.8, 128.2, 128.0, 128.0, 127.9, 127.1, 126.9, 126.8, 116.8, 116.4, 114.3, 64.8, 63.8, 62.6, 55.5, 49.7, 49.1, 48.2, 40.4, 39.8, 39.0, 36.4, 36.2, 34.7, 34.4, 29.7, 28.8, 28.5, 27.9, 27.0, 26.7, 24.8, 23.7, 23.6, 12.8, 12.1, 8.2, 7.5, 7.1; IR (ATR): ν 3379, 2939, 2865, 1688, 1609, 1509, 1245, 1026, 827 cm^–1^; HRMS (ESI): *m*/*z* calcd for [C_23_H_29_NNaO_2_]^+^: 374.2096 [M + Na]^+^; found, 374.2095.

Fraction 2 (pure diast. 4, white foam): ^1^H NMR (400 MHz, CDCl_3_): δ 7.54–7.48 (m, 2H), 7.38–7.26 (m, 3H), 6.61–6.55 (m, 2H), 6.25–6.19 (m, 2H), 3.67 (s, 3H), 3.53 (br s, 1H), 2.68–2.59 (m, 1H), 2.57–2.48 (m, 1H), 2.38–2.27 (m, 2H), 2.13–2.01 (m, 3H), 1.56–1.48 (m, 1H), 1.32–1.25 (m, 1H), 1.24–1.12 (m, 1H), 1.08 (d, *J* = 6.8 Hz, 3H), 0.84 (t, *J* = 7.2 Hz, 3H); ^13^C{^1^H} NMR (101 MHz, CDCl_3_): δ 214.8, 152.0, 141.8, 139.6, 128.3, 128.3, 127.2, 116.8, 114.4, 64.0, 55.6, 47.0, 39.1, 35.5, 27.9, 27.1, 24.3, 22.5, 7.9; IR (ATR): ν 3368, 2932, 1705, 1508, 1234, 1034 cm^–1^; HRMS (ESI): *m*/*z* calcd for [C_23_H_29_NNaO_2_]^+^: 374.2096 [M + Na]^+^; found, 374.2095.

The synthesis of products **2b** and **3k** was repeated on a larger scale, giving the following results.

#### *tert*-Butyl 4-(3-(Benzyloxy)-3-oxo-2-phenylpropyl)-4-(2-oxoethyl)piperidine-1-carboxylate (**2b**)^10^

General procedure 1 for the β-Michael reaction was followed using DABCO (112.2 mg, 1.00 mmol, 1 equiv), **3DPAFIPN** (13.0 mg, 0.02 mmol, 2 mol %), benzyl 2-phenyl acrylate^26a^ (238.3 mg, 1.00 mmol, 1 equiv), dicyclohexylamine (40 μL, 0.20 mmol, 20 mol %), water (54 μL, 3.00 mmol, 3 equiv), acetic acid (12 μL, 0.20 mmol, 20 mol %) and *N*-Boc-4-piperidineacetaldehyde (454.6 mg, 2.00 mmol, 2 equiv) in DME (2.0 mL) for 24 h, without employing a cooling fan. Purification by flash chromatography (85:15 hexane/acetone) afforded compound **2b** as a yellowish oil (yield: 224.0 mg; 48%). ^1^H NMR (400 MHz, CDCl_3_): δ 9.68 (t, *J* = 2.4 Hz, 1H), 7.43–7.15 (m, 10H), 5.15 (d, *J* = 12.3 Hz, 1H), 5.00 (d, *J* = 12.3 Hz, 1H), 3.72 (dd, *J* = 8.5, 4.4 Hz, 1H), 3.52–3.33 (m, 2H), 3.34–3.17 (m, 2H), 2.56 (dd, *J* = 14.8, 8.5 Hz, 1H), 2.38–2.33 (m, 2H), 1.92 (dd, *J* = 14.8, 4.4 Hz, 1H), 1.46–1.42 (m, 13H).

#### 3-(1-(4-Chlorophenyl)-1-hydroxyethyl)cyclohexan-1-one (**3k**)^12^

General procedure 3 was followed using DABCO (378.6 mg, 3.375 mmol, 3 equiv), **3CzClIPN** (14.8 mg, 0.0225 mmol, 2 mol %), azepane (51 μL, 0.45 mmol, 40 mol %), water (41 μL, 2.25 mmol, 2 equiv), acetic acid (26 μL, 0.45 mmol, 40 mol %), 4′-chloroacetophenone (146 μL, 1.125 mmol, 1 equiv) and cyclohexanone (1.20 mL, 11.25 mmol, 10 equiv) in MeCN (6.6 mL) for 24 h. Purification by flash chromatography (7:3 hexane/ethyl acetate) afforded compound **3k** as a colorless oil (1:1 mixture of diastereoisomers; yield: 130.5 mg; 46%). ^1^H NMR (400 MHz, CDCl_3_): δ 7.44–7.26 (m, 4H), 2.57–2.45 (m, 0.5H), 2.45–2.34 (m, 1H), 2.33–2.18 (m, 2H), 2.18–1.97 (m, 3.5H), 1.96–1.86 (m, 0.5H), 1.80–1.67 (m, 1H), 1.59 (s, 1.5H, diast. 1), 1.54 (s, 1.5H, diast. 2), 1.51–1.31 (m, 1.5H).

## Data Availability

The data underlying this study are available in the published article and in its Supporting Information.

## Supporting Information Available

General procedures, experimental details, characterization data, NMR spectra, fluorescence-quenching experiments (PDF)
