# Supplementary material for: Visible Light-Promoted β-Functionalization of Carbonyl Compounds in the Presence of Organic Dyes
Source: J Org Chem. 2023 Oct 4;88(20):14283–91. doi: 10.1021/acs.joc.3c00890 (PMC10594657; doi:10.1021/acs.joc.3c00890)
Supplement: Supplementary file 1 — jo3c00890_si_001.pdf [file jo3c00890_si_001.pdf]

Supporting Information

Visible Light-promoted β-Functionalization of Carbonyl Compounds in the Presence of Organic Dyes

Luigi Dolcini,[a] Tommaso Gandini,[a] Riccardo Castiglioni,[a] Alberto Bossi,[b] Marta Penconi,[b] Alberto Dal Corso,[a] Cesare Gennari,[a] Luca Pignataro[a]*

[a] Università degli Studi di Milano, Dipartimento di Chimica, via C. Golgi 19 - 20133 Milano (Italy)

[b] CNR-Institute of Chemical Sciences and Technologies (SCITEC) "Giulio Natta", via Fantoli 16/15 - 20138 Milano (Italy)

Correspondence to: luca.pignataro@unimi.it

Summary

General remarks S2

Materials S2

Catalytic tests in β-alkylation of aldehydes S3

Stern-Volmer analysis S4

Catalytic cycle proposed for the β-aldol reaction of cyclic ketones with aryl-alkyl ketones S7

Reaction setup S9

General Procedure for the reactions S10

- Synthesis of β-Michael products S11
- Synthesis of β-aldol products from aryl-aryl ketones S14
- Synthesis of β-aldol products with aryl-alkyl ketones S19
- Synthesis of β-Mannich products S21

NMR spectra of the isolated products S26

References S53

General remarks

The catalytic tests were performed in septum-sealed 10 mL microwave vials (borosilicate glass 3.3 acc. to ISO 3585). All reactions were performed with the Schlenk technique,[1] under nitrogen or argon atmosphere, unless otherwise specified. Irradiation was performed using Kessil PR160L lamps of the specified wavelength while cooling down with a fan (unless otherwise specified). Analytical thin layer chromatography (TLC) was carried out using commercial silica gel plates, spots were detected with UV light and revealed either with cerium-ammonium molybdate, ninhydrin or 2,4-dinitrophenylhydrazine solution. Flash column chromatography was performed using silica gel (60 Å, particle size 40-64 μm) as stationary phase, following the procedure by Still and co-workers.[2] ^{1}H NMR spectra were recorded on a 400 MHz spectrometer. Proton chemical shifts are reported in ppm (δ) with the solvent reference relative to tetramethylsilane (TMS) employed as the internal standard ($CDCl_3$, δ = 7.26 ppm).[3] The following abbreviations are used: s = singlet, d = doublet, t = triplet, q = quartet, dd = doublet of doublets, dt = doublet of triplets, dq = doublet of quartets, tt = triplet of triplets, tq = quartet of triplets and m = multiplet, broad signals are indexed br. (broad). $^{13}C\{^{1}H\}$ NMR spectra were recorded on a 400 MHz spectrometer operating at 100.56 MHz, with complete proton decoupling. Carbon chemical shifts are reported in ppm (δ) relative to TMS with the respective solvent resonance as the internal standard ($CDCl_3$ δ = 77.16 ppm).[3] Yield by NMR were determined by adding the internal standard (1,3,5-trimethoxybenzene) after the reaction time, before the work-up, in a stock solution in ethyl acetate. All coupling constants are expressed in Hertz (Hz). IR spectra were recorded using a Jasco FT/IR-4600 ATR spectrometer. Mass spectrometry analyses were performed at the Mass Spectrometry facility of the Unitech COSPECT at the University of Milan (Italy).

Materials

Dry solvents were either purchased from Acros Organics and Sigma-Aldrich (1,2-dimethoxyethane, *N*,*N*-dimethylpropylene urea), or distilled under nitrogen from calcium hydride (acetonitrile) or sodium/benzophenone (THF). Chemicals were purchased from Sigma Aldrich, Fluorochem and TCI, or synthesized with reported and adaprted literature procedures. Deuterated solvents were purchased from Deutero GmbH, EurisoTop, Sigma-Aldrich or VWR.

Photocatalysts **4CzIPN**,[4] **3CzFIPN**,[5] **3CzClIPN**,[6] **3DPAFIPN**,[6] **3DPAClIPN**,[6] **4DPAIPN**,[7] **5CzBN**[6] and **3DPA2FBN**[6] were synthesized following the procedures reported in the literature.

Catalytic tests in *β*-alkylation of aldehydes

Table S1. Dye screening.[a]

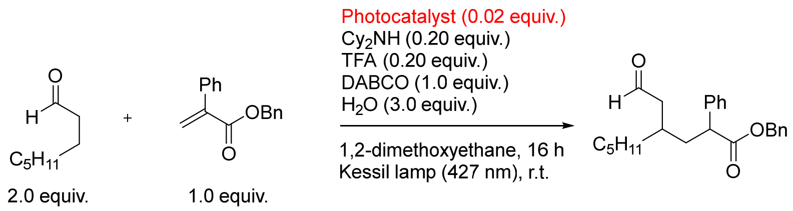

#	Photocatalyst	Yield (%)[b]
1	**3DPAFIPN**	69
2	Michler's ketone (**MK**)[c]	68
3	**4DPAIPN**	65
4	**3DPA2FBN**	61
5	**3DPAClIPN**	53
6	Thioxanthone (**TX**)[c]	50
7	**3CzClIPN**	48
8	**5CzBN**	47
9	**4CzIPN**	44
10	**3CzFIPN**	39
11	EosinY[d]	0
12	Rose Bengal[d]	0
13	Congo red[d]	0
15	Rhodamine 6G[d]	0
16	Rhodamine B[d]	0
17	Methyl orange[e]	0
18	[Mes-Acr-Me]$^+$	0

[a] Reaction conditions: Benzyl 2-phenylacrylate = 0.25 mmol; $C_{0,\mathrm{sub.}}$= 0.5 M; 1,2-dimethoxyethane; rt; 16 h; irradiation performed at 427 nm with a 40 W LED lamp. [b] Determined by ^{1}H NMR with the internal standard. [c] Irradiated using a different Kessil lamp (390 nm). [d] Irradiated using a different Kessil lamp (525 nm). [d] Irradiated using a different Kessil lamp (456 nm).

Table S2. Kessil lamp screening.[a]

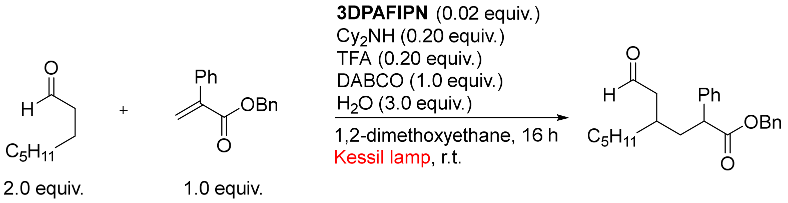

#	Kessil lamp (nm)	Yield (%)[b]
1	390	55
2	427	69
3	456	68
4	525	46

[a] Reaction conditions: Benzyl 2-phenylacrylate = 0.25 mmol; $C_{0,\text{sub.}}$= 0.5 M; 1,2-dimethoxyethane; rt; 16 h; irradiation performed using a 40 W lamp. [b] Determined by ^{1}H NMR with the internal standard.

Stern-Volmer analysis

Steady state emission spectra and photoluminescence lifetimes were obtained with a FLS 980 spectrofluorimeter (Edinburgh Instruments Ltd.). Continuous excitation for the steady state measurements was provided by a 450 W Xenon arc lamp. Emission spectra were corrected for the wavelength-dependent sensitivity of the detector. Photoluminescence time-resolved measurements were carried out by TCSPC (time-correlated single-photon counting) method with an Edinburgh Picosecond Pulsed Diode Laser EPL-375 (Edinburgh Instruments Ltd.) and fitted with a sum of exponential decay to obtain the lifetimes of prompt and delayed fluorescence. Photoluminescence experiments were carried out in argon-degassed solutions, unless otherwise specified.

A 0.01 mM stock solution of photocatalyst was used to prepare a 0.01 M solution of the quencher. Aliquots of this solution were added to a 5 mL volumetric flask and diluted with the same (0.01 mM) photocatalyst solution. All solutions were degassed with argon for 15 minutes before measuring time-resolved luminescence. The obtained lifetimes of the (delayed or prompt) fluorescence were plotted against the quencher concentration according to the Stern-Volmer equation:

$$\frac{\tau_0}{\tau} = 1 + k_q \tau_0 [Q]$$

where τ_0 and τ are the lifetimes of (delayed or prompt) fluorescence in the absence and in the presence of quencher Q, and k_q is the quenching rate constant, which was calculated from the slope of the linear fit against the quencher concentration $[Q]$. The Stern-Volmer quenching constant K_{SV} is obtained from the following relationship:

$$k_q \tau_0 = K_{SV}$$

Quenching efficiencies (η) for the study of quenching of β-aldol and β-Mannich reactions are calculated by the following equation:

$$\eta = 1 - \frac{\tau}{\tau_0}$$

Enamine **10a** was synthesized from cyclohexanone and azepane according to a literature procedure,[8] using a Dean-Stark apparatus to subtract water. The product was then purified it by high vacuum distillation (T_{bath} = 124 °C). Due to its sensitivity to moisture, the solutions employing enamine **10a** as quencher were prepared inside a glove box.

Figure S1. Stern-Volmer plot of delayed lifetimes (excitation at 375 nm, emission at 532 nm) of 0.01 mM **3DPAFIPN** solutions in DME in the presence of enamine **6** (formed *in situ*, assuming complete conversion, from a 15:1 mixture of octanal and dicyclohexylamine), DABCO, acrylate **1a** and dicyclohexylamine.

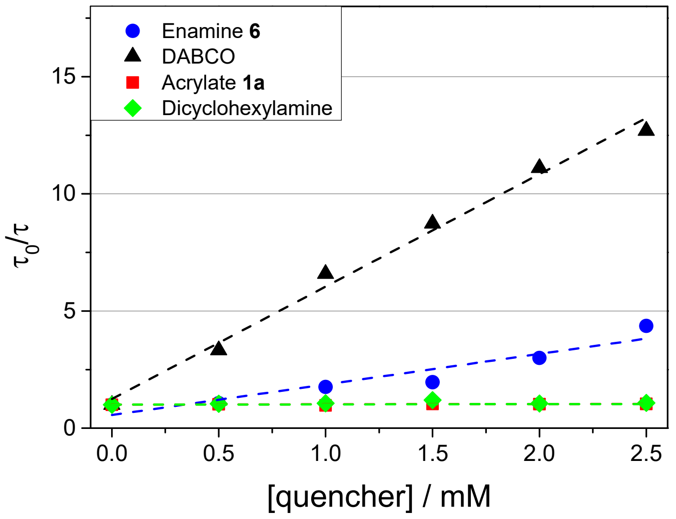

Table S3. Stern-Volmer constants (K_{SV}) and quenching rate constants (k_q) of **3DPAFIPN** in DME.

Quencher	K_{SV} (M^{-1})	k_q (s^{-1} M^{-1})
Enamine **6**	1304	$2.23 \cdot 10^8$
DABCO	4798	$7.71 \cdot 10^8$
Acrylate **1a**	11.8	$1.90 \cdot 10^6$
Dicyclohexylamine	12.9	$2.20 \cdot 10^6$

Figure S2. Stern-Volmer plot of delayed lifetimes (excitation at 375 nm, emission at 520 nm) of 0.01 mM **5CzBN** solutions in DMPU in the presence of enamine **10a**, DABCO, imine **4a** and benzophenone.

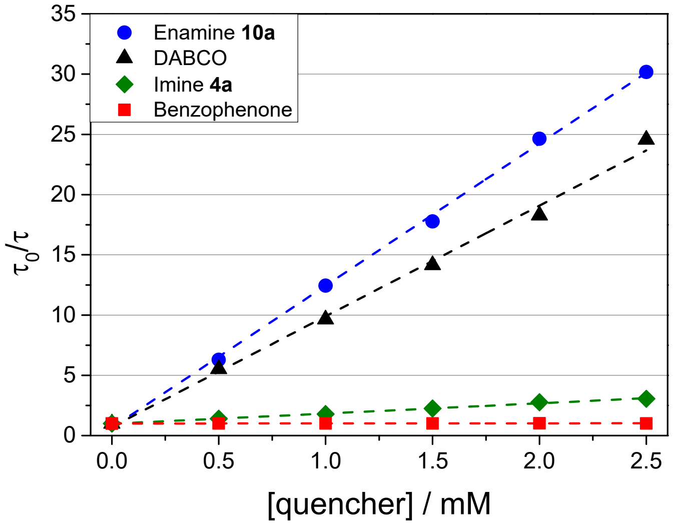

Table S4. Stern-Volmer constants (K_{SV}) and quenching rate constants (k_q) of **5CzBN** in DMPU.

Quencher	K_{SV} (M^{-1})	k_q (s^{-1} M^{-1})
Enamine **10a**	11785	$2.31 \cdot 10^9$
DABCO	9177	$1.80 \cdot 10^9$
Imine **4a**	849.5	$1.70 \cdot 10^8$
Benzophenone	9.96	$1.87 \cdot 10^6$

From the K_{SV} values obtained and by using the above-shown equations, we calculated the quenching efficiencies of enamine and DABCO at their estimated concentrations (C_0) in the reaction environment.

Table S5. Quenching efficiencies of the strongest quenchers under catalytic reaction conditions.

Reaction	Quencher	K_{SV} (M^{-1})	C_0 (M)	τ_0/τ	Quenching efficiency η (%)
β-aldol on diaryl ketones	Enamine **10a**	11785	0.0794	936	99.89
	DABCO	9177	0.794	7284	99.99
β-Mannich	Enamine **10a**	11785	0.108	1268	99.92
	DABCO	9177	0.538	4935	99.98

Figure S3. Stern-Volmer plot of delayed lifetimes (excitation at 375 nm, emission at 520 nm) of 0.01 mM **5CzBN** solutions in MeCN in the presence of enamine **10a**, DABCO, and acetophenone.

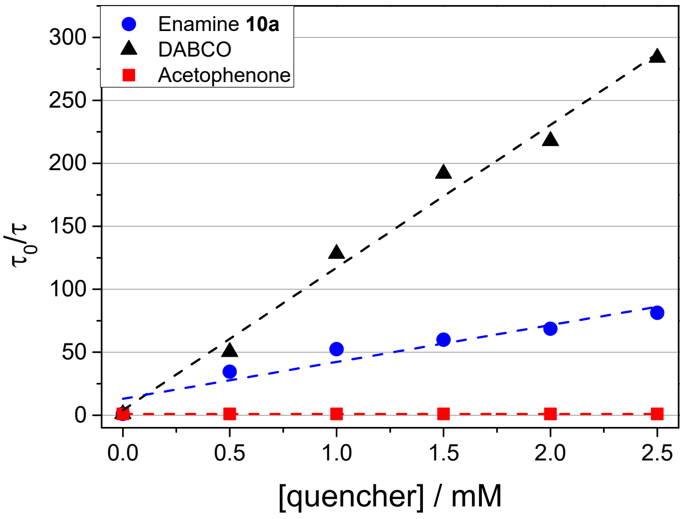

Table S6. Stern-Volmer constants (K_{SV}) and quenching rate constants (k_q) of **5CzBN** in MeCN.

Quencher	K_{SV} (M^{-1})	k_q (s^{-1} M^{-1})
Enamine **10a**	29219	$4.13 \cdot 10^9$
DABCO	113202	$1.59 \cdot 10^{10}$
Acetophenone	3.4	$4.91 \cdot 10^5$

Figure S4. Stern-Volmer plots of prompt lifetimes (excitation at 375 nm) in the presence of DABCO for 0.01 mM of a) **3DPAFIPN** in DME (emission at 532 nm), b) **5CzBN** in DMPU (emission at 520 nm) and c) **5CzBN** in MeCN (emission at 520 nm).

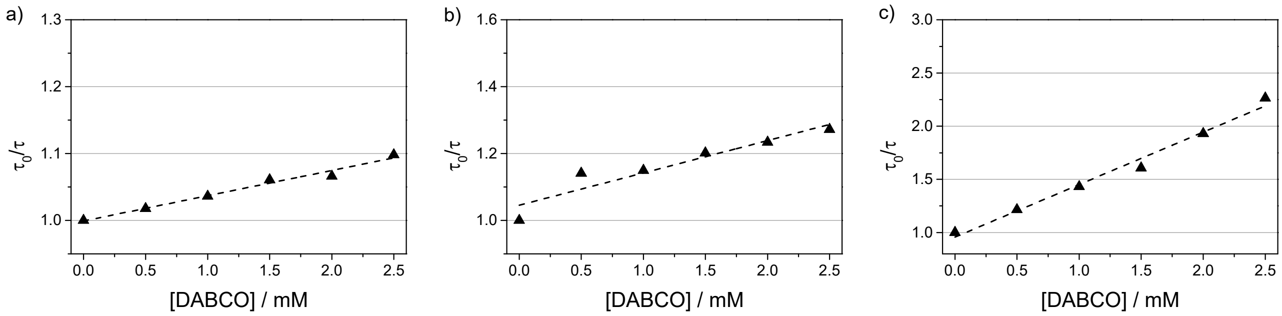

Table S7. Comparison between quenching of delayed and prompt fluorescence of the employed dyes by DABCO.

Quenching constant by DABCO		
DYE/solvent	k_q delayed fluorescence ($s^{-1}M^{-1}$)	k_q prompt fluorescence ($s^{-1}M^{-1}$)
**3DPAFIPN**/DME	$7.71 \cdot 10^8$	$9.35 \cdot 10^9$
**5CzBN**/DMPU	$1.80 \cdot 10^9$	$3.48 \cdot 10^9$
**5CzBN**/MeCN	$1.59 \cdot 10^{10}$	$1.98 \cdot 10^{10}$

Catalytic cycle proposed for the *β*-aldol reaction of cyclic ketones with aryl-alkyl ketones

Scheme S1. Proposed catalytic cycle, involving DABCO-mediated oxidation of the enamine.

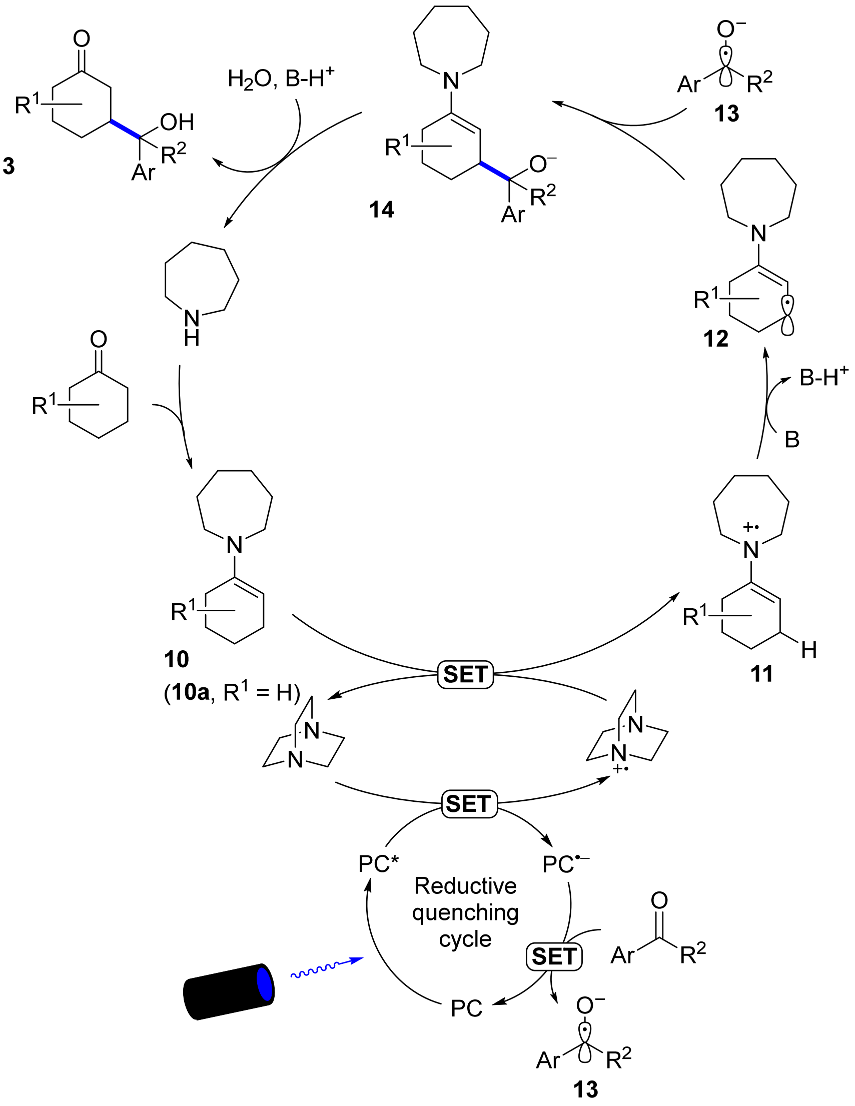

Reaction setup

The magnetically stirred reactions were run at room temperature in 10 mL microwave vials sealed with a septum under irradiation from one 40 W Kessil PR160L lamp (λ = 427 nm; distance between lamp and vial(s): 5 cm). A fan was used to dissipate the heat generated by the lamp.

Figure S5. Reaction setup.

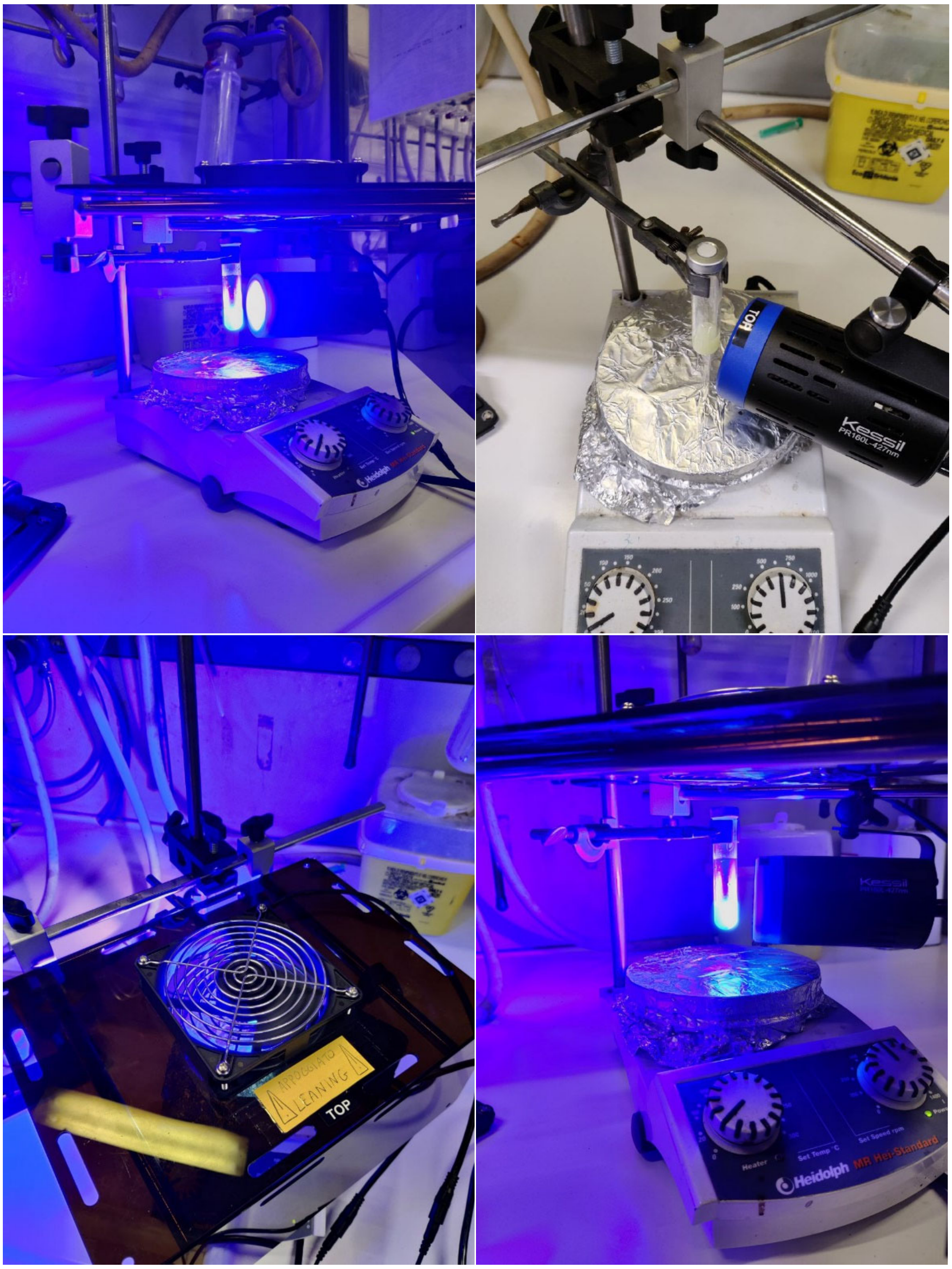

General Procedure for the reactions

General Procedure 1: *β*-Michael of aldehydes

To a vial containing a stirring bar DABCO (28.0 mg, 0.25 mmol, 1 eq) and **3DPAFIPN** (3.2 mg, 0.005 mmol, 2 mol%) were added. The vial was sealed and put under N_2. A 0.5 M solution of acrylate **1** (0.25 mmol, 1 eq, 0.50 ml) in DME was added, followed by dicyclohexylamine (10 µl, 0.05 mmol, 20 mol%), water (14 µl, 0.75 mmol, 3 eq), trifluoroacetic acid (5 µl, 0.05 mmol, 20 mol%) and aldehyde (0.50 mmol, 2 eq). The vial was then cooled into a liquid nitrogen bath and degassed via vacuum evacuation. The reaction vial was backfilled with N_2 and warmed to room temperature. This purge-and-backfill procedure was repeated three times. The reaction vial was then placed at ca. 2 cm from a 427 nm Kessil lamp (40 W) and stirred under a nitrogen atmosphere at room temperature (a fan was used to dissipate the heat generated by the lamp). After 16 h, the reaction was quenched with EtOAc and concentrated in vacuo. The crude was purified by column chromatography on silica gel (eluent: 95:5 to 8:2 hexane/diethyl ether) to afford the pure product.

General Procedure 2: *β*-aldol reaction of cyclic ketones and aryl-aryl ketones

To a vial containing a stirring bar **5CzBN** (7.0 mg, 7.5 µmol, 2 mol%), DABCO (84.1 mg, 0.75 mmol, 2 eq), $LiAsF_6$ (73.4 mg, 0.375 mmol, 1 eq) and the aryl-aryl ketone (0.375 mmol, 1 eq) were added. The vial was sealed and put under N_2. Azepane (9 µl, 0.075 mmol, 20 mol%), the cyclic ketone (1.85 mmol, 5 eq), acetic acid (5 µl, 0.075 mmol, 0.2 eq), water (14 µl, 0.75 mmol, 2 eq) and DMPU (0.75 ml, $C_{0,\text{sub.}}$ = 0.5 M) were added to the reaction vial. The vial was then cooled into a liquid nitrogen bath and degassed via vacuum evacuation. The reaction vial was backfilled with N_2 and warmed to room temperature. This purge-and-backfill procedure was repeated three times. The reaction vial was then placed ca. 2 cm from a 427 nm Kessil lamp (40 W) and stirred under a nitrogen atmosphere at room temperature (a fan was used to dissipate the heat generated by the lamp). After 16 h, the reaction mixture was diluted with EtOAc and then washed with brine, water and brine. The combined aqueous washings were extracted three times with EtOAc. The combined organic extracts were dried over Na_2SO_4, filtered and concentrated in vacuo. The crude was purified by column chromatography on silica gel (eluent: 9:1 to 6:4 hexane/ethyl acetate) to afford the pure product.

General Procedure 3: *β*-aldol reaction of cyclic ketones and alkyl-aryl ketones

To a vial containing a stirring bar **3CzClIPN** (4.9 mg, 7.5 µmol, 2 mol%) and DABCO (126.2 mg, 1.13 mmol, 3 eq) were added. The vial was sealed and put under N_2. The alkyl-aryl ketone (0.375 mmol, 1 eq), azepane (17 µl, 0.15 mmol, 40 mol%), the cyclic ketone (3.70 mmol, 10 eq), acetic acid (9 µl, 0.15 mmol, 0.4 eq), water (14 µl, 0.75 mmol, 2 eq) and MeCN (2.2 ml, $C_{0,\text{sub.}}$ = 0.17 M) were added to the reaction vial. The vial was then cooled into a liquid nitrogen bath and degassed via vacuum evacuation. The reaction vial was backfilled with N_2 and warmed to room temperature. This purge-and-backfill procedure was repeated three times. The reaction vial was then placed about 2 cm from a 427 nm Kessil lamp (40 W) and stirred under a nitrogen atmosphere, without employing a cooling fan. After 16 h, the reaction was quenched with ethyl acetate and concentrated in vacuo. The crude was purified by column chromatography on silica gel (eluent: 9:1 to 6:4 hexane/ethyl acetate) to afford the pure product.

General Procedure 4: *β*-Mannich reaction of cyclic ketones

To a vial containing a stirring bar **5CzBN** (9.4 mg, 10 µmol, 2 mol%) and DABCO (56.2 mg, 0.50 mmol, 1 eq) were added. The vial was sealed and put under N_2. A 0.75 M solution of imine **4** (0.50 mmol, 1 eq, 0.67 ml) in DMPU, the cyclic ketone (2.50 mmol, 5 eq), azepane (12 µl, 100 µmol, 20 mol%) and trifluoroacetic acid (8 µl, 200 µmol, 0.2 eq) were added to the reaction vial. The vial was then cooled into a liquid nitrogen bath and degassed via vacuum evacuation. The reaction vial was backfilled with N_2 and warmed to room temperature. This purge-

and-backfill procedure was repeated three times. The reaction vial was then placed about 2 cm from a 427 nm Kessil lamp (40 W) and stirred under a nitrogen atmosphere at room temperature (a fan was used to dissipate the heat generated by the lamp). After 16 h, the reaction mixture was diluted with EtOAc and then washed with brine, water and brine. The combined aqueous washings were extracted three times with EtOAc. The combined organic extracts were dried over Na_2SO_4, filtered and concentrated in vacuo. The crude was purified by column chromatography on silica gel (eluent: 95:5 to 8:2 hexane/ethyl acetate) to afford the pure product.

Synthesis of *β*-Michael products

Benzyl 4-(2-oxoethyl)-2-phenylnonanoate (**2a**)[9]

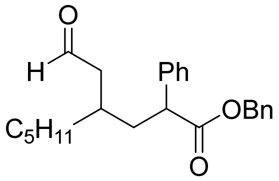

General Procedure 1 was followed using DABCO (28.0 mg, 0.25 mmol, 1 eq), **3DPAFIPN** (3.2 mg, 0.005 mmol, 2 mol%), benzyl 2-phenylacrylate[10] (59.6 mg, 0.25 mmol, 1 eq), dicyclohexylamine (10 µl, 0.05 mmol, 20 mol%), water (14 µl, 0.75 mmol, 3 eq), trifluoroacetic acid (5 µl, 0.05 mmol, 20 mol%) and octanal (78 µl, 0.50 mmol, 2 eq) in DME (0.50 ml) for 16 hours. Purification by flash chromatography (9:1 hexane/diethyl ether) afforded compound **2a** as a yellowish oil (~ 1:1 mixture of diastereoisomers; yield: 44.4 mg; 49%).

^{1}H NMR (400 MHz, $CDCl_3$): δ 9.64 (t, *J* = 2.4 Hz, 0.5H, diast. 1), 9.61 (t, *J* = 2.2 Hz, 0.5H, diast. 2), 7.38-7.17 (m, 10H), 5.14 (d, *J* = 12.4 Hz, 1H), 5.05 (d, *J* = 12.4 Hz, 1H), 3.75-3.64 (m, 1H), 2.40-2.25 (m, 2H), 2.22-2.12 (m, 0.5H), 2.11-2.01 (m, 0.5H), 1.92-1.81 (m, 1.5H), 1.81-1.73 (m, 0.5H), 1.43-1.11 (m, 8H), 0.86 (m, 3H).

tert-Butyl 4-(3-(benzyloxy)-3-oxo-2-phenylpropyl)-4-(2-oxoethyl)piperidine-1-carboxylate (**2b**)[9]

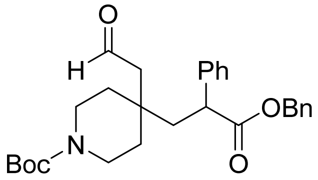

General Procedure 1 was followed using DABCO (28.0 mg, 0.25 mmol, 1 eq), **3DPAFIPN** (3.2 mg, 0.005 mmol, 2 mol%), benzyl 2-phenylacrylate[10] (59.6 mg, 0.25 mmol, 1 eq), dicyclohexylamine (10 µl, 0.05 mmol, 20 mol%), water (14 µl, 0.75 mmol, 3 eq), acetic acid (3 µl, 0.05 mmol, 20 mol%) and *N*-Boc-4-piperidineacetaldehyde (113.7 mg, 0.50 mmol, 2 eq) in DME (0.50 ml) for 16 hours. Purification by flash chromatography (85:15 hexane/acetone) afforded compound **2b** as a yellowish oil (yield: 68.2 mg; 59%).

^{1}H NMR (400 MHz, $CDCl_3$): δ 9.68 (t, *J* = 2.4 Hz, 1H), 7.43-7.15 (m, 10H), 5.15 (d, *J* = 12.3 Hz, 1H), 5.00 (d, *J* = 12.3 Hz, 1H), 3.72 (dd, *J* = 8.5, 4.4 Hz, 1H), 3.52-3.33 (m, 2H), 3.34-3.17 (m, 2H), 2.56 (dd, *J* = 14.8, 8.5 Hz, 1H), 2.38-2.33 (m, 2H), 1.92 (dd, *J* = 14.8, 4.4 Hz, 1H), 1.46-1.42 (m, 13H).

Benzyl 4-cyclohexyl-6-oxo-2-phenylhexanoate (**2c**)[9]

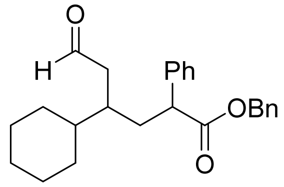

General Procedure 1 was followed using DABCO (28.0 mg, 0.25 mmol, 1 eq), **3DPAFIPN** (3.2 mg, 0.005 mmol, 2 mol%), benzyl 2-phenylacrylate[10] (59.6 mg, 0.25 mmol, 1 eq), dicyclohexylamine (10 µl, 0.05 mmol, 20 mol%), water (14 µl, 0.75 mmol, 3 eq), trifluoroacetic acid (5 µl, 0.05 mmol, 20 mol%) and 3-cyclohexylpropionaldehyde[11] (85 µl, 0.50 mmol, 2 eq) in DME (0.50 ml) for 16 hours. Purification by flash chromatography (9:1 hexane/diethyl ether) afforded compound **2c** as a yellowish oil (~ 1:1 mixture of diastereoisomers; yield: 54.6 mg; 58%).

^{1}H NMR (400 MHz, $CDCl_3$): δ 9.60 (t, *J* = 2.4 Hz, 0.5H, diast. 1), 9.57 (t, *J* = 2.2 Hz, 0.5H, diast. 2), 7.36-7.19 (m, 10H), 5.14 (d, *J* = 12.4 Hz, 1H), 5.05 (dd, *J* = 12.4, 7.2 Hz, 1H), 3.69-3.61 (m, 1H), 2.45-2.33 (m, 1H), 2.29-2.19 (m, 1H), 1.99-1.93 (m, 1H), 1.85-1.58 (m, 5H), 1.55-1.40 (m, 1.5H), 1.40-0.88 (m, 6.5H).

Benzyl 3-(1-(2-oxoethyl)cyclohexyl)-2-phenylpropanoate (**2d**)[9]

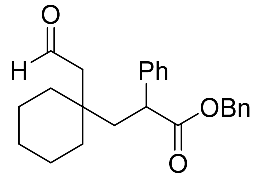

General Procedure 1 was followed using DABCO (28.0 mg, 0.25 mmol, 1 eq), **3DPAFIPN** (3.2 mg, 0.005 mmol, 2 mol%), benzyl 2-phenylacrylate[10] (59.6 mg, 0.25 mmol, 1 eq), dicyclohexylamine (10 µl, 0.05 mmol, 20 mol%), water (14 µl, 0.75 mmol, 3 eq), trifluoroacetic acid (5 µl, 0.05 mmol, 20 mol%) and 2-cyclohexylacetaldehyde[12] (69 µl, 0.50 mmol, 2 eq) in DME (0.50 ml) for 16 hours. Purification by flash chromatography (9:1 hexane/diethyl ether) afforded compound **2d** as a yellowish oil (yield: 34.9 mg; 38%).

^{1}H NMR (400 MHz, $CDCl_3$): δ 9.71 (t, *J* = 2.9 Hz, 1H), 7.36-7.26 (m, 7H), 7.25-7.19 (m, 3H), 5.14 (d, *J* = 12.3 Hz, 1H), 5.00 (d, *J* = 12.3 Hz, 1H), 3.75 (dd, *J* = 8.8, 4.1 Hz, 1H), 2.52 (dd, *J* = 14.7, 8.8 Hz, 1H), 2.34 (dd, *J* = 15.2, 2.7 Hz, 1H), 2.27 (dd, *J* = 15.2, 3.2 Hz, 1H), 1.85 (dd, *J* = 14.7, 4.1 Hz, 1H), 1.53-1.21 (m, 10H).

Methyl 4-(2-oxoethyl)-2-phenylnonanoate (**2e**)[9]

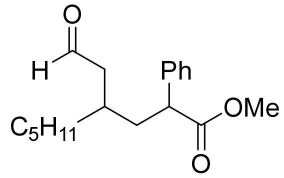

General Procedure 1 was followed using DABCO (28.0 mg, 0.25 mmol, 1 eq), **3DPAFIPN** (3.2 mg, 0.005 mmol, 2 mol%), methyl 2-phenylacrylate[13] (40.5 mg, 0.25 mmol, 1 eq), dicyclohexylamine (10 µl, 0.05 mmol, 20 mol%), water (14 µl, 0.75 mmol, 3 eq), trifluoroacetic acid (5 µl, 0.05 mmol, 20 mol%) and octanal (78 µl, 0.50 mmol, 2 eq) in DME (0.50 ml) for 16 hours. Purification by flash chromatography (9:1 hexane/diethyl ether) afforded compound **2e** as a yellowish oil (~ 1:1 mixture of diastereoisomers; yield: 30.6 mg; 42%).

^{1}H NMR (400 MHz, $CDCl_3$): δ 9.70 (t, J = 2.3 Hz, 0.5H, diast. 1), 9.66 (t, J = 2.2 Hz, 0.5H, diast. 2), 7.37-7.23 (m, 5H), 3.65 (s, 3H), 3.65-3.61 (m, 1H), 2.34 (dt, J = 6.4, 2.4 Hz, 2H), 2.19-2.09 (m, 0.5H), 2.08-1.98 (m, 0.5H), 1.93-1.82 (m, 1.5H), 1.82-1.72 (m, 0.5H), 1.41-1.14 (m, 8H), 0.92-0.82 (m, 3H).

Methyl 2-(4-chlorophenyl)-4-(2-oxoethyl)nonanoate (**2f**)[9]

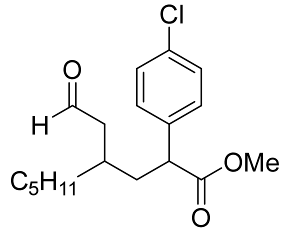

General Procedure 1 was followed using DABCO (28.0 mg, 0.25 mmol, 1 eq), **3DPAFIPN** (3.2 mg, 0.005 mmol, 2 mol%), methyl 2-(4-chlorophenyl)acrylate[14] (49.2 mg, 0.25 mmol, 1 eq), dicyclohexylamine (10 µl, 0.05 mmol, 20 mol%), water (14 µl, 0.75 mmol, 3 eq), trifluoroacetic acid (5 µl, 0.05 mmol, 20 mol%) and octanal (78 µl, 0.50 mmol, 2 eq) in DME (0.50 ml) for 16 hours. Purification by flash chromatography (85:15 hexane/diethyl ether) afforded compound **2f** as a yellowish oil (~ 1:1 mixture of diastereoisomers; yield: 29.1 mg; 36%).

^{1}H NMR (400 MHz, $CDCl_3$): δ 9.70 (t, J = 2.3 Hz, 0.5H, diast. 1), 9.68 (t, J = 2.1 Hz, 0.5H), 7.34-7.28 (m, 2H), 7.27-7.20 (m, 2H), 3.66 (s, 1.5H, diast. 1), 3.65 (s, 1.5H, diast. 2), 3.64-3.59 (m, 1H), 2.42-2.31 (m, 2H), 2.15-2.05 (m, 0.5H), 2.05-1.97 (m, 0.5H), 1.89-1.79 (m, 1.5H), 1.79-1.70 (m, 0.5H), 1.40-1.12 (m, 8H), 0.90-0.84 (m, 3H).

Methyl 2-(4-fluorophenyl)-4-(2-oxoethyl)nonanoate (**2g**)[9]

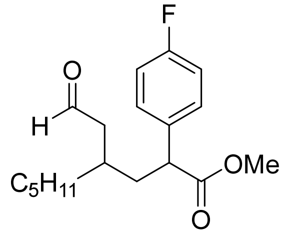

General Procedure 1 was followed using DABCO (28.0 mg, 0.25 mmol, 1 eq), **3DPAFIPN** (3.2 mg, 0.005 mmol, 2 mol%), methyl 2-(4-fluorophenyl)acrylate[14] (45.0 mg, 0.25 mmol, 1 eq), dicyclohexylamine (10 µl, 0.05 mmol, 20 mol%), water (14 µl, 0.75 mmol, 3 eq), trifluoroacetic acid (5 µl, 0.05 mmol, 20 mol%) and octanal (78 µl, 0.50 mmol, 2 eq) in DME (0.50 ml) for 16 hours. Purification by flash chromatography (9:1 hexane/diethyl ether) afforded compound **2g** as a yellowish oil (~ 1:1 mixture of diastereoisomers; yield: 30.2 mg; 39%).

^{1}H NMR (400 MHz, $CDCl_3$): δ 9.70 (t, J = 2.3 Hz, 0.5H, diast. 1), 9.68 (t, J = 2.1 Hz, 0.5H, diast. 2), 7.31-7.22 (m, 2H), 7.07-6.96 (m, 2H), 3.66 (s, 1.5H, diast. 1), 3.65 (s, 1.5H, diast. 2), 3.64-3.60 (m, 1H), 2.38-2.32 (m, 2H), 2.15-2.05 (m, 0.5H), 2.05-1.95 (m, 0.5H), 1.89-1.81 (m, 1.5H), 1.81-1.70 (m, 0.5H), 1.41-1.14 (m, 8H), 0.92-0.81 (m, 3H).

Methyl 2-(2-bromophenyl)-4-(2-oxoethyl)nonanoate (**2h**)[9]

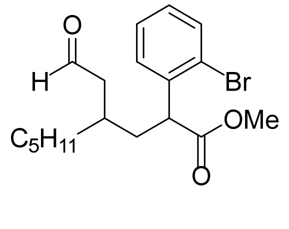

General Procedure 1 was followed using DABCO (28.0 mg, 0.25 mmol, 1 eq), **3DPAFIPN** (3.2 mg, 0.005 mmol, 2 mol%), methyl 2-(2-bromophenyl)acrylate[15] (60.3 mg, 0.25 mmol, 1 eq), dicyclohexylamine (10 µl, 0.05 mmol, 20 mol%), water (14 µl, 0.75 mmol, 3 eq), trifluoroacetic acid (5 µl, 0.05 mmol, 20 mol%) and octanal (78 µl, 0.50 mmol, 2 eq) in DME (0.50 ml) for 16 hours. Purification by flash chromatography (85:15 hexane/diethyl ether) afforded compound **2h** as a yellowish oil (~ 1:1 mixture of diastereoisomers; yield: 26.2 mg; 26%).

^{1}H NMR (400 MHz, $CDCl_3$): δ 9.72 (t, J = 2.3 Hz, 0.5H, diast. 1), 9.71 (t, J = 2.1 Hz, 0.5H, diast. 2), 7.57 (m, 1H), 7.40-7.33 (m, 1H), 7.33-7.25 (m, 1H), 7.16-7.09 (m, 1H), 4.32-4.20 (m, 1H), 3.68 (s, 3H), 2.49-2.33 (m, 2H), 2.17-2.08 (m, 0.5H), 2.06-1.96 (m, 0.5H), 1.95-1.87 (m, 1H), 1.87-1.78 (m, 0.5H), 1.76-1.66 (m, 0.5H), 1.42-1.14 (m, 8H), 0.92-0.83 (m, 3H).

Methyl 2-(4-methoxyphenyl)-4-(2-oxoethyl)nonanoate (**2i**)[9]

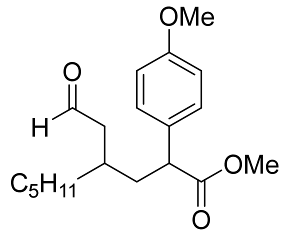

General Procedure 1 was followed using DABCO (28.0 mg, 0.25 mmol, 1 eq), **3DPAFIPN** (3.2 mg, 0.005 mmol, 2 mol%), methyl 2-(4-methoxyphenyl)acrylate[14] (48.1 mg, 0.25 mmol, 1 eq), dicyclohexylamine (10 µl, 0.05 mmol, 20 mol%), water (14 µl, 0.75 mmol, 3 eq), trifluoroacetic acid (5 µl, 0.05 mmol, 20 mol%) and octanal (78 µl, 0.50 mmol, 2 eq) in DME (0.50 ml) for 16 hours. Purification by flash chromatography (8:2 hexane/diethyl ether) afforded compound **2i** as a yellowish oil (~ 1:1 mixture of diastereoisomers; yield: 45.8 mg; 57%).

^{1}H NMR (400 MHz, $CDCl_3$): δ 9.69 (t, J = 2.4 Hz, 0.5H, diast. 1), 9.66 (t, J = 2.2 Hz, 0.5H, diast. 2), 7.26-7.17 (m, 2H), 6.88-6.81 (m, 2H), 3.79 (s, 3H), 3.65 (s, 3H), 3.63-3.56 (m, 1H), 2.37-2.31 (m, 2H), 2.15-2.05 (m, 0.5H), 2.04-1.94 (m, 0.5H), 1.90-1.80 (m, 1.5H), 1.79-1.69 (m, 0.5H), 1.40-1.12 (m, 8H), 0.92-0.81 (m, 3H).

Synthesis of *β*-aldol products from aryl-aryl ketones

3-(Hydroxydiphenylmethyl)cyclohexan-1-one (**3a**)[16]

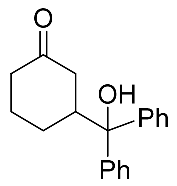

General Procedure 2 was followed using DABCO (84.1 mg, 0.75 mmol, 2 eq), **5CzBN** (7.0 mg, 0.0075 mmol, 2 mol%), $LiAsF_6$ (73.4 mg,0.375 mmol, 1 eq), benzophenone (68.3 mg, 0.375 mmol, 1 eq), azepane (9 µl, 0.075 mmol, 20 mol%), water (14 µl, 0.75 mmol, 2 eq), acetic acid (5 µl, 0.075 mmol, 20 mol%) and cyclohexanone (195 µl, 1.85 mmol, 5 eq) in DMPU (0.75 ml) for 16 hours. Purification by flash chromatography (8:2 hexane/ethyl acetate) afforded compound **3a** as a white powder (8:1 mixture with the corresponding hemiacetal; yield: 57.5 mg; 55%).

^{1}H NMR (400 MHz, $CDCl_3$): δ 7.63-7.59 (m, 0.2H, hemiacetal peak), 7.58-7.53 (m, 0.2H, hemiacetal peak), 7.49-7.44 (m, 1.8H), 7.43-7.38 (m, 1.8H), 7.35-7.23 (m, 4H), 7.22-7.15 (m, 1.8H), 7.14-7.09 (m, 0.2H), 3.00-2.85 (m, 1H), 2.46-2.34 (m, 1H), 2.32-2.20 (m, 3H), 2.13-1.99 (m, 1H), 1.88-1.77 (m, 1H), 1.75-1.57 (m, 1H), 1.57-1.42 (m, 1H).

3-(Hydroxydiphenylmethyl)-4-methylcyclohexan-1-one (**3c**)[16]

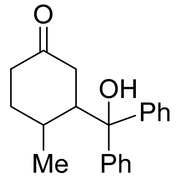

General Procedure 2 was followed using DABCO (84.1 mg, 0.75 mmol, 2 eq), **5CzBN** (7.0 mg, 0.0075 mmol, 2 mol%), $LiAsF_6$ (73.4 mg, 0.375 mmol, 1 eq), benzophenone (68.3 mg, 0.375 mmol, 1 eq), azepane (9 µl, 0.075 mmol, 20 mol%), water (14 µl, 0.75 mmol, 2 eq), acetic acid (5 µl, 0.075 mmol, 20 mol%) and 4-methylcyclohexanone (225 µl, 1.85 mmol, 5 eq) in DMPU (0.75 ml) for 16 hours. Purification by flash chromatography (85:15 hexane/ethyl acetate) afforded compound **3c** as a white powder (5:1 mixture of *trans*:*cis* diastereoisomers, each of them as ~ 3:1 mixture with the corresponding hemiacetal; yield: 51.1 mg; 46%). Determination of the d.r. and assignment of relative configuration were made by ^{1}H NMR analysis of the methyl acetal derivative **3c-acetal**, which was prepared according to the procedure reported below.[16]

^{1}H NMR (400 MHz, $CDCl_3$): δ 7.64-7.59 (m, 1.33H), 7.58-7.53 (m, 1.33H), 7.50-7.37 (m, 1.33H), 7.34-7.22 (m, 4.01H), 7.21-7.17 (m, 0.67H), 7.16-7.10 (m, 1.33H), 3.21-3.19 (m, 0.05H, hemiacetal minor diast.), 3.11-3.06 (m, 0.15H, minor diast.), 3.08-3.04 (m, 0.60H, major diast.), 2.99-2.93 (m, 0.20H, hemiacetal major diast.), 2.63 (s, 0.66H), 2.55-2.42 (m, 0.68H), 2.41-2.28 (m, 0.68H), 2.24-2.15 (m, 0.66H), 2.04-1.98 (m, 0.66H), 1.98-1.93 (m, 0.66H), 1.91-1.76 (m, 2.4H), 1.73-1.58 (m, 1H), 1.14 (d, J = 6.9 Hz, 0.35H, minor diast.), 1.10-1.03 (m, 0.6H), 1.02 (d, J = 7.2 Hz, 1.8H, major diast.), 0.94 (d, J = 7.1 Hz, 0.7H, hemiacetal major diast.), 0.46 (d, J = 7.1 Hz, 0.15H, hemiacetal minor diast.); HRMS (ESI): *m/z* calcd. for $[C_{20}H_{22}NaO_2]^+$: 317.1517 $[M+Na]^+$; found: 317.1520.

5-Methoxy-2-methyl-7,7-diphenyl-6-oxabicyclo[3.2.1]octane (**3c-acetal**)[16]

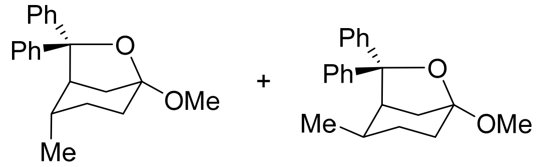

Compound **3c** (34.5 mg, 0.12 mmol, 1 eq) was dissolved in MeOH (0.47 ml), treated with *p*-TsOH•H_2O (1.7 mg, 0.009 mmol, 0.07 eq), and stirred at room temperature for 16 h. The reaction mixture was then diluted with water and extracted with ethyl acetate. The combined organic extracts were washed with brine, dried over Na_2SO_4, filtered and concentrated in vacuo. The crude was purified by column chromatography on silica gel (eluent: 97:3 to 9:1 hexane/ethyl acetate) to afford the pure methyl acetal **3c-acetal** as a yellowish oil (5:1 mixutre of *trans*:*cis* diastereoisomers; yield: 28.1 mg; 76%). Relative configuration was assigned by integration of the peaks at 3.25 ppm (*trans* isomer, known compound[16]) and 3.12 ppm (*cis* isomer) in the methyl acetal ^{1}H NMR spectrum.

^{1}H NMR (400 MHz, $CDCl_3$): δ 7.60-7.55 (m, 2H), 7.55-7.49 (m, 2H), 7.31-7.22 (m, 4H), 7.12 (tt, J = 7.4, 1.4 Hz, 2H,), 3.25 (s, 2.5H, *trans* diast.), 3.16 (m, 0.83H), 3.12 (s, 0.5H, *cis* diast.), 2.48-2.42 (m, 0.17H), 2.13–2.05 (m, 1H), 1.96-1.86 (m, 1.83H), 1.77-1.64 (m, 2.83H), 1.50-1.46 (m, 0.17H), 1.40 (d, J = 11.4 Hz, 0.17H), 1.11-1.04 (m, 1H), 1.03 (d, J = 7.2 Hz, 2.5H, *trans* diast.), 0.41 (d, J = 7.2 Hz, 0.5H, *cis* diast.); $^{13}C\{^1H\}$ NMR (101 MHz, $CDCl_3$): δ 148.5, 144.4, 128.4, 127.9, 127.5, 126.4, 126.2, 126.1, 125.7, 125.3, 125.2, 110.3, 89.3, 50.1, 49.3, 48.5 (*cis* diast.), 39.4 (*cis* diast.), 37.0 (*cis* diast.), 36.4 (*cis* diast.), 33.1, 31.0, 29.8 (*cis* diast.), 28.0, 27.8 (*cis* diast.), 25.5, 21.0 (*cis* diast.), 19.7.

3-(Hydroxydiphenylmethyl)-4-phenylcyclohexan-1-one (**3d**)

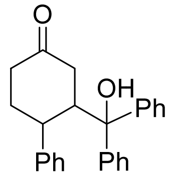

General Procedure 2 was followed using DABCO (84.1 mg, 0.75 mmol, 2 eq), **5CzBN** (7.0 mg, 0.0075 mmol, 2 mol%), $LiAsF_6$ (73.4 mg, 0.375 mmol, 1 eq), benzophenone (68.3 mg, 0.375 mmol, 1 eq), azepane (9 µl, 0.075 mmol, 20 mol%), water (14 µl, 0.75 mmol, 2 eq), acetic acid (5 µl, 0.075 mmol, 20 mol%) and 4-phenylcyclohexanone (322.3 mg, 1.85 mmol, 5 eq) in DMPU (0.75 ml) for 16 hours, without employing a cooling fan. Purification by flash chromatography (9:1 hexane/ethyl acetate) afforded compound **3d** as a pale yellow powder (20:1 mixture of *trans*:*cis* diastereoisomers, 3:1 mixture with the corresponding hemiacetal for the *trans* isomer; yield: 33.0 mg; 25%). Mp = 62-67 °C. Determination of the d.r. and assignment of relative configuration were made by ^{1}H NMR analysis of the methyl acetal derivative **3d-acetal**, which was prepared according to the procedure reported below.[16]

^{1}H NMR (400 MHz, $CDCl_3$): δ 7.74-7.61 (m, 3H), 7.43-7.00 (m, 11.3H), 6.89-6.71 (m, 0.7H), 3.65-3.55 (m, 0.25H), 3.44-3.36 (m, 0.75H), 3.21-3.13 (m, 0.75H), 3.05-2.96 (m, 0.25H, hemiacetal peak), 2.88-2.75 (m, 0.75H), 2.68 (dd, *J* = 15.4, 7.4 Hz, 0.25H), 2.57 (dd, *J* = 15.4, 4.2 Hz, 0.25H), 2.51-2.37 (m, 0.75H), 2.17-1.67 (m, 5H); ^{13}C{^{1}H} NMR (101 MHz, $CDCl_3$): δ 214.3, 148.6, 147.4 (hemiacetal peak), 145.6 (hemiacetal peak), 145.5, 145.1 (hemiacetal peak), 144.0, 128.8, 128.6, 128.5, 128.3, 128.0, 127.7, 127.3, 127.2, 126.7, 126.6, 126.5, 126.1, 126.0, 125.7, 125.0, 107.0, 89.2 (hemiacetal peak), 82.2 (hemiacetal peak), 52.2, 47.9 (hemiacetal peak), 40.8 (hemiacetal peak), 40.6 (hemiacetal peak), 38.4, 38.0 (hemiacetal peak), 36.9, 36.5, 30.8 (hemiacetal peak), 22.7; IR (ATR): ν = 3384, 3087, 3060, 3028, 2957, 2931, 2242, 1709, 1601, 1491, 1447, 1153, 990, 694 cm^{-1}; HRMS (ESI): *m/z* calcd. for $[C_{25}H_{24}NaO_2]^+$: 379.1674 $[M+Na]^+$; found: 379.1674.

5-Methoxy-2,7,7-triphenyl-6-oxabicyclo[3.2.1]octane (**3d-acetal**)[16]

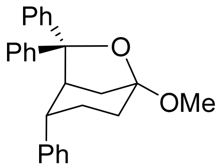

Compound **3d** (23.4 mg, 0.066 mmol, 1 eq) was dissolved in MeOH (0.87 ml), treated with *p*-TsOH•H_2O (1.0 mg, 0.0053 mmol, 0.08 eq), and stirred at room temperature for 16 h. The reaction mixture was then diluted with water and extracted with ethyl acetate. The combined organic extracts were washed with brine, dried over Na_2SO_4, filtered and concentrated in vacuo. The crude was purified by column chromatography on silica gel (eluent: 97:3 to 9:1 hexane/ethyl acetate) to afford the pure methyl acetal **3d-acetal** as a yellowish oil (20:1 mixture of *trans* and *cis* diastereoisomers; yield: 16.6 mg; 68%). Relative configuration was assigned by integration of the peaks at 3.48 ppm (*trans* isomer, known compound[16]) and 3.84 ppm (*cis* isomer) in the methyl acetal ^{1}H NMR spectrum.

^{1}H NMR (400 MHz, $CDCl_3$): δ 7.66 (d, *J* = 7.7 Hz, 2H), 7.59 (d, *J* = 8.2 Hz, 2H), 7.39-7.27 (m, 7H), 7.25–7.10 (m, 4H), 3.52-3.46 (m, 1H), 3.24 (s, 3H), 3.09-3.03 (m, 1H), 2.21-2.13 (m, 1H), 2.07-1.88 (m, 3H), 1.83-1.74 (m, 1H), 1.53 (d, *J* = 11.0 Hz, 1H); ^{13}C{^{1}H} NMR (101 MHz, $CDCl_3$): δ 148.0, 145.8, 144.4, 128.6, 128.5, 128.2, 127.8, 126.6, 126.4, 126.1, 125.7, 125.3, 110.4, 88.8, 50.9, 50.1, 39.0, 35.4, 31.2, 22.6.

3-(Hydroxydiphenylmethyl)-5-methylcyclohexan-1-one (**3e**)[16]

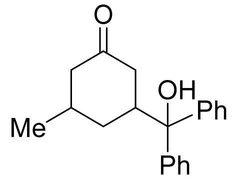

General Procedure 2 was followed using DABCO (84.1 mg, 0.75 mmol, 2 eq), **5CzBN** (7.0 mg, 0.0075 mmol, 2 mol%), $LiAsF_6$ (73.4 mg, 0.375 mmol, 1 eq), benzophenone (68.3 mg, 0.375 mmol, 1 eq), azepane (9 µl, 0.075 mmol, 20 mol%), water (14 µl, 0.75 mmol, 2 eq), acetic acid (5 µl, 0.075 mmol, 20 mol%) and 3-methylcyclohexanone (225 µl, 1.85 mmol, 5 eq) in DMPU (0.75 ml) for 16 hours. Purification by flash chromatography (85:15 hexane/ethyl acetate) afforded compound **3e** as a yellow powder (~ 1:1 mixture of diastereoisomers, one of which in mixture with the corresponding hemiacetal; yield: 24.0 mg; 22%).

^{1}H NMR (400 MHz, $CDCl_3$): δ 7.63-7.57 (m, 0.4H), 7.56-7.50 (m, 0.4H), 7.49-7.43 (m, 1.6H), 7.43-7.37 (m, 1.6H), 7.36-7.27 (m, 3H), 7.26-7.08 (m, 3H), 3.29-3.23 (m, 0.2H), 3.22-3.13 (m, 0.3H), 2.97-2.87 (m, 0.5H), 2.51-2.27 (m, 1.6H), 2.25-2.16 (m, 1.6H), 2.15-1.83 (m, 2H), 1.82-1.63 (m, 1.2H), 1.61-1.57 (m, 0.8H), 1.34-1.18 (m, 0.8H), 1.05-0.95 (m, 2.4H), 0.74 (d, J = 6.6 Hz, 0.6H); HRMS (ESI): *m/z* calcd. for $[C_{20}H_{22}NaO_2]^+$: 317.1517 $[M+Na]^+$; found: 317.1521.

3-(Hydroxydiphenylmethyl)cyclopentan-1-one (**3f**)[16]

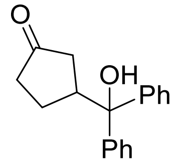

General Procedure 2 was followed using DABCO (84.1 mg, 0.75 mmol, 2 eq), **5CzBN** (7.0 mg, 0.0075 mmol, 2 mol%), $LiAsF_6$ (73.4 mg,0.375 mmol, 1 eq), benzophenone (68.3 mg, 0.375 mmol, 1 eq), azepane (9 µl, 0.075 mmol, 20 mol%), water (14 µl, 0.75 mmol, 2 eq), acetic acid (5 µl, 0.075 mmol, 20 mol%) and cyclopentanone (165 µl, 1.85 mmol, 5 eq) in DMPU (0.75 ml) for 16 hours. Purification by flash chromatography (8:2 hexane/ethyl acetate) afforded compound **3f** as a yellow oil (yield: 11.7 mg; 12%).

^{1}H NMR (400 MHz, $CDCl_3$): δ 7.49-7.44 (m, 2H), 7.44-7.39 (m, 2H), 7.37-7.27 (m, 4H), 7.26-7.18 (m, 2H), 3.44-3.33 (m, 1H), 2.40-2.12 (m, 5H), 1.95-1.83 (m, 2H).

3-((4-Chlorophenyl)(hydroxy)(phenyl)methyl)cyclohexan-1-one (**3g**)[16]

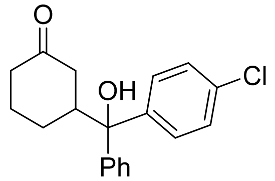

General Procedure 2 was followed using DABCO (84.1 mg, 0.75 mmol, 2 eq), **3DPA2FBN** (4.8 mg, 0.0075 mmol, 2 mol%), $LiAsF_6$ (73.4 mg, 0.375 mmol, 1 eq), 4-chlorobenzophenone (81.2 mg, 0.375 mmol, 1 eq), azepane (9 µl, 0.075 mmol, 20 mol%), water (14 µl, 0.75 mmol, 2 eq), acetic acid (5 µl, 0.075 mmol, 20 mol%) and cyclohexanone (195 µl, 1.85 mmol, 5 eq) in DMPU (0.75 ml) for 16 hours. Purification by flash chromatography (8:2 hexane/ethyl acetate) afforded compound **3g** as a white powder (1:1 mixture of diastereoisomers, each of them as mixture with the corresponding hemiacetal; yield: 61.0 mg; 52%).

^{1}H NMR (400 MHz, $CDCl_3$): δ 7.59-7.46 (m, 0.65H), 7.45-7.41 (m, 0.85H), 7.41-7.36 (m, 1.6H), 7.36-7.31 (m, 1.8H), 7.31-7.18 (m, 4.1H), 2.96-2.81 (m, 0.85H), 2.47-2.35 (m, 0.85H), 2.34-2.13 (m, 3.3H), 2.12-2.03 (m, 0.85H), 1.96-1.59 (m, 2.7H), 1.54-1.34 (m, 1.45H).

3-(Hydroxy(4-methoxyphenyl)(phenyl)methyl)cyclohexan-1-one (**3h**)[16]

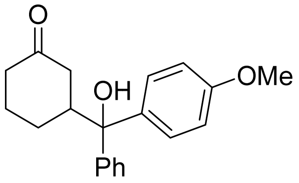

General Procedure 2 was followed using DABCO (84.1 mg, 0.75 mmol, 2 eq), **5CzBN** (7.0 mg, 0.0075 mmol, 2 mol%), $LiAsF_6$ (73.4 mg,0.375 mmol, 1 eq), 4-methoxybenzophenone (79.6 mg, 0.375 mmol, 1 eq), azepane (9 µl, 0.075 mmol, 20 mol%), water (14 µl, 0.75 mmol, 2 eq), acetic acid (5 µl, 0.075 mmol, 20 mol%) and cyclohexanone (195 µl, 1.85 mmol, 5 eq) in DMPU (0.75 ml) for 16 hours. Purification by flash chromatography (8:2 hexane/ethyl acetate) afforded compound **3h** as a white foam (1:1 mixture of diastereoisomers; yield: 40.5 mg; 35%).

^{1}H NMR (400 MHz, $CDCl_3$): δ 7.48-7.42 (m, 1H), 7.41-7.35 (m, 2H), 7.34-7.27 (m, 3H), 7.24-7.14 (m, 1H), 6.88-6.79 (m, 2H), 3.78 (s, 1.5H, diast. 1), 3.76 (s, 1.5H, diast. 2), 2.88 (br. s, 1H), 2.46-2.01 (m, 5H), 1.93-1.40 (m, 4H).

3-(9-Hydroxy-9H-xanthen-9-yl)cyclohexan-1-one (**3i**)[16]

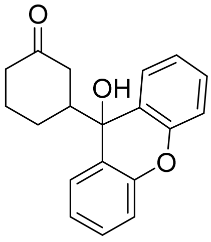

General Procedure 2 was followed using DABCO (84.1 mg, 0.75 mmol, 2 eq), **5CzBN** (7.0 mg, 0.0075 mmol, 2 mol%), $LiAsF_6$ (73.4 mg,0.375 mmol, 1 eq), xanthone (73.6 mg, 0.375 mmol, 1 eq), azepane (9 µl, 0.075 mmol, 20 mol%), water (14 µl, 0.75 mmol, 2 eq), acetic acid (5 µl, 0.075 mmol, 20 mol%) and cyclohexanone (195 µl, 1.85 mmol, 5 eq) in DMPU (0.75 ml) for 16 hours. Purification by flash chromatography (95:5 dichloromethane/ethyl acetate) afforded compound **3i** as a white powder (yield: 38.6 mg; 35%).

^{1}H NMR (400 MHz, $CDCl_3$): δ 7.68 (dd, J = 7.7, 1.7 Hz, 1H), 7.62 (dd, J = 7.8, 1.7 Hz, 1H), 7.40-7.30 (m, 2H), 7.23-7.17 (m, 2H), 7.17-7.12 (m, 2H), 2.42-2.34 (m, 1H), 2.34-2.29 (m, 1H), 2.28-2.19 (m, 1H), 2.15 (tt, J = 12.6, 3.4 Hz, 1H), 2.08-1.99 (m, 1H), 1.99-1.92 (m, 1H), 1.90 (d, J = 13.7 Hz, 1H), 1.87-1.80 (m, 1H), 1.43 (tq, J = 12.8, 3.6 Hz, 1H), 1.11 (dq, J = 13.0, 3.7 Hz, 1H).

3-(9-Hydroxy-9H-thioxanthen-9-yl)cyclohexan-1-one (**3j**)

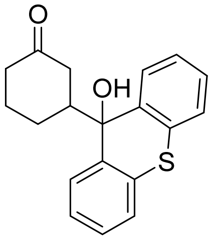

General Procedure 2 was followed using DABCO (84.1 mg, 0.75 mmol, 2 eq), **5CzBN** (7.0 mg, 0.0075 mmol, 2 mol%), $LiAsF_6$ (73.4 mg, 0.375 mmol, 1 eq), thioxanthone (79.6 mg, 0.375 mmol, 1 eq), azepane (9 µl, 0.075 mmol, 20 mol%), water (14 µl, 0.75 mmol, 2 eq), acetic acid (5 µl, 0.075 mmol, 20 mol%) and cyclohexanone (195 µl, 1.85 mmol, 5 eq) in DMPU (0.75 ml) for 16 hours. Purification by flash chromatography (8:2 hexane/ethyl acetate) afforded compound **3j** as a yellow powder (yield: 36.7 mg; 32%). Mp = 177-185 °C.

^1H NMR (400 MHz, $CDCl_3$): δ 7.79 (dd, J = 7.8, 1.5 Hz, 1H), 7.70 (dd, J = 7.8, 1.5 Hz, 1H), 7.46-7.39 (m, 2H), 7.36-7.21 (m, 4H), 2.52-2.43 (m, 1H), 2.39 (m, 1H), 2.30 (br. s, 1H), 2.27-2.12 (m, 2H), 2.00-1.91 (m, 1H), 1.90-1.84 (m, 1H), 1.65-1.54 (m, 1H), 1.40-1.28 (m, 2H); $^{13}C\{^1H\}$ NMR (101 MHz, $CDCl_3$): δ 212.7, 139.2, 138.7, 130.7, 130.5, 127.5, 127.4, 127.0, 126.4, 126.3, 126.2, 126.1, 77.0, 42.3, 41.2, 40.9, 25.2, 24.6; IR (ATR): ν = 3384, 2939, 2865, 1688, 1609, 1510, 1246, 1026 cm^{-1}; HRMS (ESI): m/z calcd. for $[C_{19}H_{18}NaO_2S]^+$: 333.0925 $[M+Na]^+$; found: 333.0928.

Synthesis of *β*-aldol products with aryl-alkyl ketones

3-(1-Hydroxy-1-phenylethyl)cyclohexan-1-one (**3b**)[16]

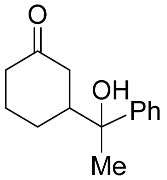

General Procedure 3 was followed using DABCO (126.2 mg, 1.125 mmol, 3 eq), **3CzClIPN** (7.0 mg, 0.0075 mmol, 2 mol%), azepane (17 µl, 0.15 mmol, 40 mol%), water (14 µl, 0.75 mmol, 2 eq), acetic acid (9 µl, 0.15 mmol, 40 mol%), acetophenone (44 µl, 0.375 mmol, 1 eq) and cyclohexanone (390 µl, 3.70 mmol, 10 eq) in MeCN (2.2 ml) for 16 hours. Purification by flash chromatography (7:3 hexane/ethyl acetate) afforded compound **3b** as a colorless oil (1:1 mixture of diastereoisomers; yield: 46.8 mg; 57%).

^1H NMR (400 MHz, $CDCl_3$): δ 7.42-7.30 (m, 4H), 7.29-7.22 (m, 1H), 2.53 (m, 0.5H), 2.40-2.28 (m, 1H), 2.28-1.96 (m, 5H), 1.73-1.62 (m, 1H), 1.61 (s, 1.5H, diast. 1), 1.56 (s, 1.5H, diast. 2), 1.54-1.33 (m, 2.5H).

3-(1-(4-Chlorophenyl)-1-hydroxyethyl)cyclohexan-1-one (**3k**)[16]

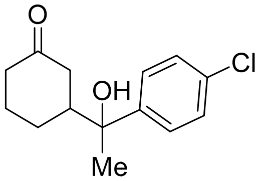

General Procedure 3 was followed using DABCO (126.2 mg, 1.125 mmol, 3 eq), **3CzClIPN** (7.0 mg, 0.0075 mmol, 2 mol%), azepane (17 µl, 0.15 mmol, 40 mol%), water (14 µl, 0.75 mmol, 2 eq), acetic acid (9 µl, 0.15 mmol, 40 mol%), 4'-chloroacetophenone (49 µl, 0.375 mmol, 1 eq) and cyclohexanone (390 µl, 3.70 mmol, 10 eq) in MeCN (2.2 ml) for 16 hours. Purification by flash chromatography (7:3 hexane/ethyl acetate) afforded compound **3k** as a colorless oil (1:1 mixture of diastereoisomers; yield: 64.9 mg; 68%).

^1H NMR (400 MHz, $CDCl_3$): δ 7.44-7.26 (m, 4H), 2.57-2.45 (m, 0.5H), 2.45-2.34 (m, 1H), 2.33-2.18 (m, 2H), 2.18-1.97 (m, 3.5H), 1.96-1.86 (m, 0.5H), 1.80-1.67 (m, 1H), 1.59 (s, 1.5H, diast. 1), 1.54 (s, 1.5H, diast. 2), 1.51-1.31 (m, 1.5H).

3-(1-Hydroxy-1-(4-methoxyphenyl)ethyl)cyclohexan-1-one (**3l**)

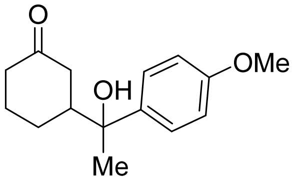

General Procedure 3 was followed using DABCO (126.2 mg, 1.125 mmol, 3 eq), **3CzClIPN** (7.0 mg, 0.0075 mmol, 2 mol%), azepane (17 µl, 0.15 mmol, 40 mol%), water (14 µl, 0.75 mmol, 2 eq), acetic acid (9 µl, 0.15 mmol, 40 mol%), 4'-methoxyacetophenone (56.3 mg, 0.375 mmol, 1 eq) and cyclohexanone (390 µl, 3.70 mmol, 10 eq) in MeCN (2.2 ml) for 16 hours. Purification by flash chromatography (93:7 dichloromethane/ethyl acetate) afforded compound **3l** as a pale brown powder (1:1 mixture of diastereoisomers; yield: 40.0 mg; 43%). Mp = 82-85 °C.

^{1}H NMR (400 MHz, $CDCl_3$): δ 7.34-7.27 (m, 2H), 6.93-6.84 (m, 2H), 3.81 (s, 1.5H, diast. 1), 3.80 (s, 1.5H, diast. 2), 2.53-2.45 (m, 0.5H), 2.36-2.28 (m, 1H), 2.26-1.97 (m, 5H), 1.72-1.65 (m, 0.5H), 1.58 (s, 1.5H, diast. 1), 1.54 (s, 1.5H, diast. 2), 1.52-1.33 (m, 2H); ^{13}C{^{1}H} NMR (101 MHz, $CDCl_3$): δ 212.8, 158.4, 138.9, 138.6, 126.3, 113.5, 75.5, 75.4, 55.3, 49.7, 49.7, 43.1, 43.0, 41.2, 41.2, 27.5, 27.4, 25.8, 25.5, 25.0; IR (ATR): ν = 3384, 2939, 2865, 2835, 1688, 1609, 1510, 1246, 1026 cm^{-1}; HRMS (ESI): *m/z* calcd. for $[C_{15}H_{20}NaO_3]^+$: 271.1310 [*M*+Na]$^+$; found: 271.1310.

3-(1-(2-(Benzyloxy)phenyl)-1-hydroxyethyl)cyclohexan-1-one (**3m**)

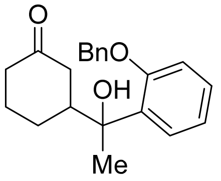

General Procedure 3 was followed using DABCO (126.2 mg, 1.125 mmol, 3 eq), **3CzClIPN** (7.0 mg, 0.0075 mmol, 2 mol%), azepane (17 µl, 0.15 mmol, 40 mol%), water (14 µl, 0.75 mmol, 2 eq), acetic acid (9 µl, 0.15 mmol, 40 mol%), 1-(2-(benzyloxy)phenyl)ethan-1-one[17] (84.8 mg, 0.375 mmol, 1 eq) and cyclohexanone (390 µl, 3.70 mmol, 10 eq) in MeCN (2.2 ml) for 16 hours. Purification by flash chromatography (7:3 hexane/ethyl acetate) afforded compound **3m** as a yellowish oil (1:1 mixture of diastereoisomers; yield: 31.4 mg; 26%).

^{1}H NMR (400 MHz, $CDCl_3$): δ 7.45-7.33 (m, 5H), 7.32-7.28 (m, 1H), 7.25-7.19 (m, 1H), 7.02-6.94 (m, 2H), 5.12 (s, 2H), 3.99 (s, 0.5H, diast. 1), 3.91 (s, 0.5H, diast. 2), 2.51-2.40 (m, 1H), 2.40-2.27 (m, 2H), 2.26-2.22 (m, 0.5H), 2.21-2.15 (m, 1H), 2.08-1.99 (m, 1H), 1.93-1.85 (m, 0.5H), 1.78-1.67 (m, 1H), 1.59 (s, 1.5H, diast. 1), 1.54 (s, 1.5H, diast. 2), 1.50-1.41 (m, 2H); ^{13}C{^{1}H} NMR (101 MHz, $CDCl_3$): δ 212.9, 212.6, 155.8, 155.8, 136.2, 134.1, 129.0, 128.5, 127.7, 127.6, 121.3, 121.3, 112.9, 77.0, 76.7, 70.8, 47.6, 43.9, 43.1, 41.5, 41.4, 26.3, 25.8, 25.4, 25.3, 24.7, 23.8; IR (ATR): ν = 3482, 2939, 2864, 1670, 1446, 1222, 730 cm^{-1}; HRMS (ESI): *m/z* calcd. for $[C_{21}H_{24}NaO_3]^+$: 347.1623 [*M*+Na]$^+$; found: 347.1624.

Synthesis of *β*-Mannich products

3-(1-((4-Methoxyphenyl)amino)-1-phenylpropyl)cyclohexan-1-one (**5a**)[18]

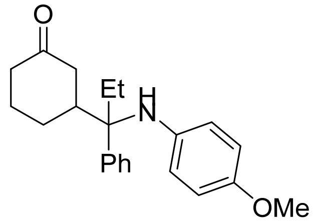

General Procedure 4 was followed using DABCO (56.1 mg, 0.50 mmol, 1 eq), **5CzBN** (9.3 mg, 0.01 mmol, 2 mol%), azepane (12 µl, 0.10 mmol, 20 mol%), trifluoroacetic acid (8 µl, 0.10 mmol, 20 mol%), imine **4a**[19] (119.7, 0.50 mmol, 1 eq) and cyclohexanone (260 µl, 2.50 mmol, 5 eq) in DMPU (0.67 ml) for 16 hours. Purification by flash chromatography (85:15 hexane/ethyl acetate) afforded compound **5a** as a white foam (1:1 mixture of diastereoisomers; yield: 144.5 mg; 86%).

^{1}H NMR (400 MHz, $CDCl_3$): δ 7.50-7.43 (m, 2H), 7.40-7.27 (m, 3H), 6.62-6.56 (m, 2H), 6.28-6.20 (m, 2H), 3.67 (s, 1.5H, diast. 1), 3.67 (s, 1.5H, diast. 2), 3.66-3.55 (m, 1H), 2.81-2.73 (m, 0.5H), 2.54-2.46 (m, 0.5H), 2.38-1.95 (m, 7H), 1.81 (t, J = 13.5 Hz, 0.5H), 1.73 (t, J = 13.4 Hz, 0.5H), 1.65-1.57 (m, 1H), 1.13-1.00 (m, 0.5H), 0.99-0.87 (m, 0.5H), 0.84-0.76 (m, 3H).

3-(((4-Methoxyphenyl)amino)(phenyl)methyl)cyclohexan-1-one (**5b**)[18]

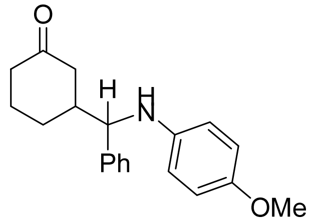

General Procedure 4 was followed using DABCO (56.1 mg, 0.50 mmol, 1 eq), **5CzBN** (9.3 mg, 0.01 mmol, 2 mol%), azepane (12 µl, 0.10 mmol, 20 mol%), trifluoroacetic acid (8 µl, 0.10 mmol, 20 mol%), imine **4b**[20] (105.6, 0.50 mmol, 1 eq) and cyclohexanone (260 µl, 2.50 mmol, 5 eq) in DMPU (0.67 ml) for 16 hours. Purification by flash chromatography (hexane/ethyl acetate) afforded compound **5b** as separate diastereoisomers (1:1 ratio; total yield: 106.5 mg; 69%).

Diastereoisomer 1 (white foam, 83:17 hexane/ethyl acetate): ^{1}H NMR (400 MHz, $CDCl_3$): δ 7.35-7.21 (m, 5H), 6.70-6.64 (m, 2H), 6.50-6.44 (m, 2H), 4.16 (d, J = 5.5 Hz, 1H), 3.86 (br. s, 1H), 3.68 (s, 3H), 2.64 (m, 1H), 2.43-1.99 (m, 5H), 1.77 (m, 1H), 1.66-1.49 (m, 1H), 1.46-1.36 (m, 1H); ^{13}C{^{1}H} NMR (101 MHz, $CDCl_3$): δ 211.2, 152.2, 141.4, 128.6, 127.4, 127.3, 114.9, 114.8, 63.6, 55.8, 45.6, 45.2, 41.4, 28.3, 25.1; HRMS (ESI): *m/z* calcd. for $[C_{20}H_{24}NO_2]^+$: 310.1807 [*M*+Na]$^+$; found: 310.1809.

Diastereoisomer 2 (white foam, 82:18 hexane/ethyl acetate): ^{1}H NMR (400 MHz, $CDCl_3$): δ 7.34-7.27 (m, 2H), 7.26-7.21 (m, 3H), 6.70-6.64 (m, 2H), 6.51-6.44 (m, 2H), 4.21 (d, J = 4.6 Hz, 1H), 3.86 (br. s, 1H), 3.68 (s, 3H), 2.41-2.03 (m, 7H), 1.70-1.58 (m, 1H), 1.51-1.41 (m, 1H); ^{13}C{^{1}H} NMR (101 MHz, $CDCl_3$): δ 211.3, 152.2, 141.4, 141.2, 128.6, 127.4, 127.3, 114.9, 63.1, 55.8, 45.6, 45.3, 41.4, 27.8, 25.1; HRMS (ESI): *m/z* calcd. for $[C_{20}H_{24}NO_2]^+$: 310.1807 [*M*+Na]$^+$; found: 310.1808; *m/z* calcd. for $[C_{20}H_{23}NNaO_2]^+$: 332.1626 [*M*+Na]$^+$; found: 332.1633.

3-(1-((4-Methoxyphenyl)amino)-1-phenylbutyl)cyclohexan-1-one (**5c**)[18]

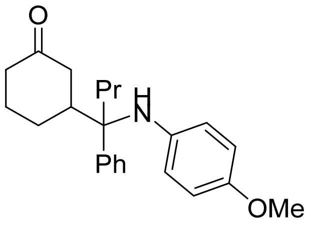

General Procedure 4 was followed using DABCO (56.1 mg, 0.50 mmol, 1 eq), **5CzBN** (9.3 mg, 0.01 mmol, 2 mol%), azepane (12 µl, 0.10 mmol, 20 mol%), trifluoroacetic acid (8 µl, 0.10 mmol, 20 mol%), imine **4c**[21] (126.7, 0.50 mmol, 1 eq) and cyclohexanone (260 µl, 2.50 mmol, 5 eq) in DMPU (0.67 ml) for 16 hours. Purification by flash chromatography (85:15 hexane/ethyl acetate) afforded compound **5c** as a yellow foam (1:1 mixture of diastereoisomers; yield: 104.2 mg; 59%).

^{1}H NMR (400 MHz, $CDCl_3$): δ 7.50-7.43 (m, 2H), 7.39-7.32 (m, 2H), 7.31-7.27 (m, 1H), 6.63-6.54 (m, 2H), 6.28-6.18 (m, 2H), 3.68 (s, 1.5H, diast. 1), 3.67 (s, 1.5H, diast. 2), 3.58 (br. s, 1H), 2.81-2.72 (m, 0.5H), 2.55-2.47 (m, 0.5H), 2.37-1.94 (m, 7H), 1.83 (t, J = 13.4 Hz, 0.5H), 1.72 (t, J = 13.5 Hz, 0.5H), 1.65-1.59 (m, 1H), 1.36-1.18 (m, 2H), 1.14-1.03 (m, 0.5H), 1.00-0.91 (m, 0.5H), 0.84-0.74 (m, 3H).

3-(1-((4-Methoxyphenyl)amino)-1,2,3,4-tetrahydronaphthalen-1-yl)cyclohexan-1-one (**5d**)[18]

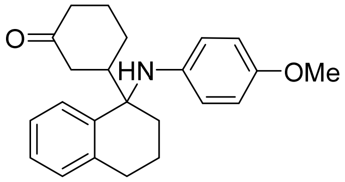

General Procedure 4 was followed using DABCO (56.1 mg, 0.50 mmol, 1 eq), **3DPAFIPN** (6.5 mg, 0.01 mmol, 2 mol%), azepane (12 µl, 0.10 mmol, 20 mol%), trifluoroacetic acid (8 µl, 0.10 mmol, 20 mol%), imine **4d**[19] (125.7, 0.50 mmol, 1 eq) and cyclohexanone (260 µl, 2.50 mmol, 5 eq) in DMPU (0.67 ml) for 16 hours. Purification by flash chromatography (8:2 hexane/ethyl acetate) afforded compound **5d** as a yellow foam (1:1 mixture of diastereoisomers; yield: 123.1 mg; 70%).

^{1}H NMR (400 MHz, $CDCl_3$): δ 7.44-7.37 (m, 1H), 7.18-7.08 (m, 3H), 6.63-6.57 (m, 2H), 6.35-6.27 (m, 2H), 3.68 (s, 1.5H, diast. 1), 3.67 (s, 1.5H, diast. 2), 2.88-2.65 (m, 2H), 2.63-2.53 (m, 0.5H), 2.44-2.02 (m, 7.5H), 1.95-1.79 (m, 2H), 1.78-1.42 (m, 4H).

3-(Phenyl(p-tolylamino)methyl)cyclohexan-1-one (**5e**)[18]

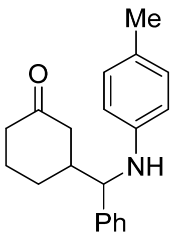

General Procedure 4 was followed using DABCO (56.1 mg, 0.50 mmol, 1 eq), **5CzBN** (9.3 mg, 0.01 mmol, 2 mol%), azepane (12 µl, 0.10 mmol, 20 mol%), trifluoroacetic acid (8 µl, 0.10 mmol, 20 mol%), imine **4e**[22] (97.6, 0.50 mmol, 1 eq) and cyclohexanone (260 µl, 2.50 mmol, 5 eq) in DMPU (0.67 ml) for 16 hours. Purification by

flash chromatography (hexane/ethyl acetate) afforded compound **5e** as separate diastereoisomers (1:1 ratio; total yield: 75.7 mg; 52%).

Diastereoisomer 1 (white foam, 9:1 hexane/ethyl acetate): ^{1}H NMR (400 MHz, $CDCl_3$): δ 7.36-7.19 (m, 5H), 6.88 (d, *J* = 8.1 Hz, 2H), 6.44 (d, *J* = 8.4 Hz, 2H), 4.21 (d, *J* = 5.4 Hz, 1H), 3.95 (br. s, 1H), 2.68-2.59 (m, 1H), 2.43-2.19 (m, 4H), 2.17 (s, 3H), 2.15-2.13 (m, 1H), 2.10-2.01 (m, 1H), 1.82-1.71 (m, 1H), 1.48-1.36 (m, 1H); ^{13}C{^{1}H} NMR (101 MHz, $CDCl_3$) δ 211.1, 144.9, 141.4, 129.7, 128.6, 127.4, 127.3, 126.9, 113.7, 63.0, 45.6, 45.2, 41.4, 28.4, 25.2, 20.4; HRMS (ESI): *m/z* calcd. for $[C_{20}H_{24}NO]^+$: 294.1858 [*M*+Na]$^+$; found: 294.1858; *m/z* calcd. for $[C_{20}H_{23}NNaO]^+$: 316.1677 [*M*+Na]$^+$; found: 316.1677.

Diastereoisomer 2 (yellow powder, 9:1 hexane/ethyl acetate): ^{1}H NMR (400 MHz, $CDCl_3$): δ 7.34-7.20 (m, 5H), 6.89 (d, *J* = 8.2 Hz, 2H), 6.44 (d, *J* = 8.4 Hz, 2H), 4.26 (d, *J* = 4.7 Hz, 1H), 4.00 (br. s, 1H), 2.40-2.20 (m, 4H), 2.17 (s, 3H), 2.14-2.03 (m, 2H), 1.70-1.41 (m, 3H); ^{13}C{^{1}H} NMR (101 MHz, $CDCl_3$): δ 211.3, 144.9, 141.2, 129.8, 128.6, 127.3, 127.2, 126.9, 113.7, 62.4, 45.6, 45.3, 41.4, 27.8, 25.1, 20.4; HRMS (ESI): *m/z* calcd. for $[C_{20}H_{24}NO]^+$: 294.1858 [*M*+Na]$^+$; found: 294.1858; *m/z* calcd. for $[C_{20}H_{23}NNaO]^+$: 316.1677 [*M*+Na]$^+$; found: 316.1680.

3-(Phenyl(phenylamino)methyl)cyclohexan-1-one (**5f**)[18]

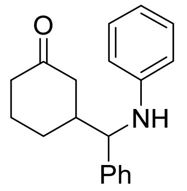

General Procedure 4 was followed using DABCO (56.1 mg, 0.50 mmol, 1 eq), **5CzBN** (9.3 mg, 0.01 mmol, 2 mol%), azepane (12 µl, 0.10 mmol, 20 mol%), trifluoroacetic acid (8 µl, 0.10 mmol, 20 mol%), imine **4f**[19] (90.6, 0.50 mmol, 1 eq) and cyclohexanone (260 µl, 2.50 mmol, 5 eq) in DMPU (0.67 ml) for 16 hours. Purification by flash chromatography (hexane/ethyl acetate) afforded compound **5f** as separate diastereoisomers (1:1 ratio; total yield: 57.4 mg; 41%).

Diastereoisomer 1 – Fraction 1 (yellow foam, 9:1 hexane/ethyl acetate): ^{1}H NMR (400 MHz, $CDCl_3$): δ 7.37-7.21 (m, 5H), 7.12-7.03 (m, 2H), 6.67-6.60 (m, 1H), 6.56-6.48 (m, 2H), 4.24 (d, *J* = 5.4 Hz, 1H), 4.09 (br. s, 1H), 2.69-2.61 (m, 1H), 2.43-2.01 (m, 6H), 1.81-1.71 (m, 1H), 1.48-1.35 (m, 1H); ^{13}C{^{1}H} NMR (101 MHz, $CDCl_3$): δ 211.1, 147.2, 141.2, 129.2, 128.7, 127.5, 127.3, 117.7, 113.6, 62.7, 45.5, 45.2, 41.4, 28.4, 25.1; HRMS (ESI): *m/z* calcd. for $[C_{19}H_{21}NNaO]^+$: 302.1521 [*M*+Na]$^+$; found: 302.1522.

Diastereoisomer 2 – Fraction 2 (yellow powder, 9:1 hexane/ethyl acetate): ^{1}H NMR (400 MHz, $CDCl_3$): δ 7.35-7.21 (m, 5H), 7.12-7.04 (m, 2H), 6.68-6.61 (m, 1H), 6.56-6.48 (m, 2H), 4.29 (d, *J* = 4.8 Hz, 1H), 4.13 (br. s, 1H), 2.42-2.04 (m, 7H), 1.70-1.59 (m, 1H), 1.53-1.43 (m, 1H); ^{13}C{^{1}H} NMR (101 MHz, $CDCl_3$): δ 211.2, 147.2, 140.9, 129.3, 128.7, 127.5, 127.2, 117.7, 113.6, 62.2, 45.5, 45.3, 41.4, 27.8, 25.1; HRMS (ESI): *m/z* calcd. for $[C_{19}H_{21}NNaO]^+$: 302.1521 [*M*+Na]$^+$; found: 302.1525.

3-(1-(Cyclohexylamino)-1-phenylpropyl)cyclohexan-1-one (**5g**)[18]

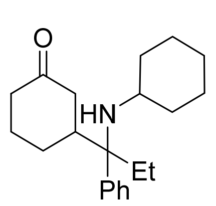

General Procedure 4 was followed using DABCO (56.1 mg, 0.50 mmol, 1 eq), **5CzBN** (9.3 mg, 0.01 mmol, 2 mol%), azepane (12 µl, 0.10 mmol, 20 mol%), trifluoroacetic acid (8 µl, 0.10 mmol, 20 mol%), imine **4g**[23] (107.7,

0.50 mmol, 1 eq) and cyclohexanone (260 µl, 2.50 mmol, 5 eq) in DMPU (0.67 ml) for 16 hours. Purification by flash chromatography (9:1 hexane/ethyl acetate) afforded compound **5g** as a colorless oil (1:1 mixture of diastereoisomers; yield: 65.5 mg; 42%).

^{1}H NMR (400 MHz, $CDCl_3$): δ 7.53-7.44 (m, 2H), 7.36-7.27 (m, 2H), 7.27-7.18 (m, 1H), 2.88-2.78 (m, 0.5H), 2.49-2.38 (m, 1.5H), 2.34 (t, J = 6.7 Hz, 2H), 2.30-2.17 (m, 1H), 2.18-2.06 (m, 0.5H), 2.07-1.81 (m, 5.5H), 1.77-1.63 (m, 4H), 1.65-1.43 (m, 2H), 1.31-1.01 (m, 6H), 0.86-0.76 (m, 3H).

3-((tert-Butylamino)diphenylmethyl)cyclohexan-1-one (**5h**)[18]

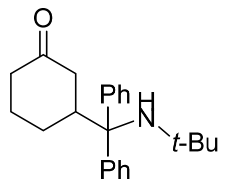

General Procedure 4 was followed using DABCO (56.1 mg, 0.50 mmol, 1 eq), **5CzBN** (9.3 mg, 0.01 mmol, 2 mol%), azepane (12 µl, 0.10 mmol, 20 mol%), trifluoroacetic acid (8 µl, 0.10 mmol, 20 mol%), imine **4h**[24] (118.7, 0.50 mmol, 1 eq) and cyclohexanone (260 µl, 2.50 mmol, 5 eq) in DMPU (0.67 ml) for 16 hours. Purification by flash chromatography (2:8 hexane/dichloromethane) afforded compound **5h** as a yellowish foam (yield: 117.7 mg; 70%).

^{1}H NMR (400 MHz, $CDCl_3$): δ 7.51-7.37 (m, 4H), 7.35-7.18 (m, 6H), 3.01-2.77 (m, 2H), 2.38-2.18 (m, 2H), 2.07-1.93 (m, 2H), 1.75-1.55 (m, 3H), 0.89-0.70 (m, 10H).

3-(1-((4-Methoxyphenyl)amino)-1-phenylpropyl)-4-methylcyclohexan-1-one (**5i**)

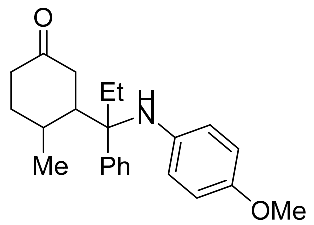

General Procedure 4 was followed using DABCO (56.1 mg, 0.50 mmol, 1 eq), **5CzBN** (9.3 mg, 0.01 mmol, 2 mol%), $LiBF_4$ (46.9 mg, 0.50 mmol, 1 eq), azepane (24 µl, 0.20 mmol, 40 mol%), trifluoroacetic acid (8 µl, 0.10 mmol, 20 mol%), imine **4a**[19] (119.7, 0.50 mmol, 1 eq) and 4-methylcyclohexanone (610 µl, 5.0 mmol, 10 eq) in DMPU (0.67 ml) for 16 hours. Purification by flash chromatography (85:15 hexane/ethyl acetate) afforded two fractions containing, respectively, a mixture of 3 diastereoisomers of compound **5i** (Diast. 1, 2, 3 / Fraction 1) and the fourth pure diastereoisomer (Diast. 4 / Fraction 2). Total yield (4 diastereoisomers in a 1:1:1:1 ratio): 79.6 mg; 45%). Mp (Fraction 1) = Mp (Fraction 2) = 45-50 °C.

Fraction 1 (diastereoisomers 1, 2, 3 as 1:1:1 mixture, white foam): ^{1}H NMR (400 MHz, $CDCl_3$): δ 7.56-7.49 (m, 1.33H), 7.46-7.41 (m, 0.67H), 7.39-7.27 (m, 3H), 6.63-6.57 (m, 1.33H), 6.55-6.51 (m, 0.67H), 6.33-6.22 (m, 1.33H), 6.13-6.07 (m, 0.67H), 3.68 (s, 1H), 3.67 (s, 1H), 3.64 (s, 1H), 2.94-2.87 (m, 0.33H), 2.56-2.36 (m, 2H), 2.36-2.06 (m, 5.67H), 1.99-1.91 (m, 0.67H), 1.85-1.70 (m, 2H), 1.18 (d, J = 7.0 Hz, 1.33H), 1.15-0.95 (m, 1.33H), 0.82-0.71 (m, 2.67H), 0.68-0.63 (m, 1H); ^{13}C{^{1}H} NMR (101 MHz, $CDCl_3$): δ 213.6, 212.3, 211.3, 151.8, 151.8, 151.7, 142.7, 142.6, 141.3, 139.9, 139.8, 139.4, 128.8, 128.2, 128.0, 128.0, 127.9, 127.1, 126.9, 126.8, 116.8, 116.4, 114.3, 64.8, 63.8, 62.6, 55.5, 49.7, 49.1, 48.2, 40.4, 39.8, 39.0, 36.4, 36.2, 34.7, 34.4, 29.7, 28.8, 28.5, 27.9, 27.0, 26.7, 24.8, 23.7, 23.6, 12.8, 12.1, 8.2, 7.5, 7.1; IR (ATR): ν = 3379, 2939, 2865, 1688, 1609, 1509, 1245, 1026, 827 cm^{-1}; HRMS (ESI): *m/z* calcd. for $[C_{23}H_{29}NNaO_2]^+$: 374.2096 $[M+Na]^+$; found: 374.2095.

Fraction 2 (pure diastereoisomer 4, white foam): ^{1}H NMR (400 MHz, $CDCl_3$): δ 7.54-7.48 (m, 2H), 7.38-7.26 (m, 3H), 6.61-6.55 (m, 2H), 6.25-6.19 (m, 2H), 3.67 (s, 3H), 3.53 (br. s, 1H), 2.68-2.59 (m, 1H), 2.57-2.48 (m, 1H), 2.38-2.27 (m, 2H), 2.13-2.01 (m, 3H), 1.56-1.48 (m, 1H), 1.32-1.25 (m, 1H), 1.24-1.12 (m, 1H), 1.08 (d, J = 6.8 Hz, 3H), 0.84 (t, J = 7.2 Hz, 3H); ^{13}C{^{1}H} NMR (101 MHz, $CDCl_3$): δ 214.8, 152.0, 141.8, 139.6, 128.3, 128.3, 127.2, 116.8, 114.4, 64.0, 55.6, 47.0, 39.1, 35.5, 27.9, 27.1, 24.3, 22.5, 7.9; IR (ATR): ν = 3368, 2932, 1705, 1508, 1234, 1034 cm^{-1}; HRMS (ESI): *m/z* calcd. for $[C_{23}H_{29}NNaO_2]^+$: 374.2096 $[M+Na]^+$; found: 374.2095.

3-(1-((4-Methoxyphenyl)amino)-1-phenylpropyl)-5-methylcyclohexan-1-one (**5j**)[18]

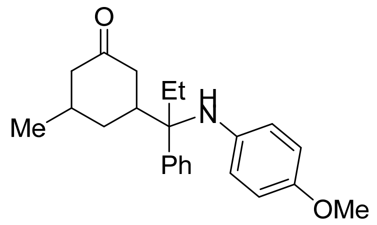

General Procedure 4 was followed using DABCO (56.1 mg, 0.50 mmol, 1 eq), **5CzBN** (9.3 mg, 0.01 mmol, 2 mol%), $LiBF_4$ (46.9 mg, 0.50 mmol, 1 eq), azepane (24 µl, 0.20 mmol, 40 mol%), trifluoroacetic acid (8 µl, 0.10 mmol, 20 mol%), imine **4a**[19] (119.7, 0.50 mmol, 1 eq) and 3-methylcyclohexanone (610 µl, 5.0 mmol, 10 eq) in DMPU (0.67 ml) for 16 hours. Purification by flash chromatography (85:15 hexane/ethyl acetate) afforded compound **5j** as a yellowish oil (1:1 mixture of diastereoisomers; yield: 94.1 mg; 54%).

^{1}H NMR (400 MHz, $CDCl_3$): δ 7.49-7.43 (m, 2H), 7.40-7.33 (m, 2H), 7.33-7.28 (m, 1H), 6.63-6.55 (m, 2H), 6.30-6.19 (m, 2H), 3.68 (s, 1.5H), 3.67 (s, 1.5H), 3.60 (br. s, 1H), 2.78-2.68 (m, 0.5H), 2.51-2.41 (m, 0.5H), 2.37-2.20 (m, 3.5H), 2.16-2.04 (m, 1.5H), 2.03-2.00 (m, 0.5H), 1.97-1.91 (m, 0.5H), 1.82-1.67 (m, 2.5H), 1.40-1.29 (m, 0.5H), 1.04-0.98 (m, 3H), 0.84-0.77 (m, 3H).

3-(1-((4-Methoxyphenyl)amino)-1-phenylpropyl)cyclopentan-1-one (**5k**)[18]

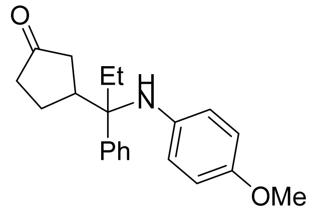

General Procedure 4 was followed using DABCO (56.1 mg, 0.50 mmol, 1 eq), **5CzBN** (9.3 mg, 0.01 mmol, 2 mol%), $LiBF_4$ (46.9 mg, 0.50 mmol, 1 eq), morpholine (17 µl, 0.20 mmol, 40 mol%), trifluoroacetic acid (8 µl, 0.10 mmol, 20 mol%), imine **4a**[19] (119.7, 0.50 mmol, 1 eq) and cyclopentanone (440 µl, 5.0 mmol, 10 eq) in DMPU (0.67 ml) for 16 hours. Purification by flash chromatography (85:15 hexane/ethyl acetate) afforded compound **5k** as a yellowish foam (1:1 mixture of diastereoisomers; yield: 52.2 mg; 32%).

^{1}H NMR (400 MHz, $CDCl_3$): δ 7.55-7.44 (m, 2H), 7.41-7.33 (m, 2H), 7.32-7.28 (m, 1H), 6.64-6.54 (m, 2H), 6.33-6.19 (m, 2H), 3.76 (br. s, 1H), 3.68 (s, 1.5H, diast. 1), 3.67 (s, 1.5H, diast. 2), 2.62 (m, 1H), 2.49-2.13 (m, 3H), 2.13-1.78 (m, 4H), 1.51-1.39 (m, 1H), 0.96-0.78 (m, 3H).

NMR spectra of the isolated products

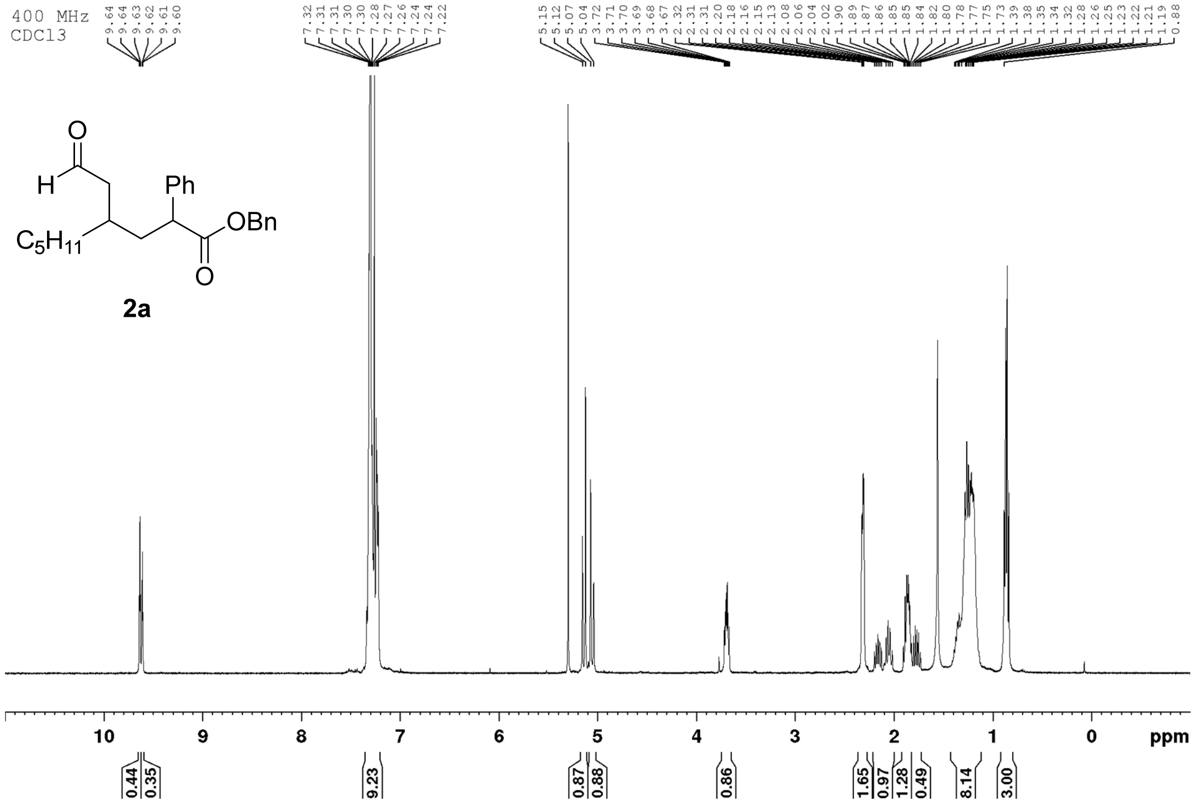

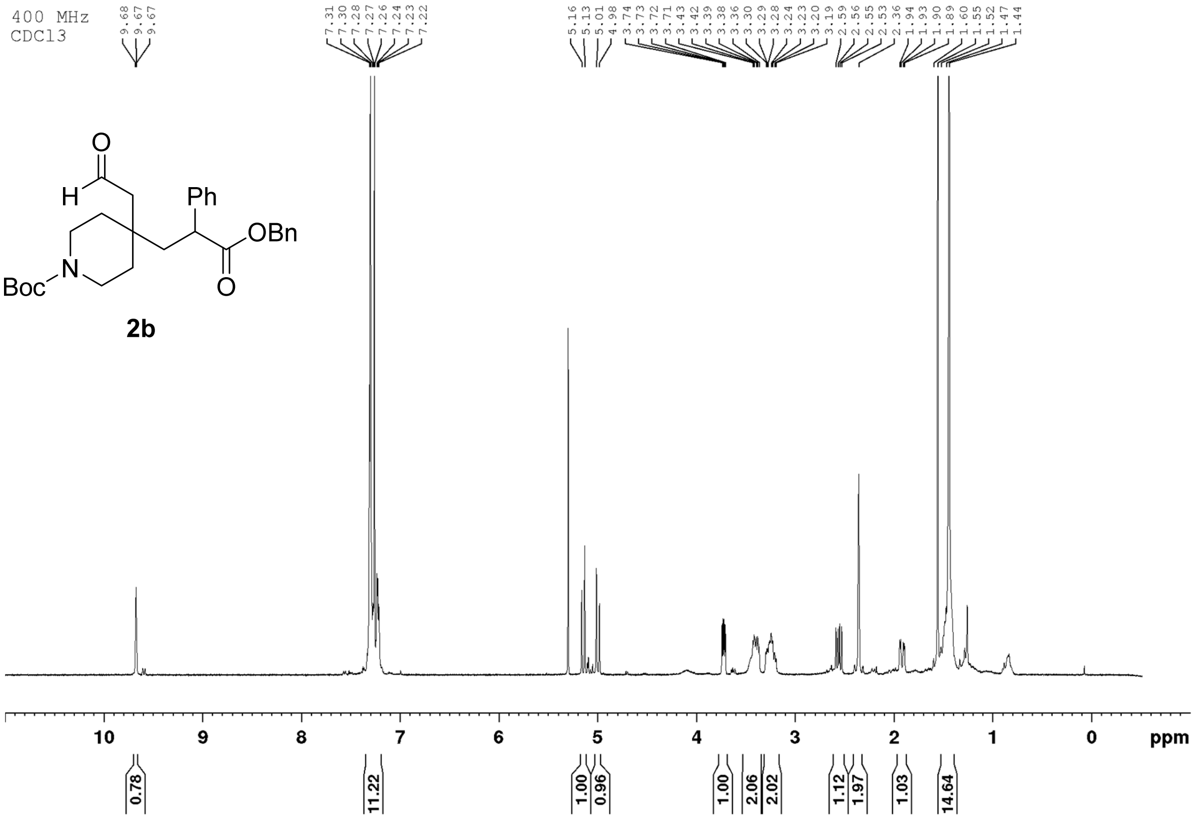

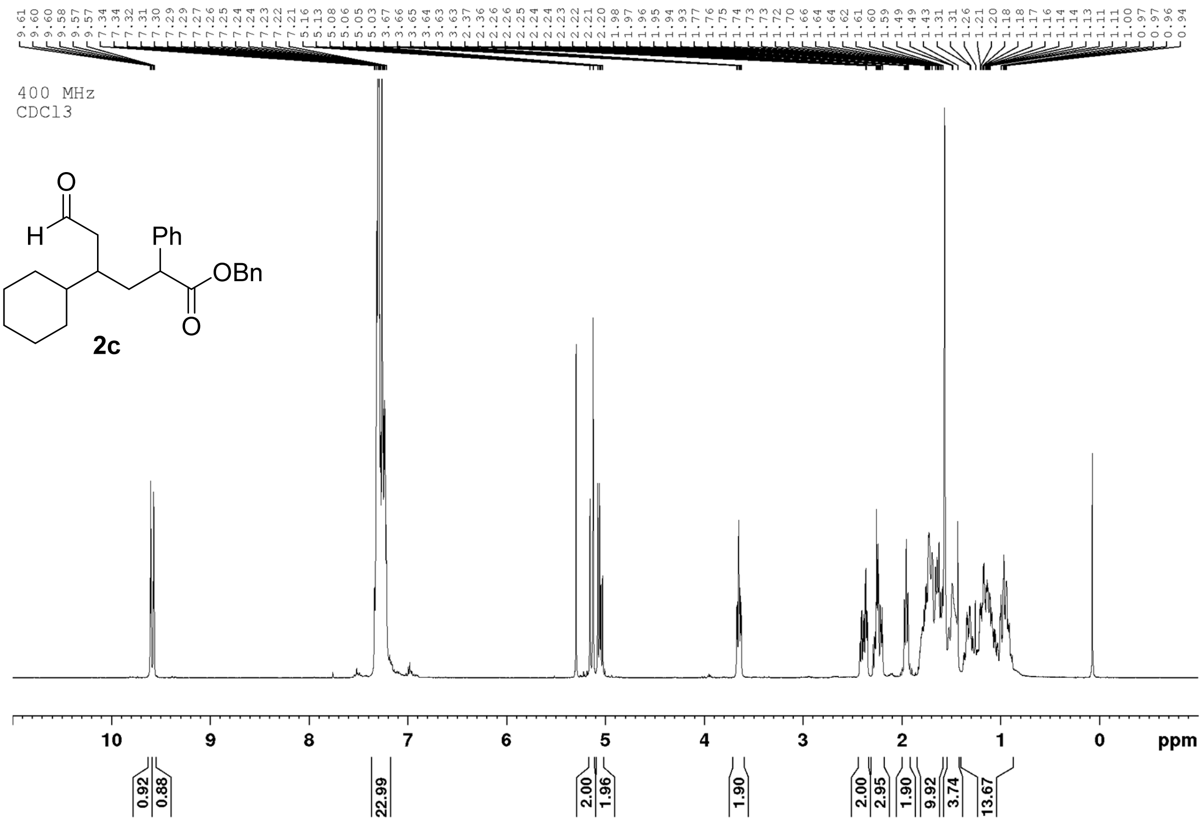
400 MHz
CDCl3
O
H
Ph
OBn
O
2c
ppm

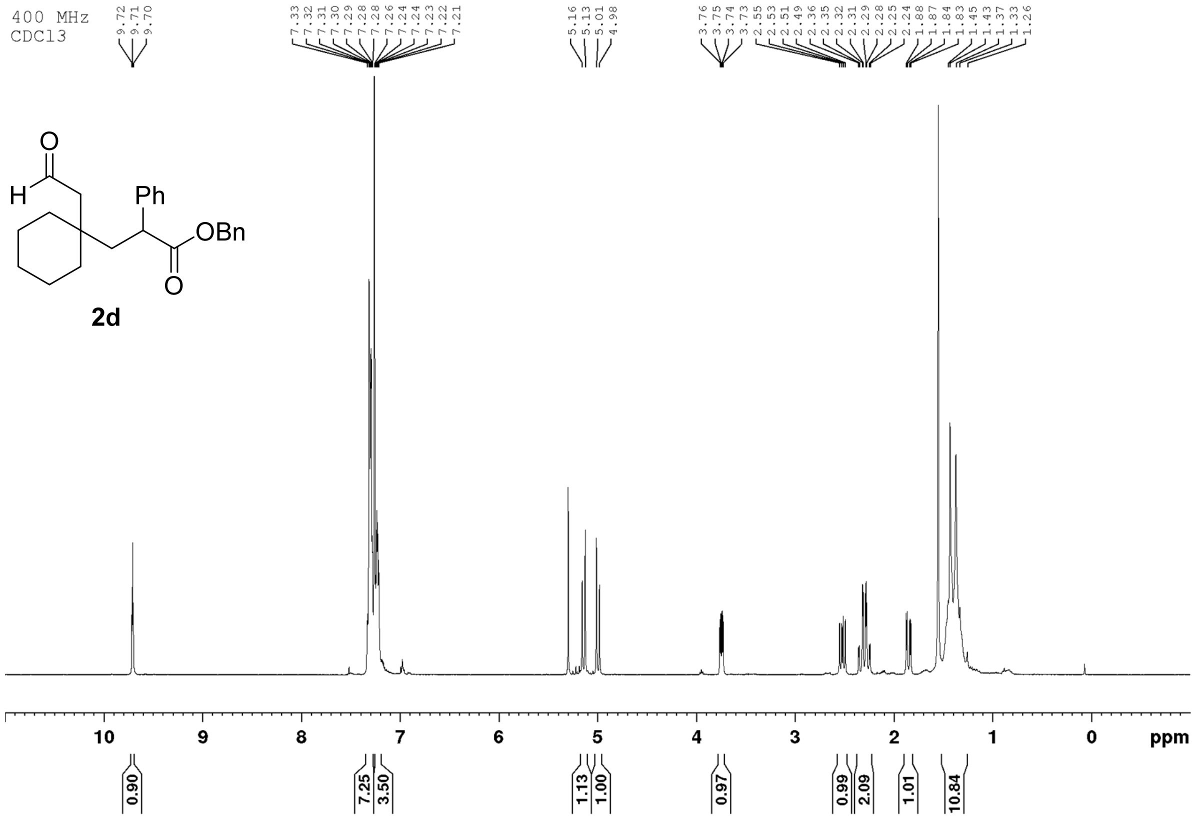
400 MHz
CDCl3
O
H
Ph
OBn
O
2d
ppm

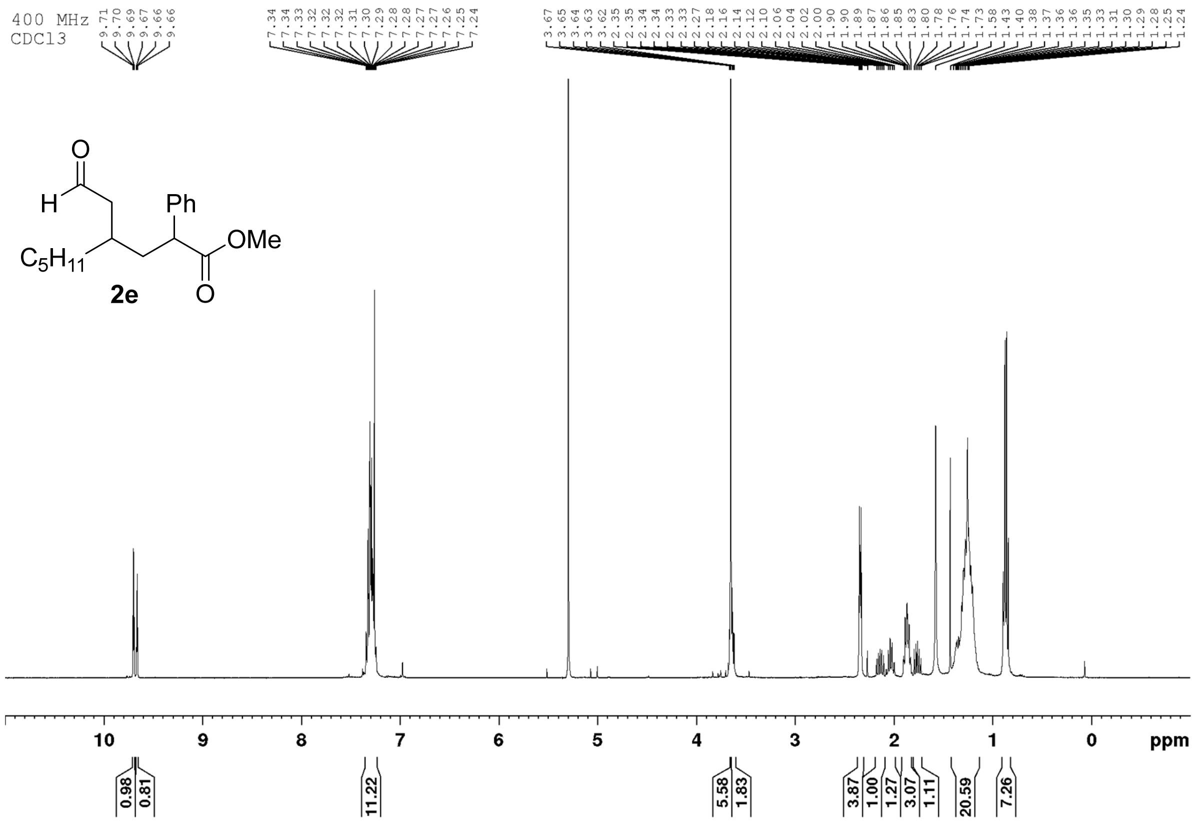
400 MHz
CDCl3
O
H
Ph
OMe
C5H11
O
2e
ppm
0.98
0.81
11.22
5.58
1.83
3.87
1.00
1.27
3.07
1.11
20.59
7.26

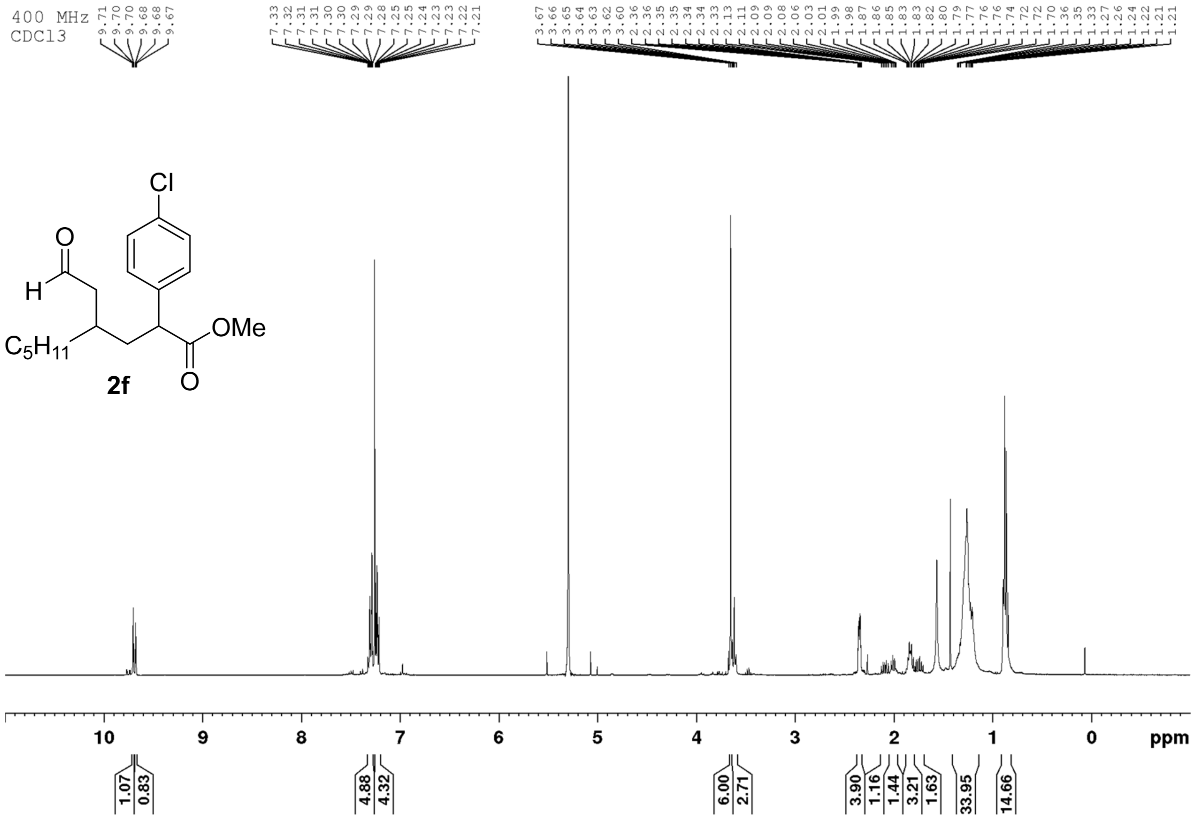
400 MHz
CDCl3
Cl
O
H
OMe
C5H11
O
2f
ppm
1.07
0.83
4.88
4.32
6.00
2.71
3.90
1.16
1.44
3.21
1.63
33.95
14.66

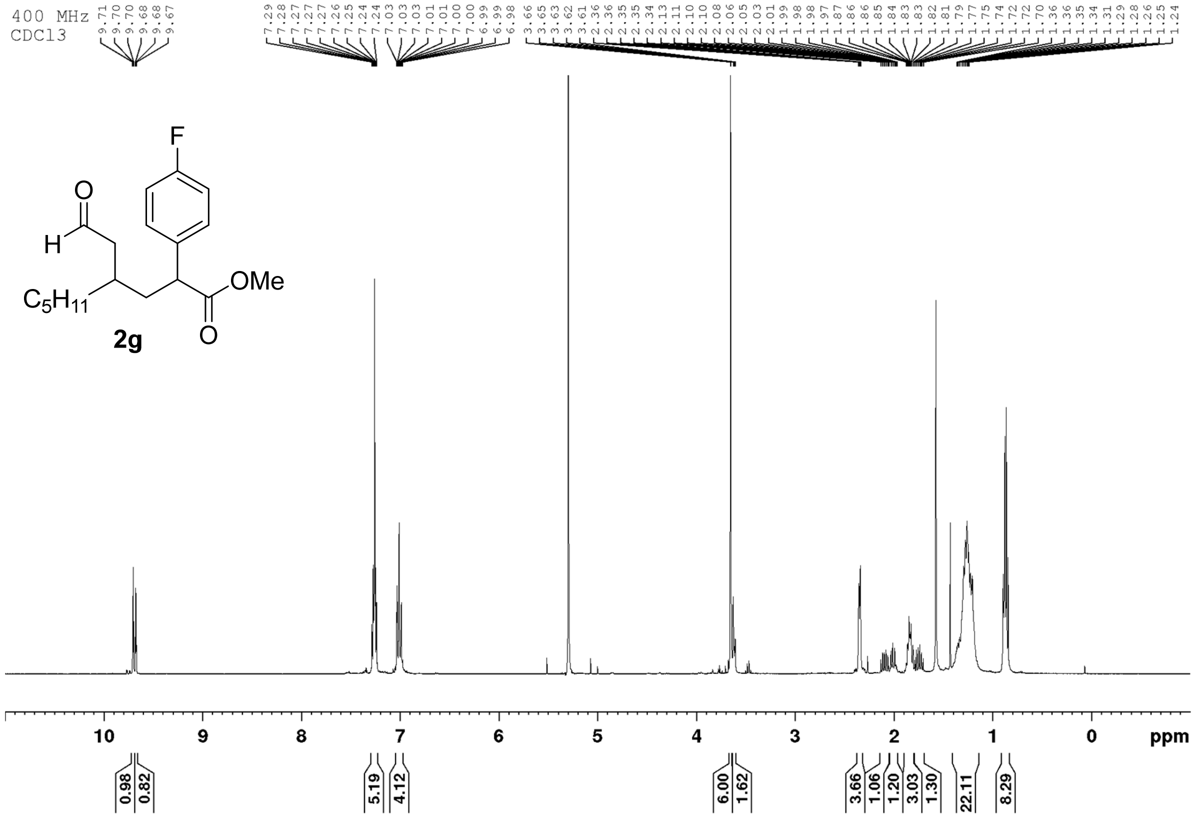
400 MHz
CDCl3
F
O
H
OMe
C5H11
O
2g
10 9 8 7 6 5 4 3 2 1 0 ppm
0.98 0.82 5.19 4.12 6.00 1.62 3.66 1.06 1.20 3.03 1.30 22.11 8.29

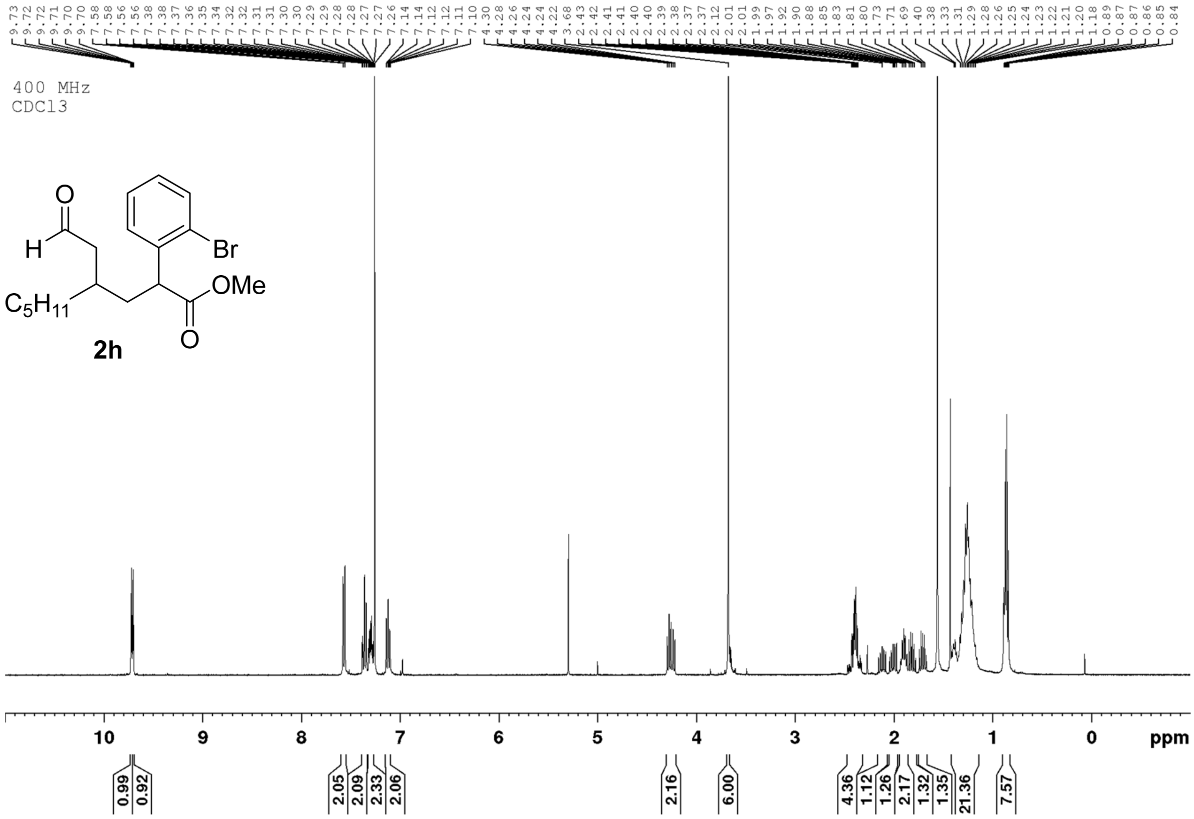
400 MHz
CDCl3
O
H
Br
OMe
C5H11
O
2h
10 9 8 7 6 5 4 3 2 1 0 ppm
0.99 0.92 2.05 2.09 2.33 2.06 2.16 6.00 4.36 1.12 1.26 2.17 1.32 1.35 21.36 7.57

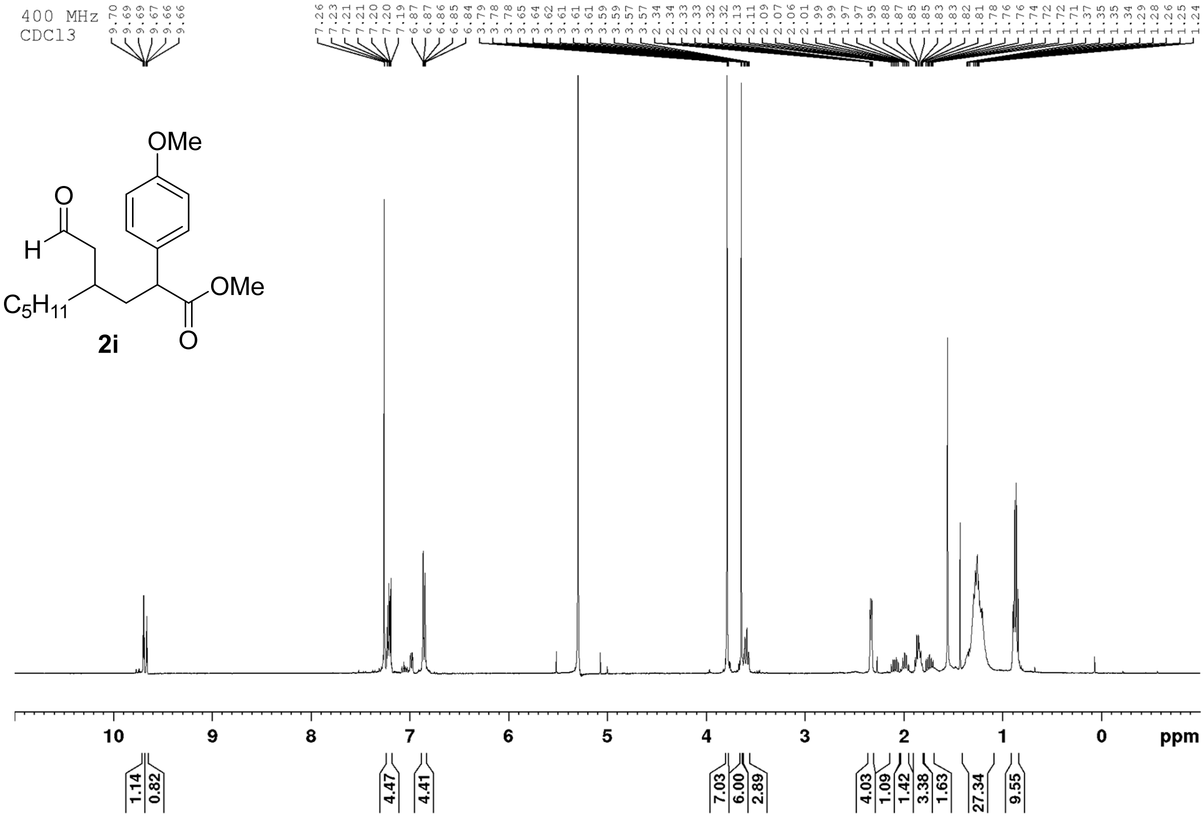

400 MHz
CDCl3
9.70 9.69 9.69 9.67 9.66 9.66
7.26 7.23 7.21 7.20 7.20 7.19 6.87 6.87 6.86 6.85 6.84 3.79 3.78 3.65 3.64 3.62 3.61 3.61 3.61 3.59 3.59 3.57 2.34 2.34 2.33 2.32 2.32 2.13 2.11 2.09 2.07 2.06 2.01 1.99 1.99 1.97 1.97 1.95 1.88 1.87 1.85 1.85 1.83 1.82 1.81 1.78 1.76 1.74 1.72 1.71 1.37 1.35 1.34 1.29 1.28 1.26 1.25 1.24
OMe
O
H
C_5H_{11}
OMe
O
2i
10 9 8 7 6 5 4 3 2 1 0 ppm
1.14 0.82 4.47 4.41 7.03 6.00 2.89 4.03 1.09 1.42 3.38 1.63 27.34 9.55

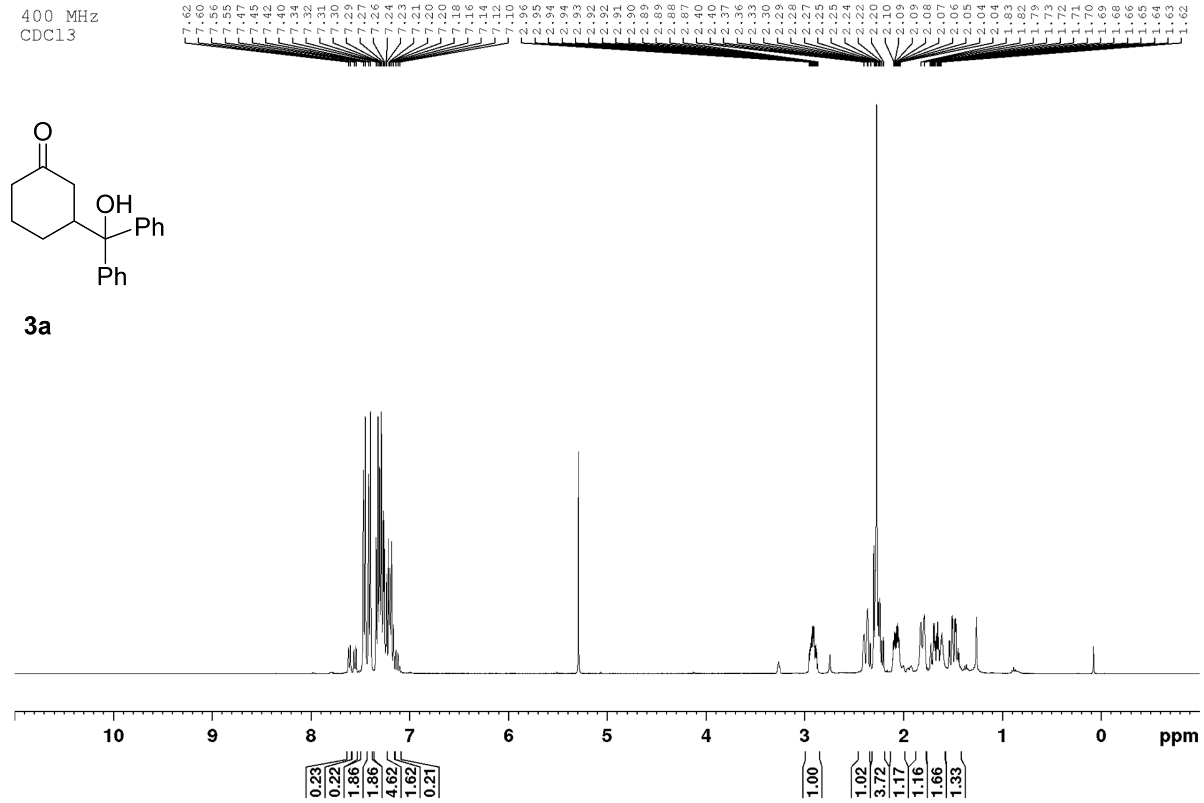

400 MHz
CDCl3
7.62 7.60 7.56 7.55 7.47 7.45 7.42 7.40 7.34 7.32 7.31 7.30 7.29 7.27 7.26 7.24 7.23 7.21 7.20 7.18 7.16 7.14 7.12 7.10 2.96 2.95 2.94 2.93 2.92 2.91 2.90 2.89 2.88 2.87 2.40 2.37 2.36 2.33 2.30 2.29 2.28 2.27 2.25 2.25 2.24 2.22 2.20 2.10 2.09 2.08 2.07 2.06 2.05 2.04 1.83 1.82 1.79 1.73 1.72 1.71 1.70 1.69 1.68 1.65 1.64 1.63 1.62
O
OH
Ph
Ph
3a
10 9 8 7 6 5 4 3 2 1 0 ppm
0.23 0.22 1.86 1.86 4.62 1.62 0.21 1.00 1.02 3.72 1.17 1.16 1.66 1.33

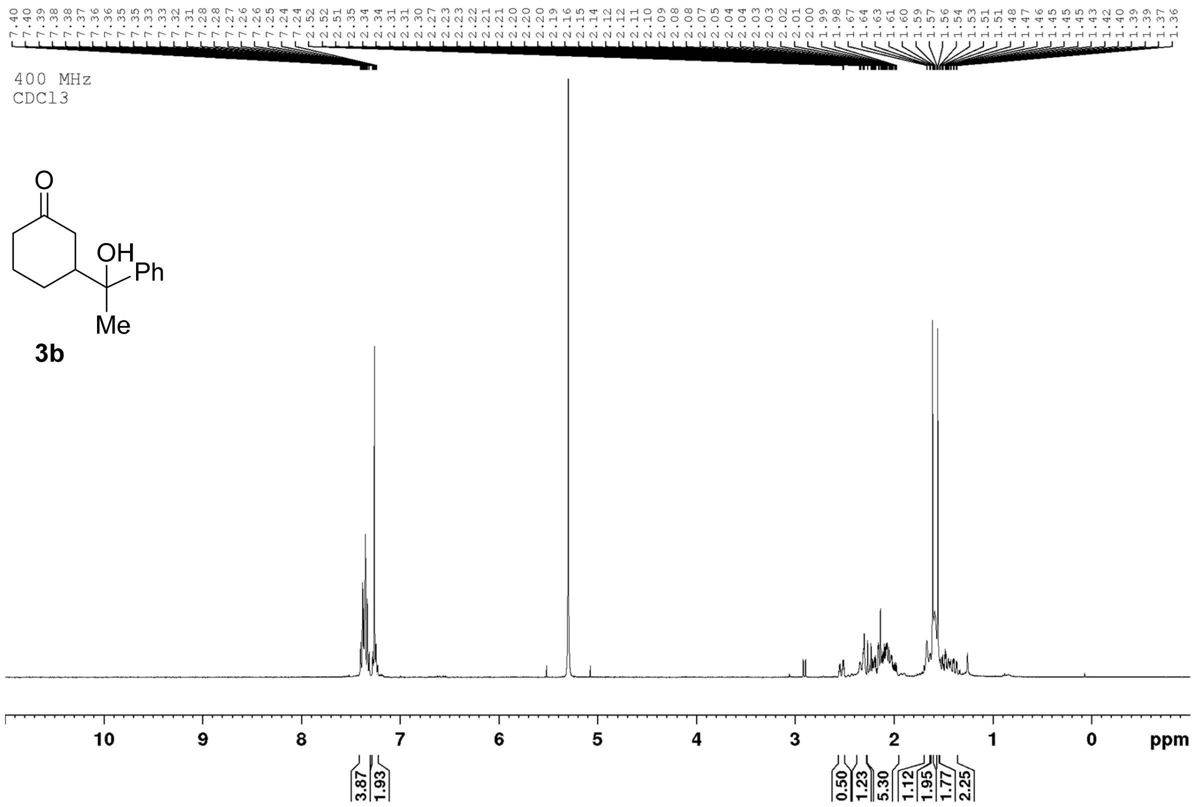
400 MHz
CDCl3
3b
ppm

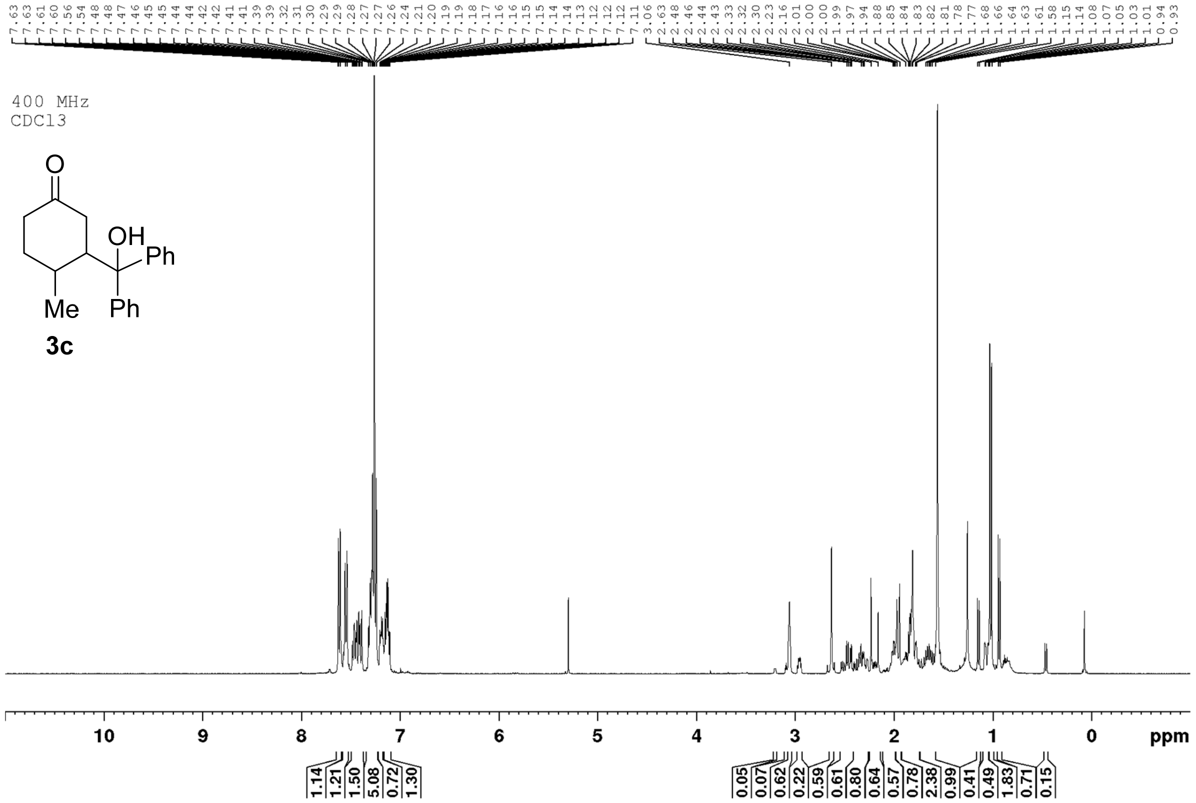
400 MHz
CDCl3
3c
ppm

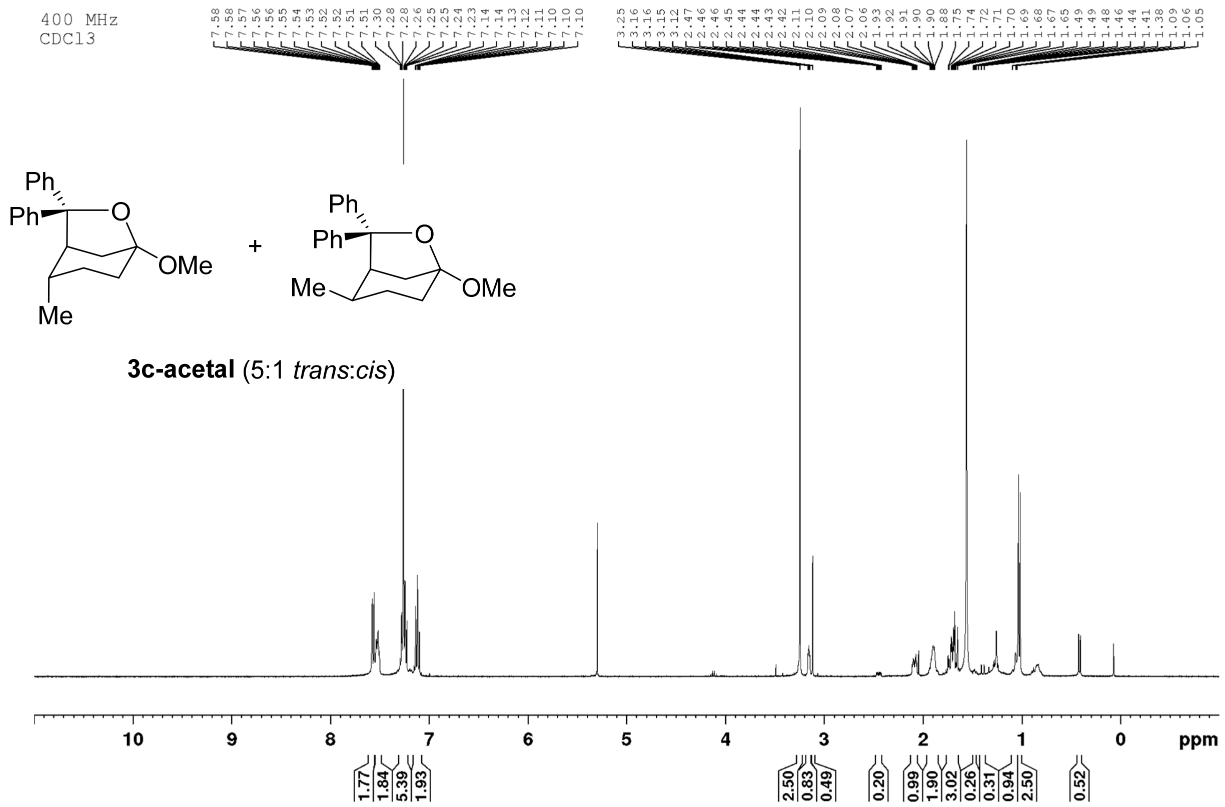
400 MHz
CDCl3
Ph
Ph
O
OMe
Me
+
3c-acetal (5:1 trans:cis)
ppm

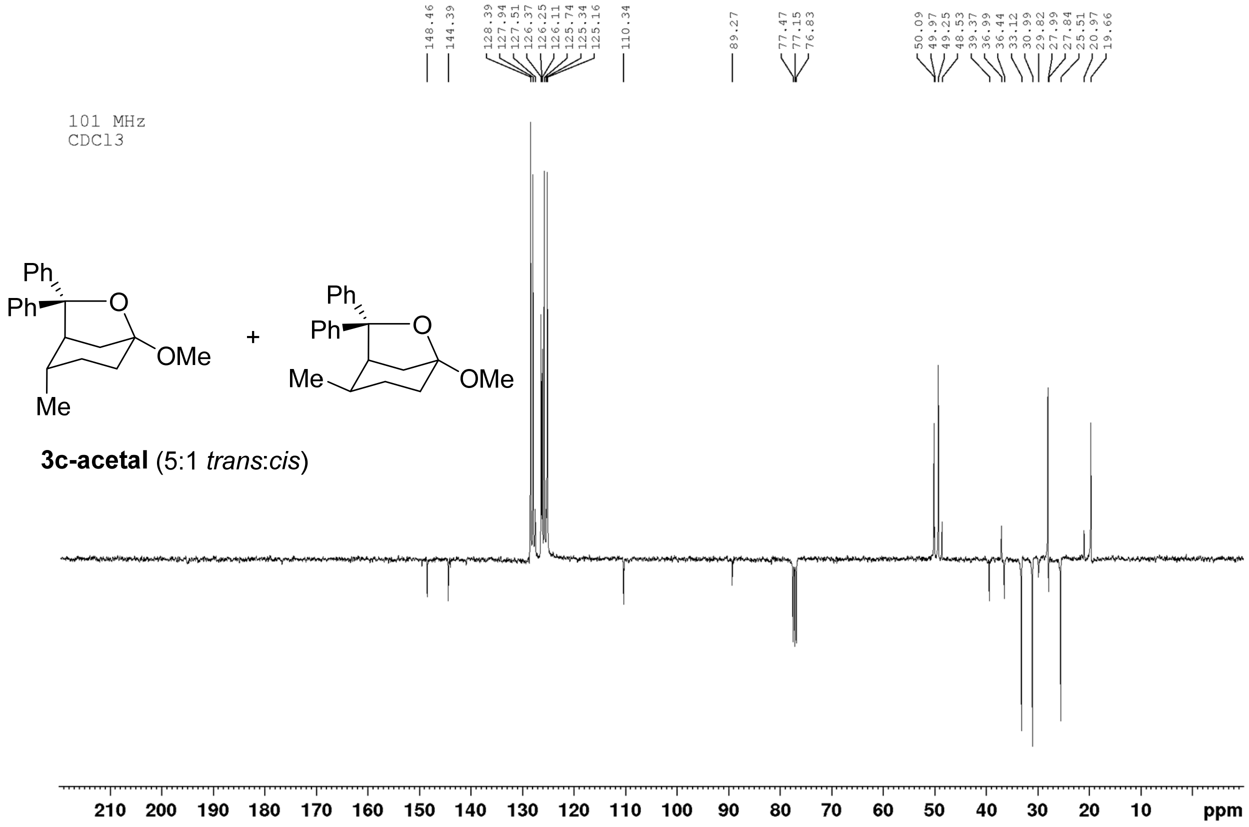
101 MHz
CDCl3
Ph
Ph
O
OMe
Me
+
3c-acetal (5:1 trans:cis)
ppm

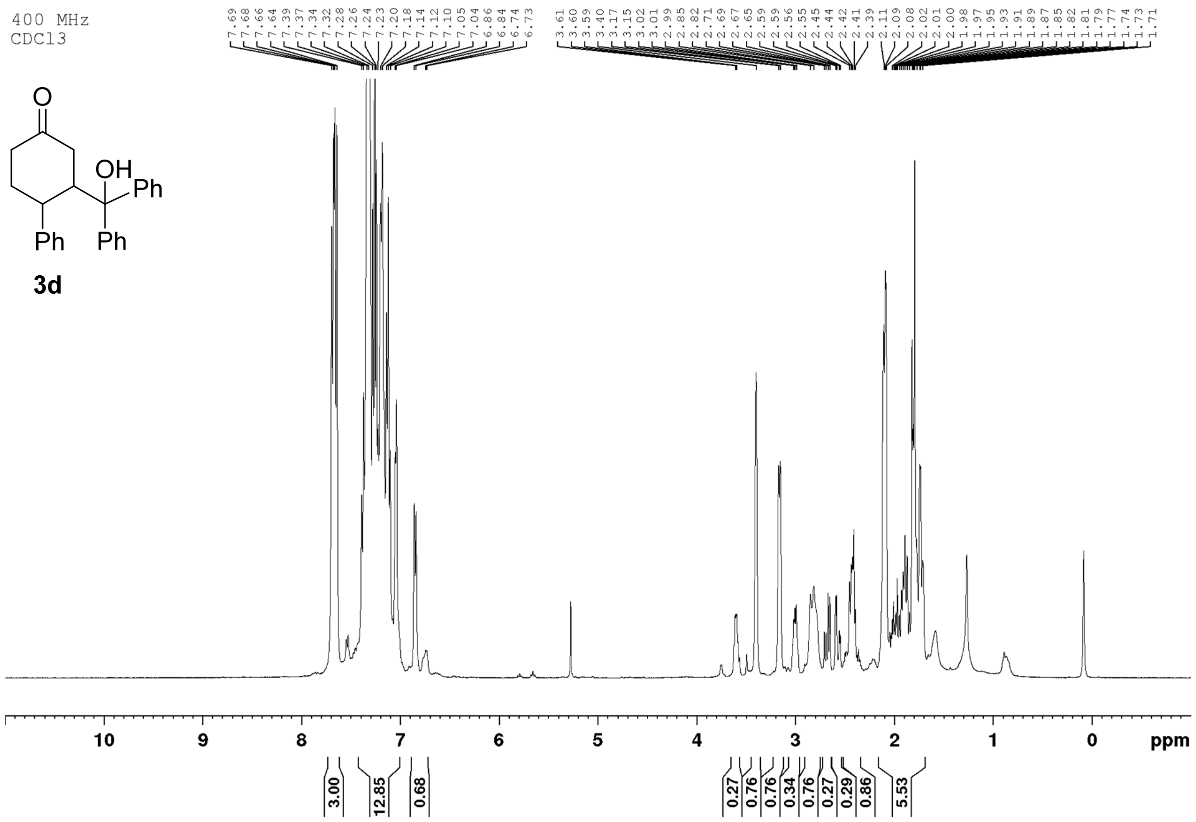
400 MHz
CDCl3
7.69 7.68 7.66 7.64 7.39 7.37 7.34 7.32 7.28 7.26 7.24 7.23 7.20 7.18 7.14 7.12 7.10 7.05 7.04 6.86 6.84 6.74 6.73
3.61 3.60 3.59 3.40 3.17 3.15 3.02 3.01 2.99 2.85 2.82 2.71 2.69 2.67 2.65 2.59 2.59 2.56 2.55 2.45 2.44 2.42 2.41 2.39 2.11 2.09 2.08 2.02 2.01 2.00 1.98 1.97 1.95 1.93 1.91 1.89 1.87 1.85 1.82 1.81 1.79 1.77 1.74 1.73 1.71
O
OH
Ph
Ph
Ph
3d
10 9 8 7 6 5 4 3 2 1 0 ppm
3.00 12.85 0.68
0.27 0.76 0.76 0.34 0.76 0.27 0.29 0.86 5.53

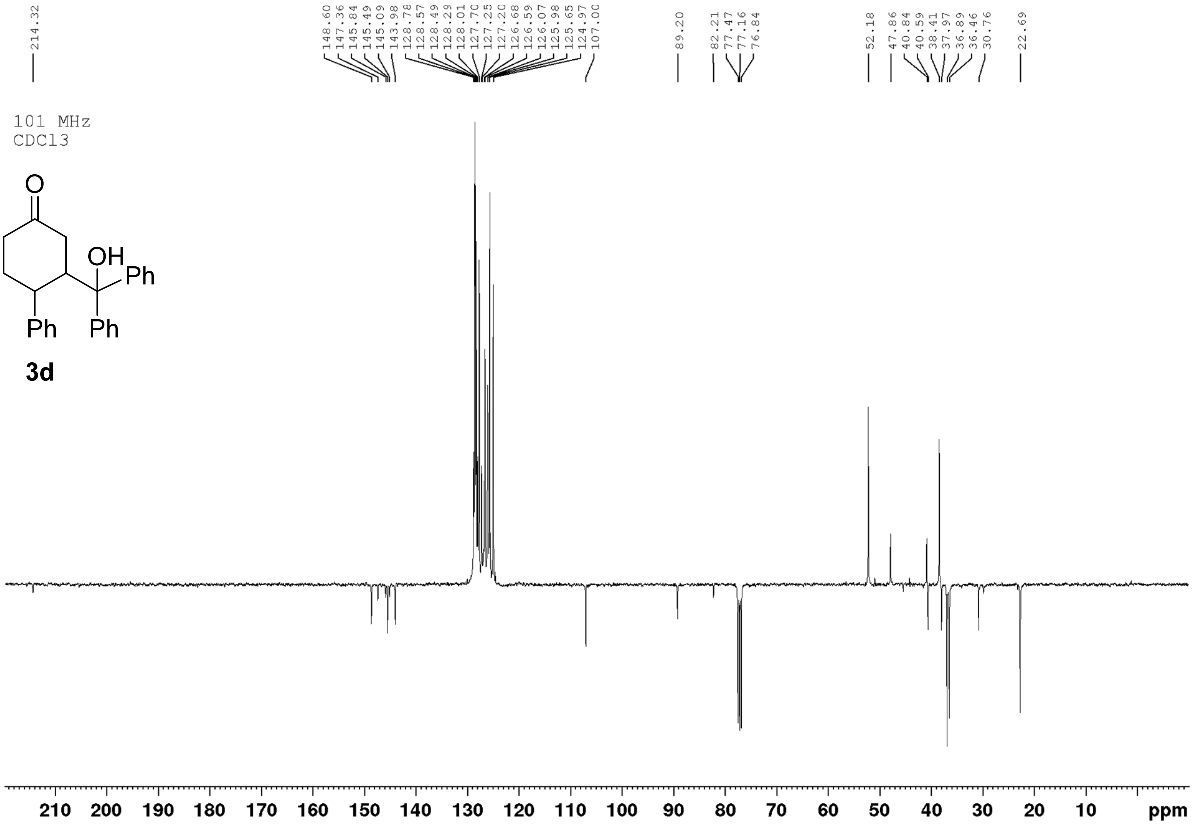
214.32
148.60 147.36 145.84 145.49 145.09 143.98 128.78 128.57 128.49 128.29 128.01 127.70 127.25 127.20 126.68 126.59 126.07 125.98 125.65 124.97 107.00
89.20
82.21 77.47 77.16 76.84
52.18 47.86 40.84 40.59 38.41 37.97 36.89 36.46 30.76
22.69
101 MHz
CDCl3
O
OH
Ph
Ph
Ph
3d
210 200 190 180 170 160 150 140 130 120 110 100 90 80 70 60 50 40 30 20 10 ppm

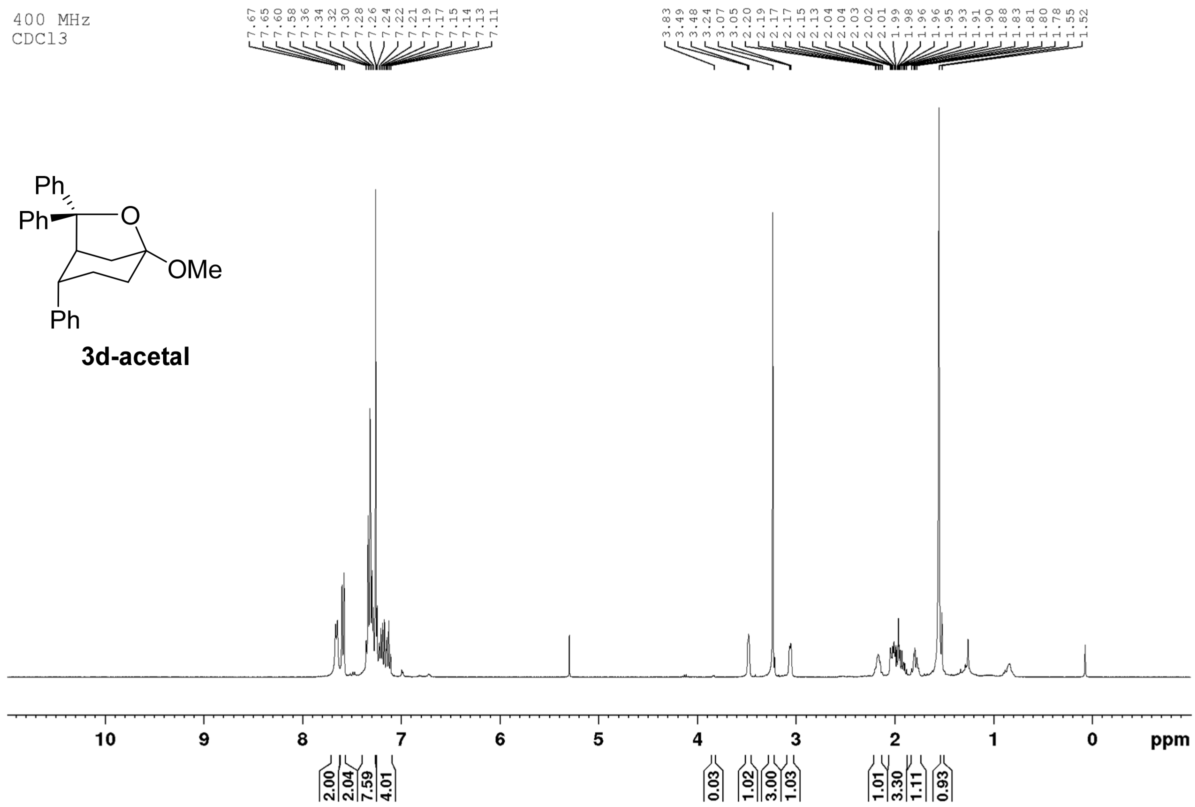
400 MHz
CDCl3
7.67 7.65 7.60 7.58 7.36 7.34 7.32 7.30 7.28 7.26 7.24 7.22 7.21 7.19 7.17 7.15 7.14 7.13 7.11
3.83 3.49 3.48 3.24 3.07 3.05 2.20 2.19 2.17 2.17 2.15 2.13 2.04 2.04 2.03 2.02 2.01 1.99 1.98 1.96 1.95 1.93 1.91 1.90 1.88 1.83 1.81 1.80 1.78 1.55 1.52
Ph
Ph
O
OMe
Ph
3d-acetal
10 9 8 7 6 5 4 3 2 1 0 ppm
2.00 2.04 7.59 4.01 0.03 1.02 3.00 1.03 1.01 3.30 1.11 0.93

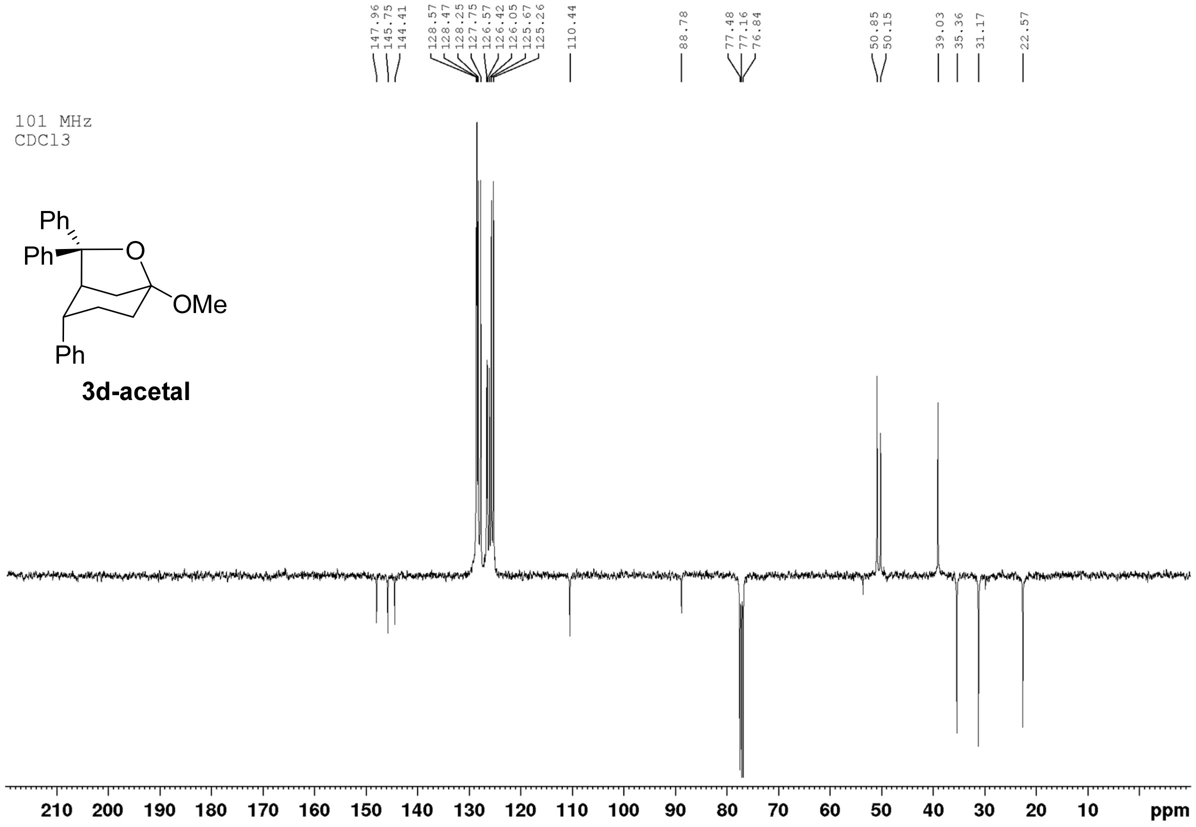
147.96 145.75 144.41
128.57 128.47 128.25 127.75 126.57 126.42 126.05 125.67 125.26
110.44
88.78
77.48 77.16 76.84
50.85 50.15
39.03 35.36 31.17
22.57
101 MHz
CDCl3
Ph
Ph
O
OMe
Ph
3d-acetal
210 200 190 180 170 160 150 140 130 120 110 100 90 80 70 60 50 40 30 20 10 ppm

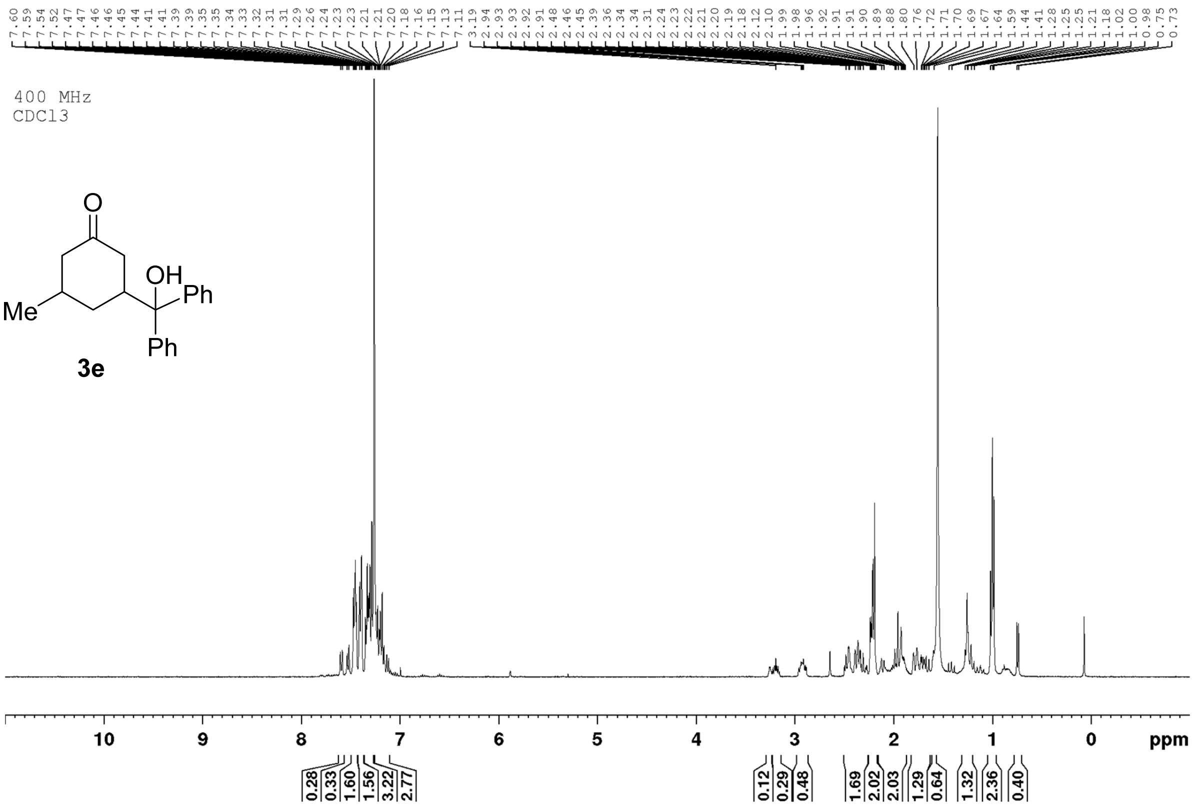
400 MHz
CDCl3
O
OH
Ph
Me
Ph
3e
ppm

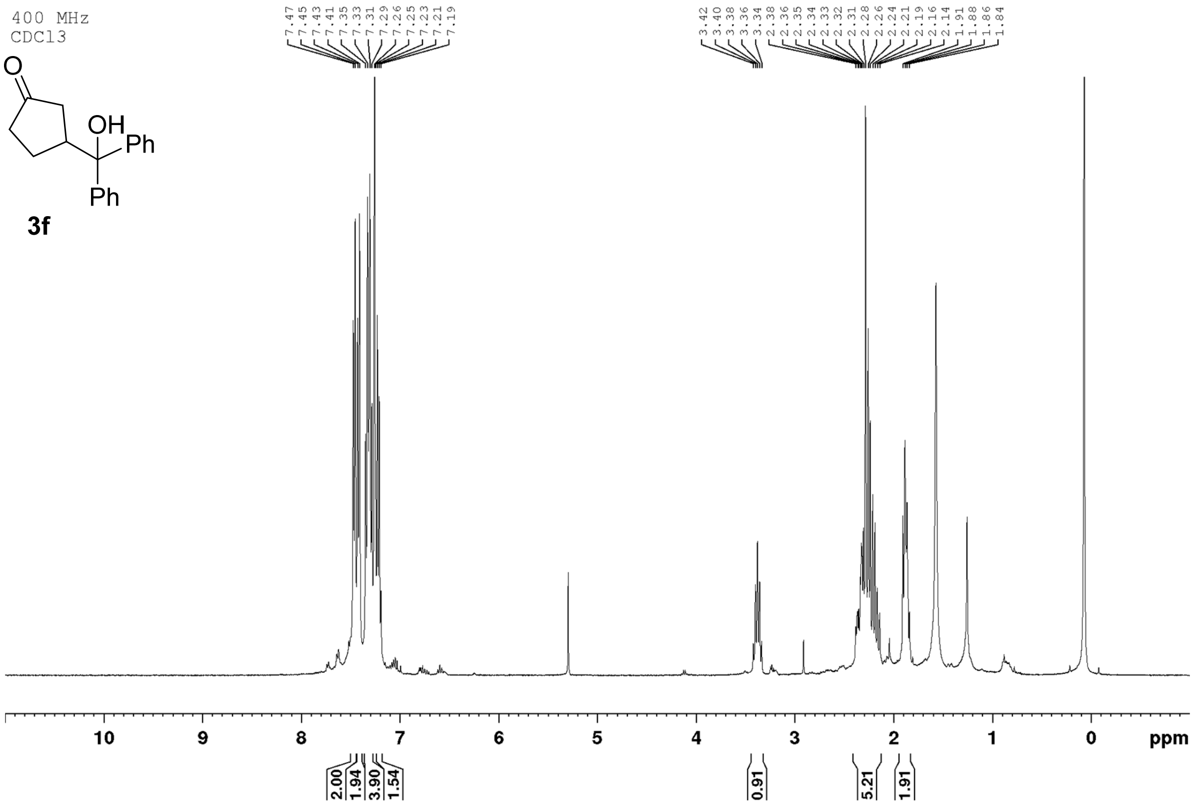
400 MHz
CDCl3
O
OH
Ph
Ph
3f
ppm

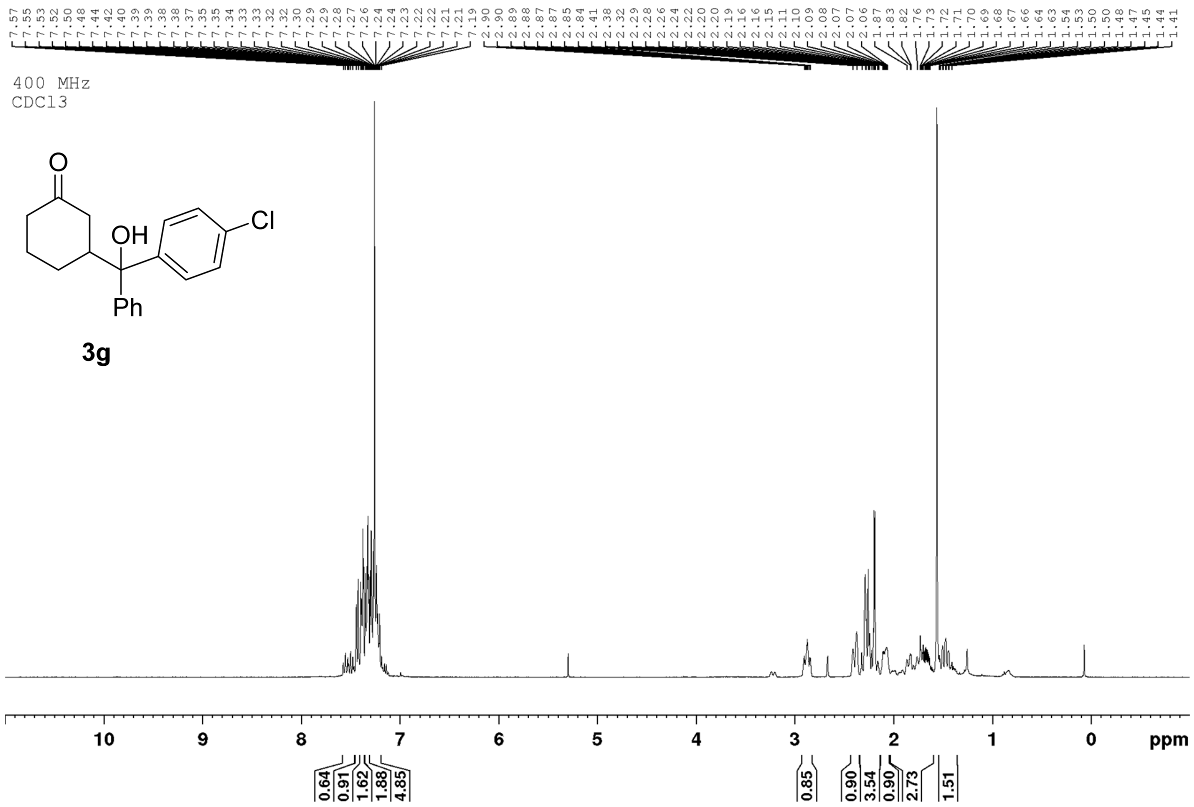
400 MHz
CDCl3
O
OH
Cl
Ph
3g
ppm

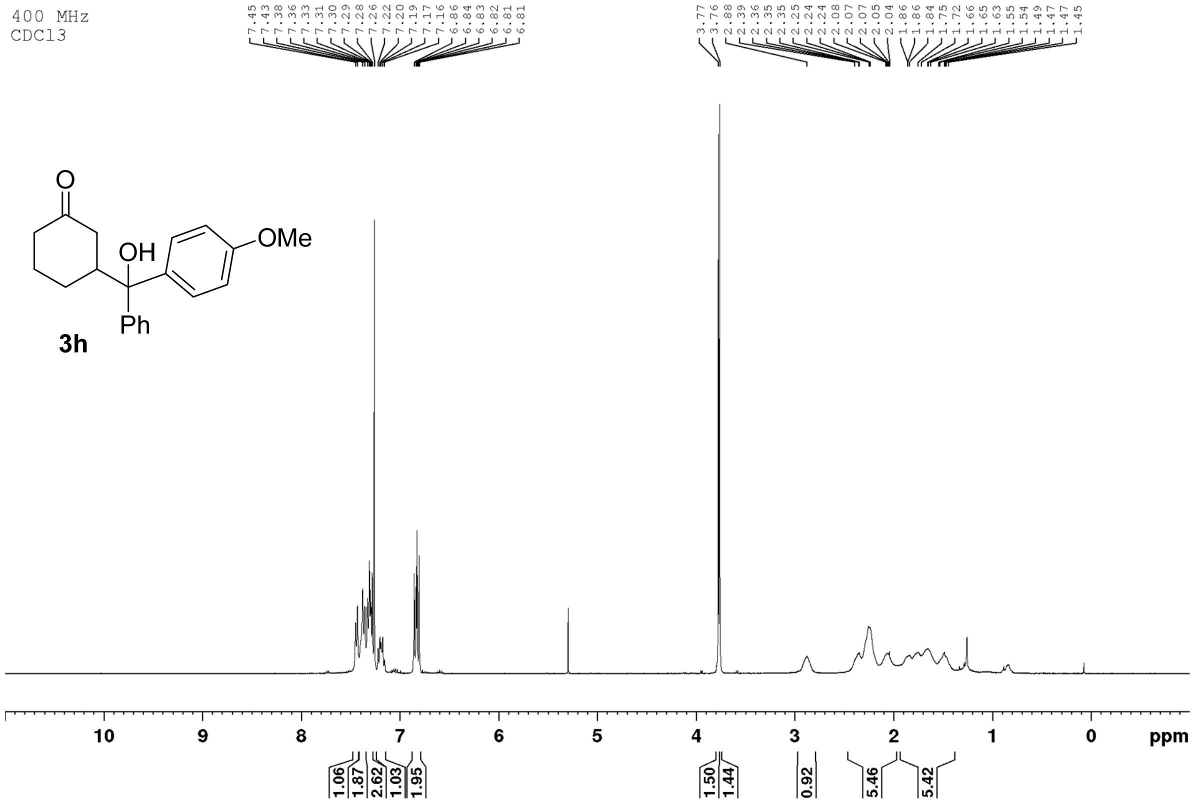
400 MHz
CDCl3
O
OH
OMe
Ph
3h
ppm

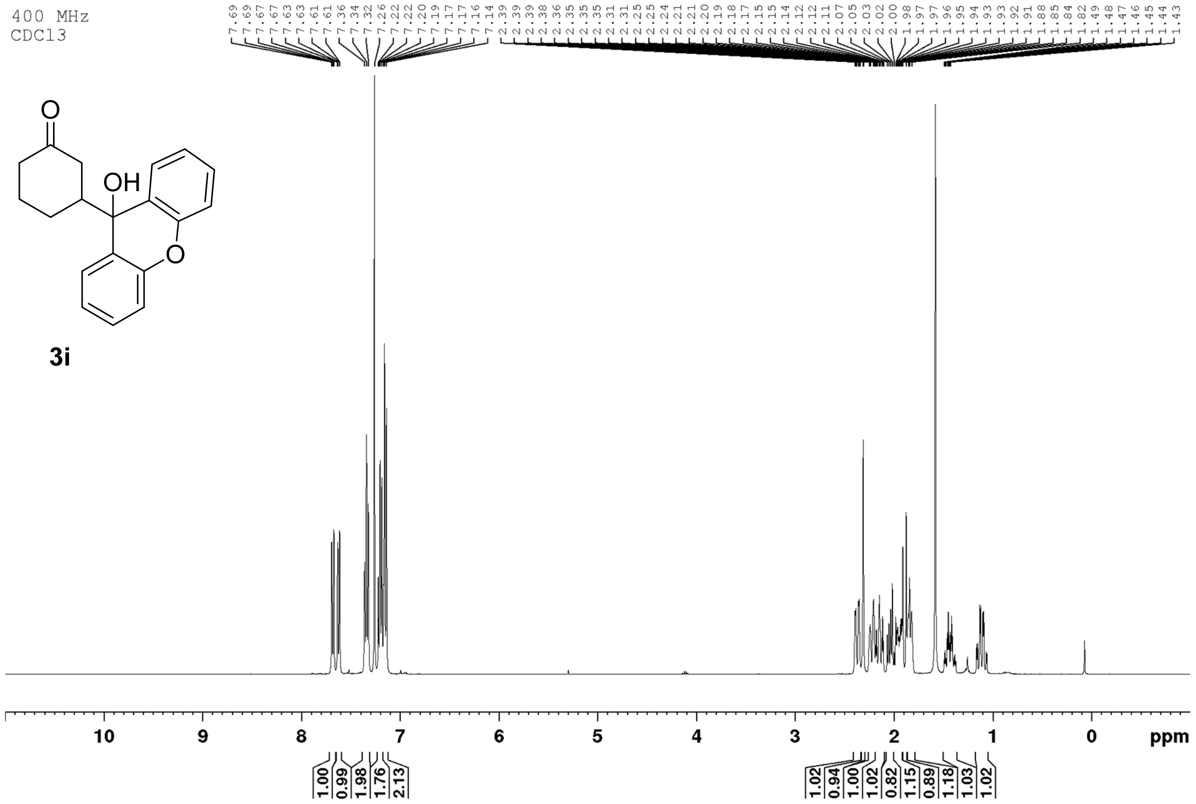
400 MHz
CDCl3
OH
O
3i
ppm

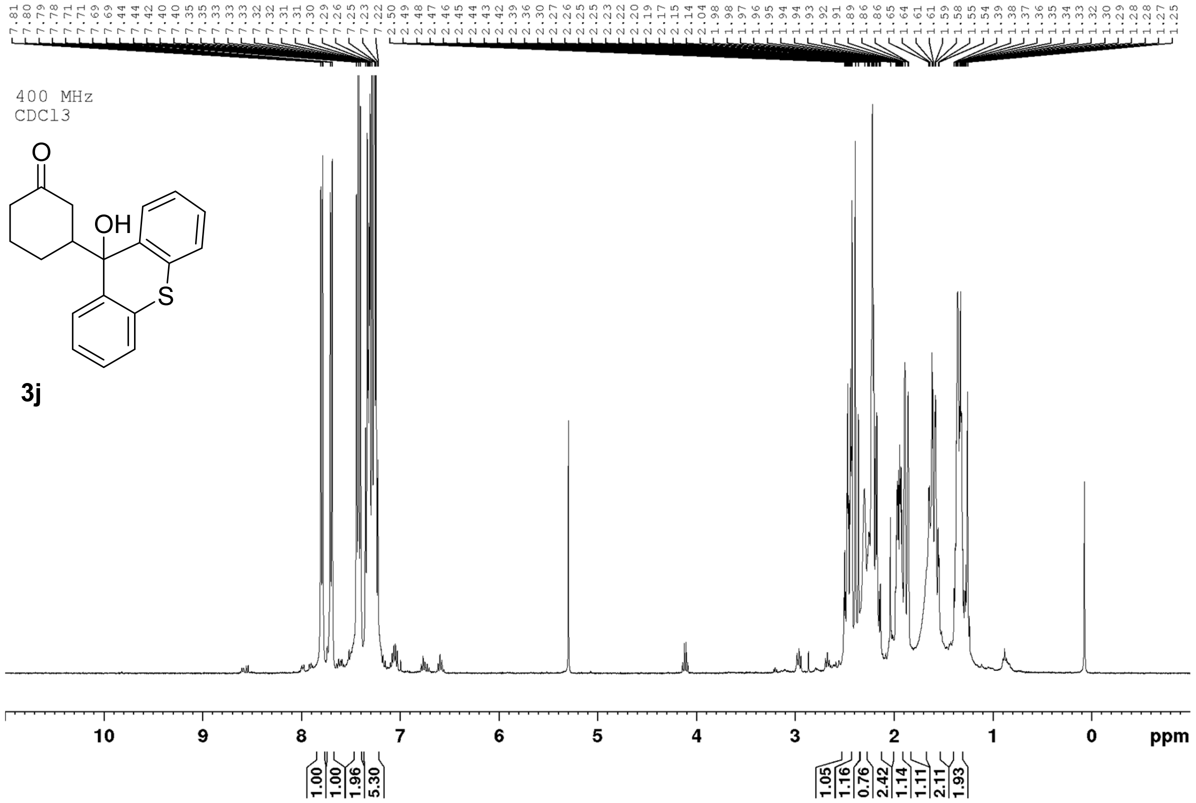
400 MHz
CDCl3
OH
S
3j
ppm

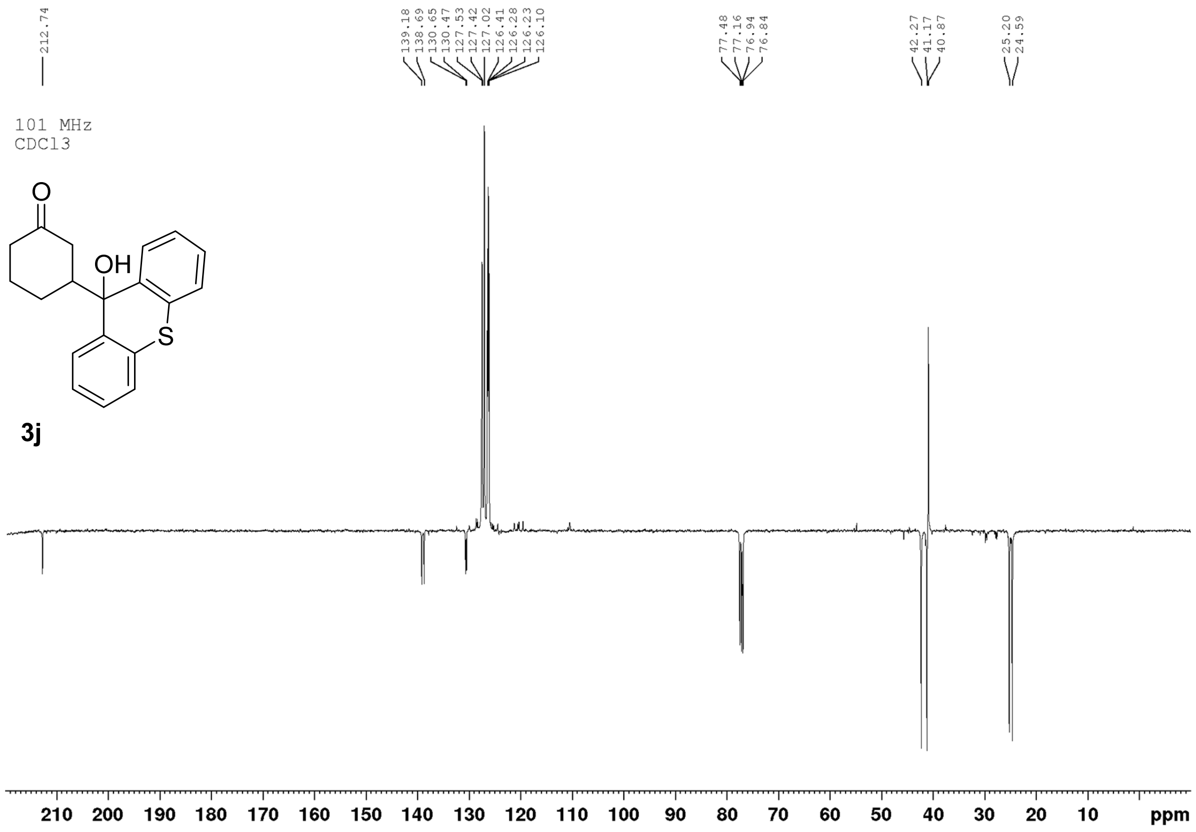

212.74
139.18 138.69 130.65 130.47 127.53 127.42 127.02 126.41 126.28 126.23 126.10
77.48 77.16 76.94 76.84
42.27 41.17 40.87
25.20 24.59
101 MHz
CDCl3
O
OH
S
3j
210 200 190 180 170 160 150 140 130 120 110 100 90 80 70 60 50 40 30 20 10 ppm

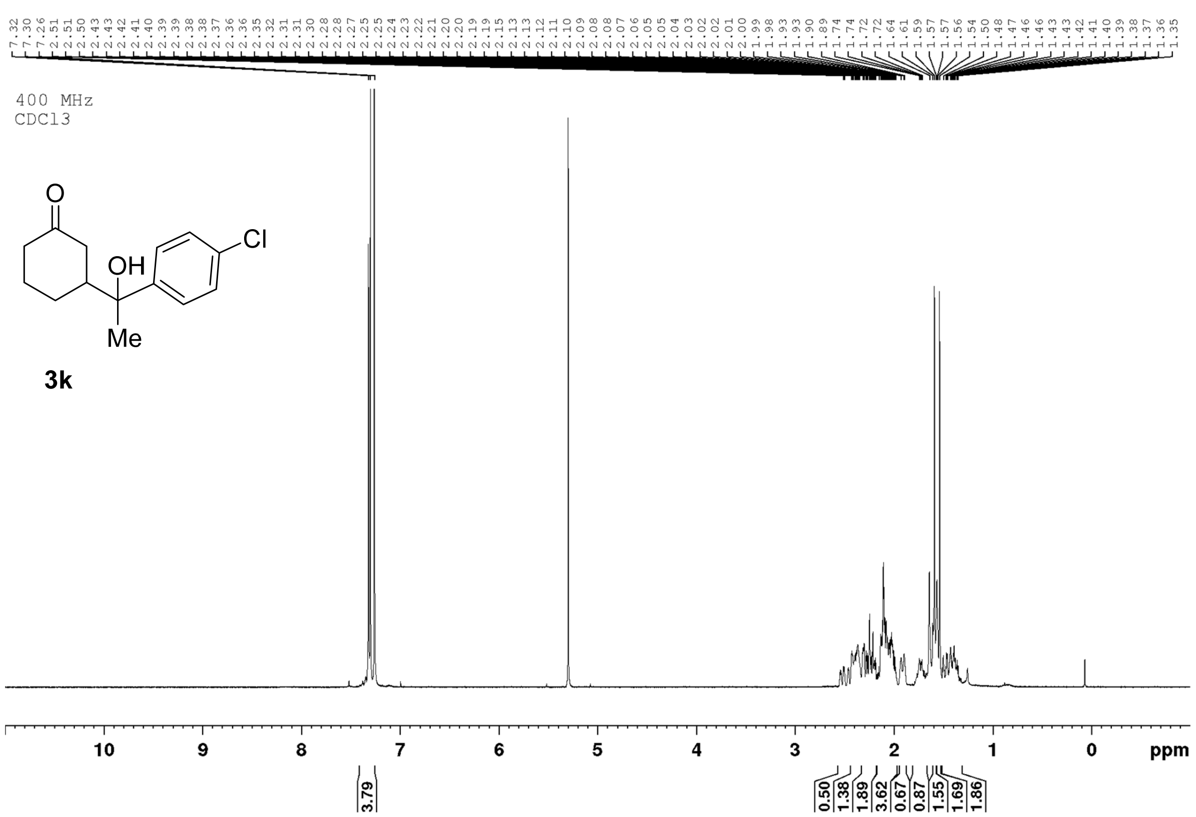

7.32 7.30 7.26 2.51 2.50 2.43 2.42 2.41 2.40 2.39 2.39 2.38 2.37 2.36 2.36 2.35 2.32 2.31 2.30 2.28 2.28 2.27 2.25 2.25 2.24 2.23 2.22 2.21 2.20 2.19 2.19 2.15 2.13 2.13 2.12 2.11 2.10 2.09 2.08 2.08 2.07 2.06 2.05 2.05 2.04 2.03 2.02 2.01 2.00 1.99 1.98 1.93 1.93 1.90 1.89 1.74 1.72 1.64 1.61 1.59 1.57 1.57 1.56 1.54 1.50 1.48 1.47 1.46 1.43 1.43 1.42 1.41 1.40 1.39 1.38 1.37 1.36 1.35
400 MHz
CDCl3
O
OH
Cl
Me
3k
10 9 8 7 6 5 4 3 2 1 0 ppm
3.79
0.50 1.38 1.89 3.62 0.67 0.87 1.55 1.69 1.86

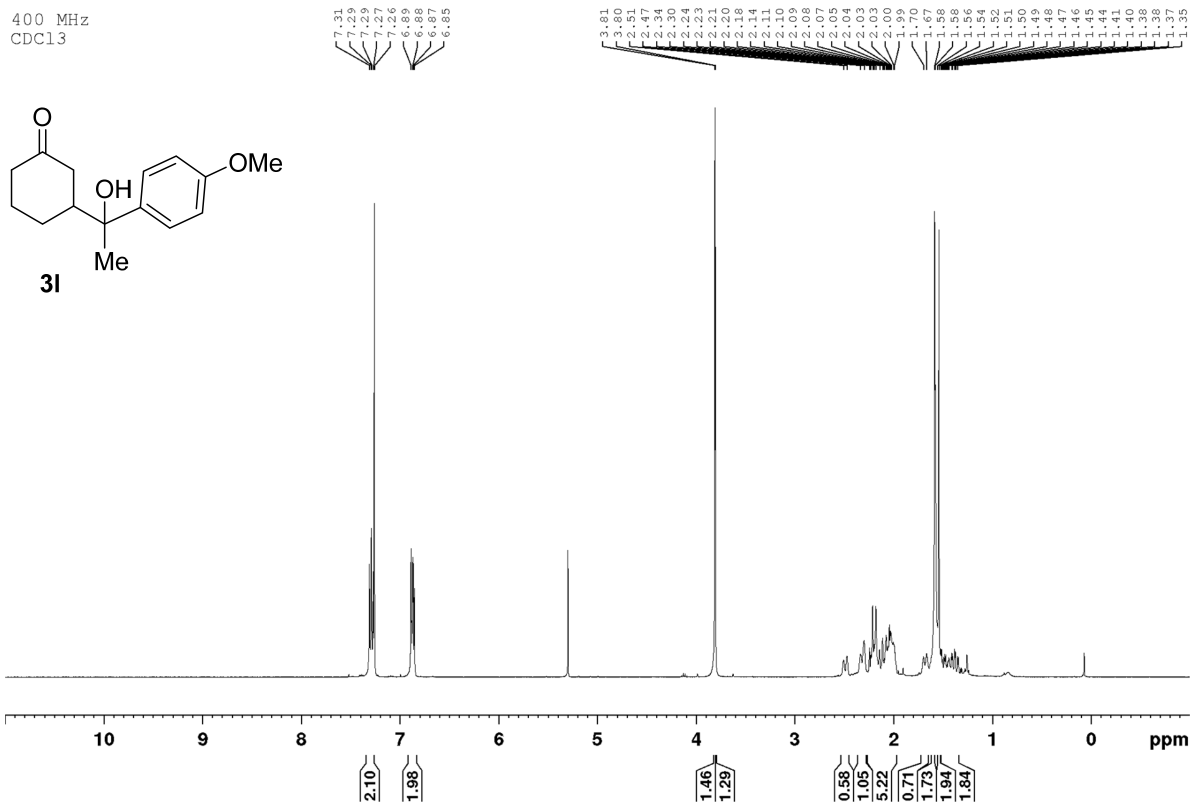
400 MHz
CDCl3
7.31
7.29
7.29
7.27
7.26
6.89
6.88
6.87
6.85
3.81
3.80
2.51
2.47
2.34
2.30
2.24
2.23
2.21
2.20
2.18
2.14
2.11
2.10
2.09
2.08
2.07
2.05
2.04
2.03
2.03
2.00
1.99
1.70
1.67
1.58
1.58
1.56
1.54
1.52
1.51
1.50
1.49
1.48
1.47
1.46
1.45
1.44
1.41
1.40
1.38
1.37
1.35
O
OH
OMe
Me
3l
10
9
8
7
6
5
4
3
2
1
0
ppm
2.10
1.98
1.46
1.29
0.58
1.05
5.22
0.71
1.73
1.94
1.84

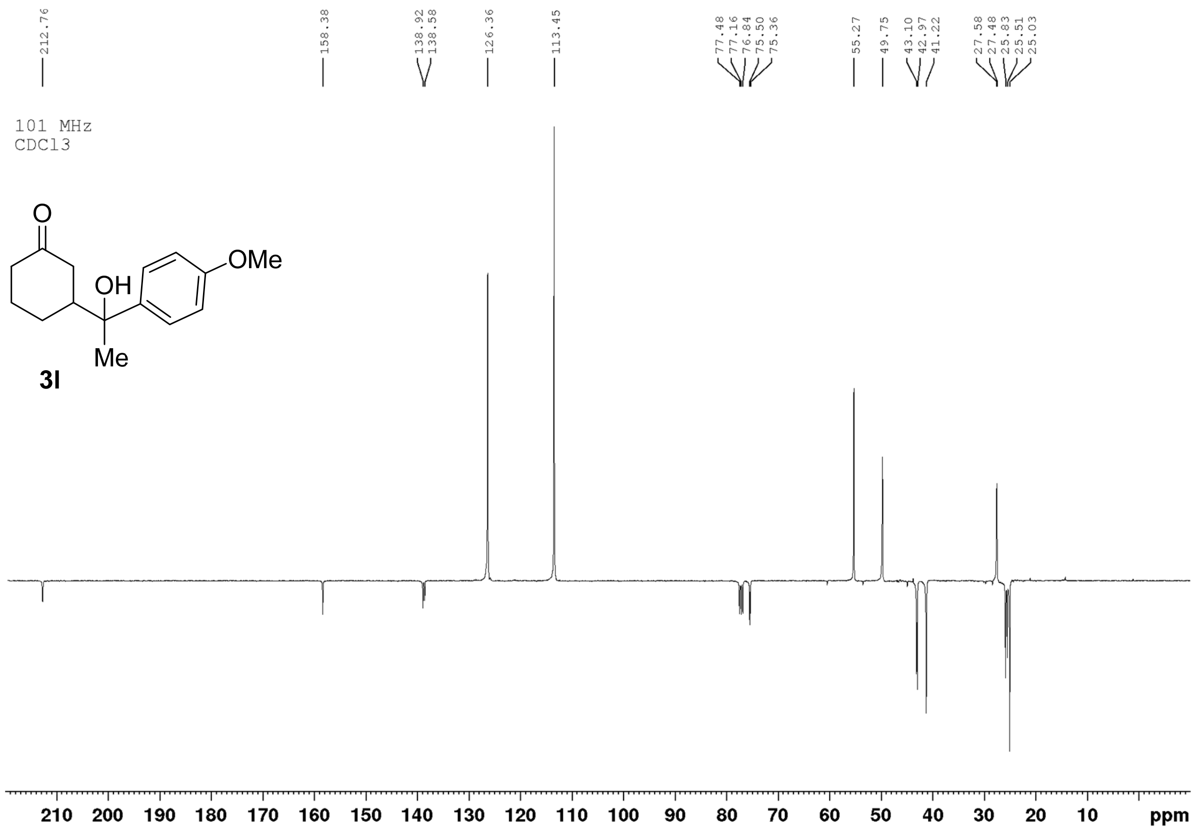
212.76
158.38
138.92
138.58
126.36
113.45
77.48
77.16
76.84
75.50
75.36
55.27
49.75
43.10
42.97
41.22
27.58
27.48
25.83
25.51
25.03
101 MHz
CDCl3
O
OH
OMe
Me
3l
210
200
190
180
170
160
150
140
130
120
110
100
90
80
70
60
50
40
30
20
10
ppm

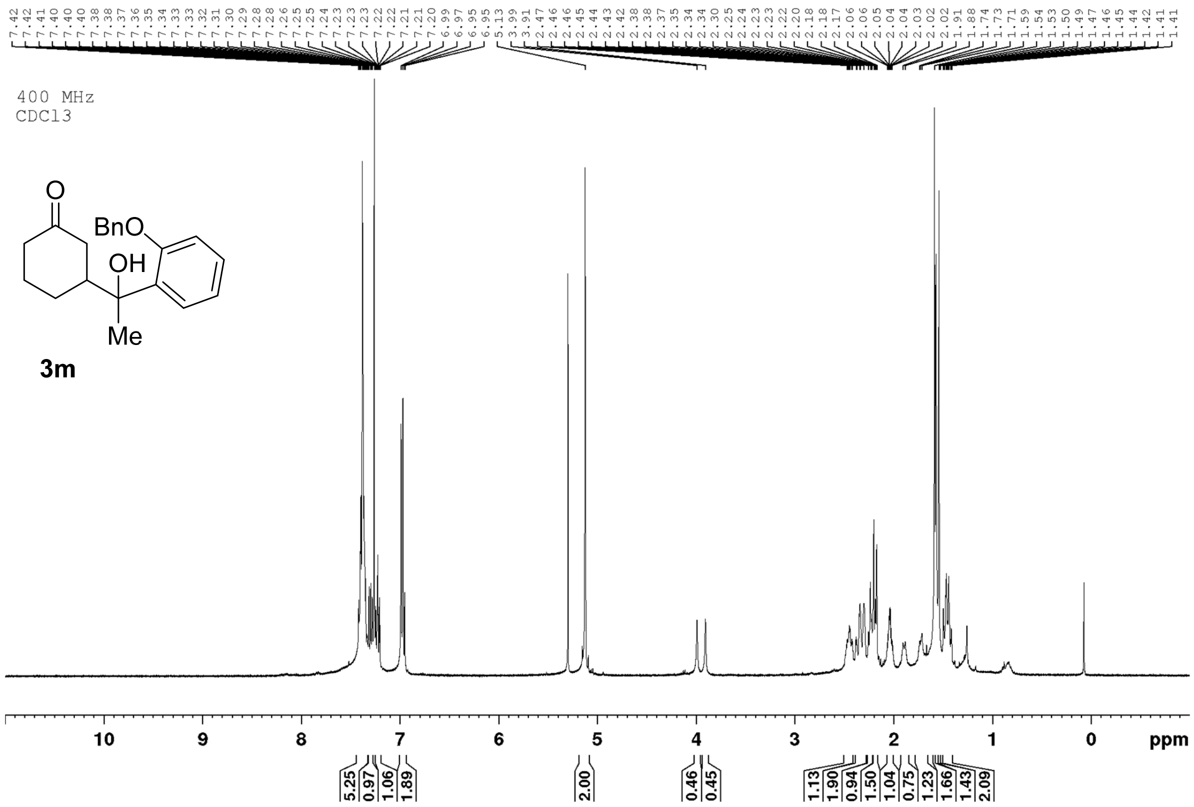
7.42 7.42 7.41 7.40 7.40 7.38 7.38 7.37 7.36 7.35 7.34 7.33 7.33 7.32 7.31 7.30 7.29 7.28 7.26 7.25 7.24 7.23 7.23 7.22 7.22 7.21 7.21 7.20 6.99 6.97 6.95 6.95 5.13 3.99 3.91 2.47 2.46 2.45 2.44 2.43 2.42 2.38 2.38 2.37 2.35 2.34 2.30 2.25 2.24 2.23 2.23 2.22 2.20 2.18 2.18 2.17 2.06 2.06 2.05 2.04 2.04 2.03 2.02 2.02 1.91 1.88 1.74 1.73 1.71 1.59 1.54 1.53 1.50 1.49 1.47 1.46 1.45 1.44 1.42 1.41 1.41
400 MHz
CDCl3
O
BnO
OH
Me
3m
10 9 8 7 6 5 4 3 2 1 0 ppm
5.25 0.97 1.06 1.89 2.00 0.46 0.45 1.13 1.90 0.94 1.50 1.04 0.75 1.23 1.66 1.43 2.09

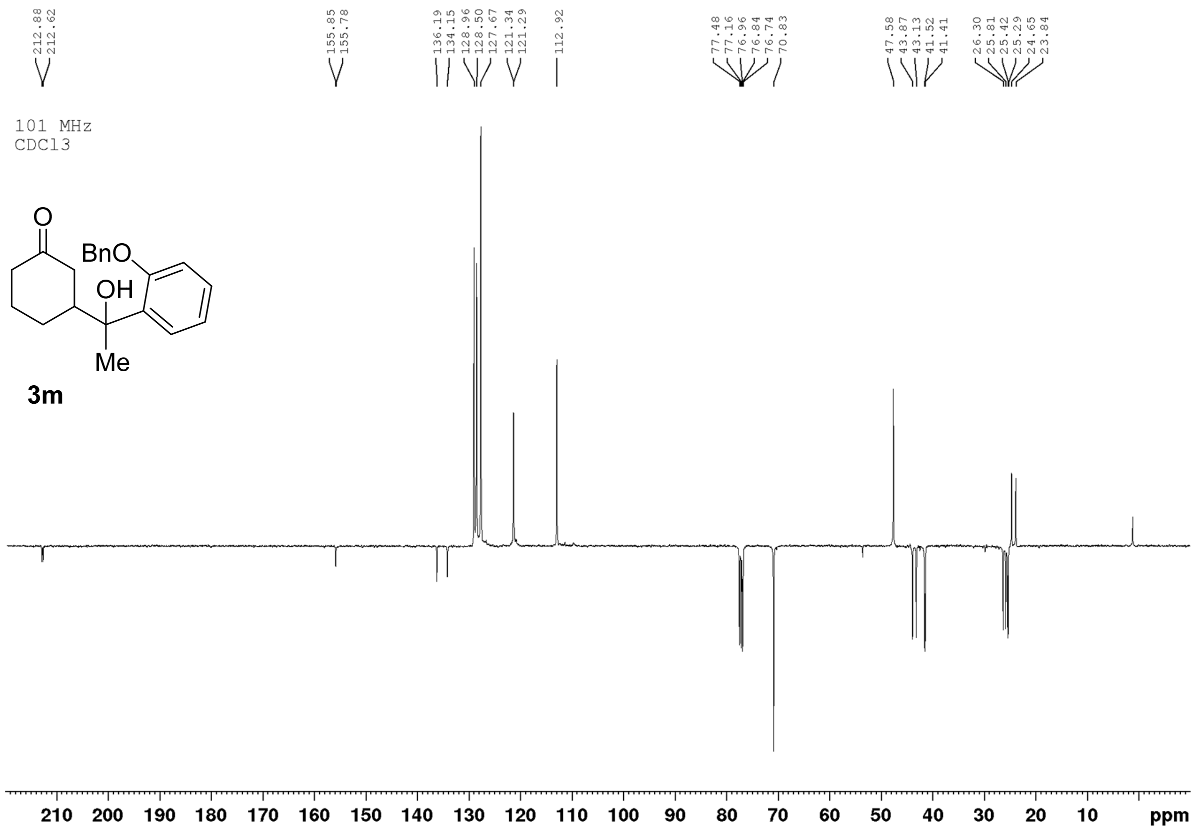
212.88 212.62 155.85 155.78 136.19 134.15 128.96 128.50 127.67 121.34 121.29 112.92 77.48 77.16 76.96 76.84 76.74 70.83 47.58 43.87 43.13 41.52 41.41 26.30 25.81 25.42 25.29 24.65 23.84
101 MHz
CDCl3
O
BnO
OH
Me
3m
210 200 190 180 170 160 150 140 130 120 110 100 90 80 70 60 50 40 30 20 10 ppm

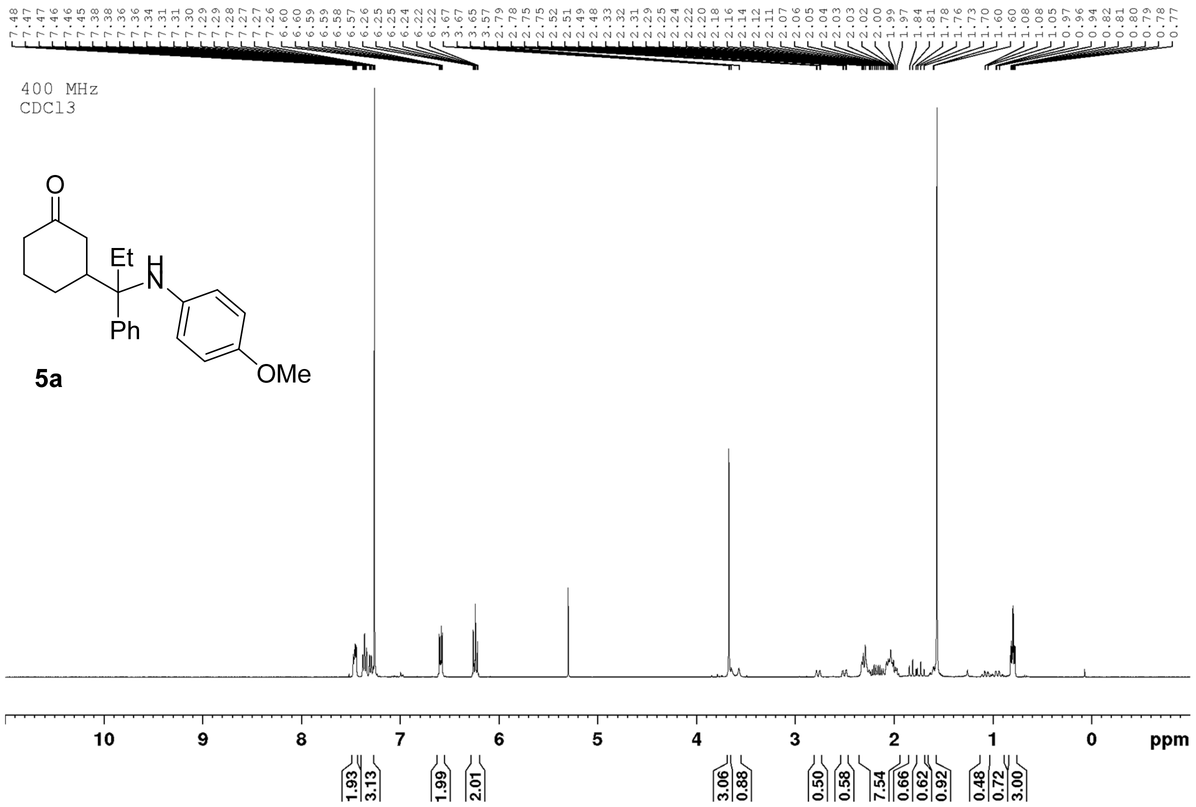
7.48 7.47 7.46 7.46 7.45 7.38 7.38 7.36 7.36 7.34 7.31 7.30 7.29 7.29 7.28 7.27 7.26 6.60 6.60 6.59 6.59 6.58 6.57 6.26 6.25 6.25 6.24 6.22 6.22 3.67 3.65 3.57 2.79 2.78 2.75 2.75 2.52 2.51 2.49 2.48 2.33 2.32 2.31 2.29 2.25 2.24 2.22 2.20 2.18 2.16 2.14 2.12 2.11 2.07 2.06 2.05 2.04 2.03 2.03 2.02 2.00 1.99 1.97 1.84 1.81 1.78 1.76 1.73 1.70 1.60 1.08 1.08 1.05 0.97 0.96 0.94 0.82 0.81 0.80 0.79 0.78 0.77
400 MHz
CDCl3
O
Et
H
N
Ph
OMe
5a
10 9 8 7 6 5 4 3 2 1 0 ppm
1.93 3.13 1.99 2.01 3.06 0.88 0.50 0.58 7.54 0.66 0.62 0.92 0.48 0.72 3.00

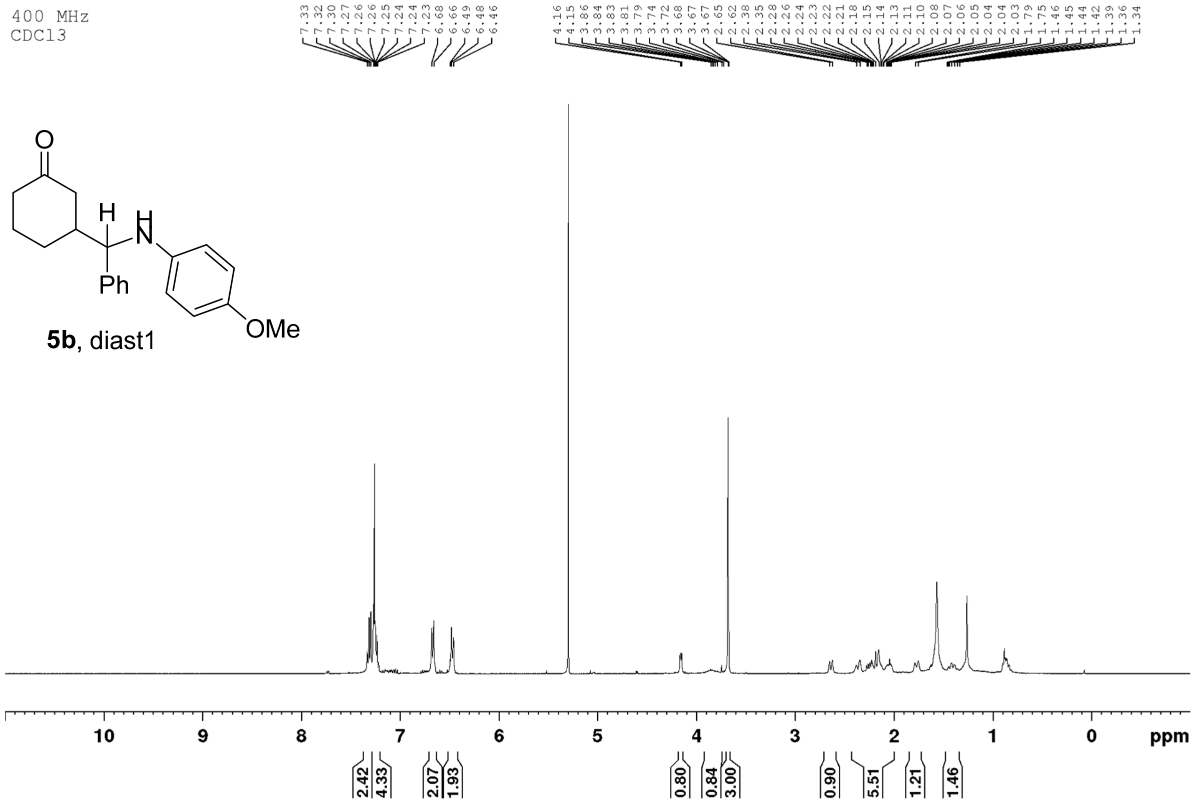
400 MHz
CDCl3
7.33 7.32 7.30 7.27 7.26 7.25 7.24 7.24 7.23 6.68 6.66 6.49 6.48 6.46
4.16 4.15 3.86 3.84 3.83 3.81 3.79 3.74 3.72 3.68 3.67 2.65 2.62 2.38 2.35 2.28 2.26 2.24 2.23 2.22 2.21 2.18 2.15 2.14 2.13 2.11 2.10 2.08 2.07 2.06 2.05 2.04 2.03 1.79 1.75 1.46 1.45 1.44 1.42 1.39 1.36 1.34
O
H
H
N
Ph
OMe
5b, diast1
10 9 8 7 6 5 4 3 2 1 0 ppm
2.42 4.33 2.07 1.93 0.80 0.84 3.00 0.90 5.51 1.21 1.46

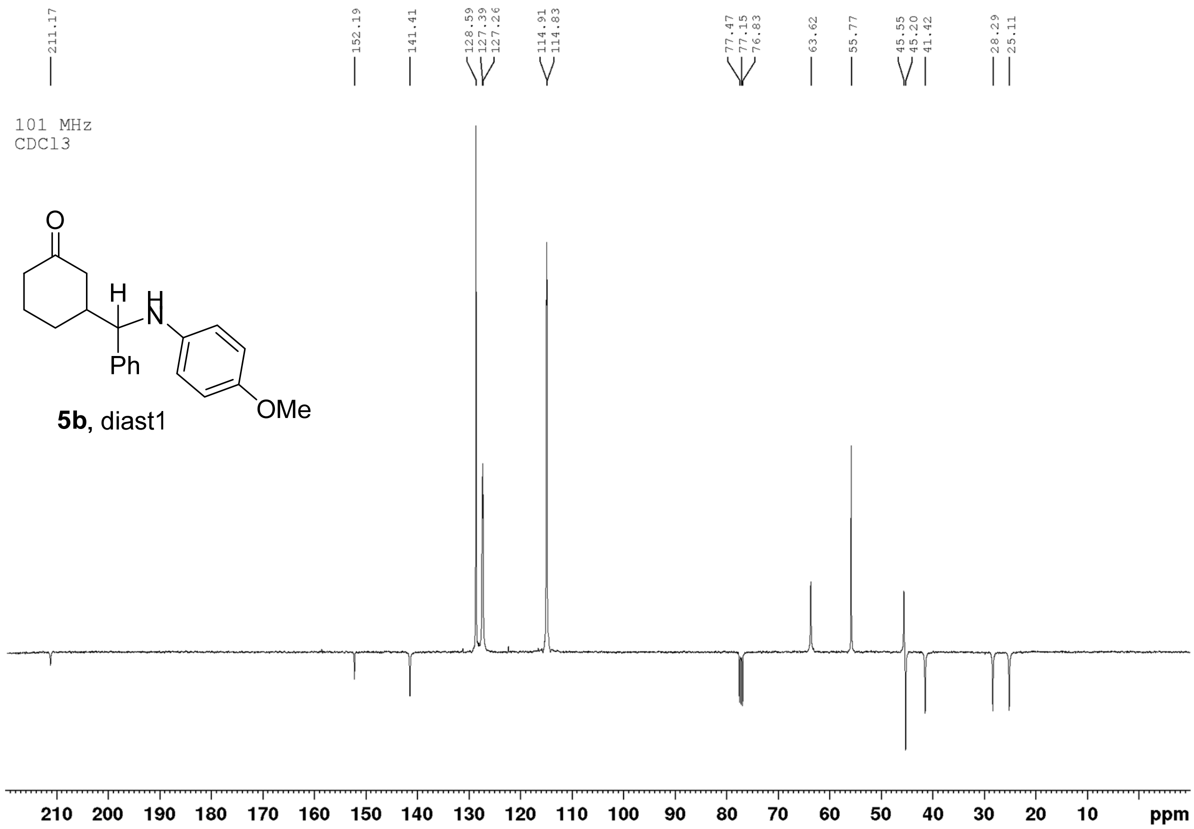
211.17
152.19
141.41
128.59
127.39
127.26
114.91
114.83
77.47
77.15
76.83
63.62
55.77
45.55
45.20
41.42
28.29
25.11
101 MHz
CDCl3
O
H
H
N
Ph
OMe
5b, diast1
210 200 190 180 170 160 150 140 130 120 110 100 90 80 70 60 50 40 30 20 10 ppm

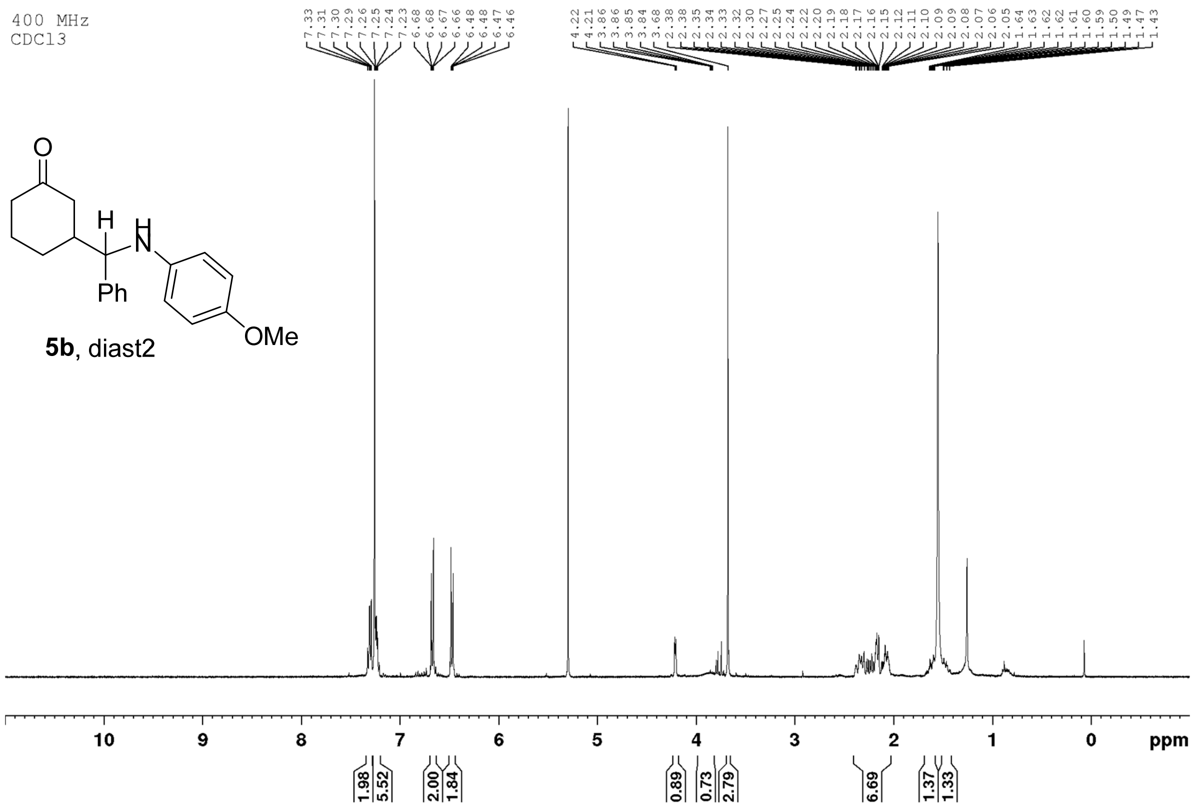
400 MHz
CDCl3
7.33 7.31 7.30 7.29 7.26 7.25 7.24 7.23 6.68 6.67 6.66 6.48 6.47 6.46
4.22 4.21 3.86 3.85 3.84 3.68 2.38 2.38 2.35 2.34 2.33 2.32 2.30 2.27 2.25 2.24 2.22 2.20 2.19 2.17 2.16 2.15 2.12 2.11 2.10 2.09 2.09 2.08 2.07 2.06 2.05 1.64 1.63 1.62 1.61 1.60 1.59 1.50 1.49 1.47 1.43
O
H
H
N
Ph
OMe
5b, diast2
10 9 8 7 6 5 4 3 2 1 0 ppm
1.98 5.52 2.00 1.84 0.89 0.73 2.79 6.69 1.37 1.33

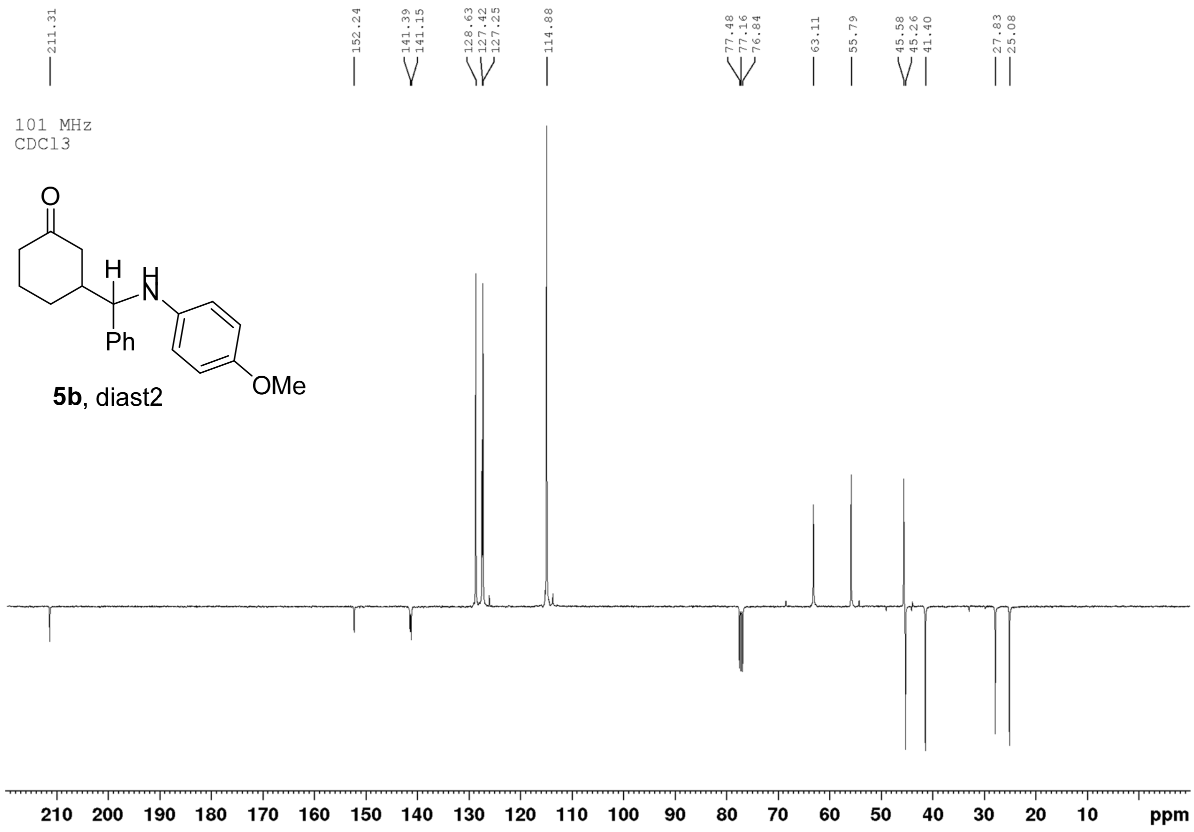
211.31
152.24
141.39
141.15
128.63
127.42
127.25
114.88
77.48
77.16
76.84
63.11
55.79
45.58
45.26
41.40
27.83
25.08
101 MHz
CDCl3
O
H
H
N
Ph
OMe
210 200 190 180 170 160 150 140 130 120 110 100 90 80 70 60 50 40 30 20 10 ppm

5b, diast2

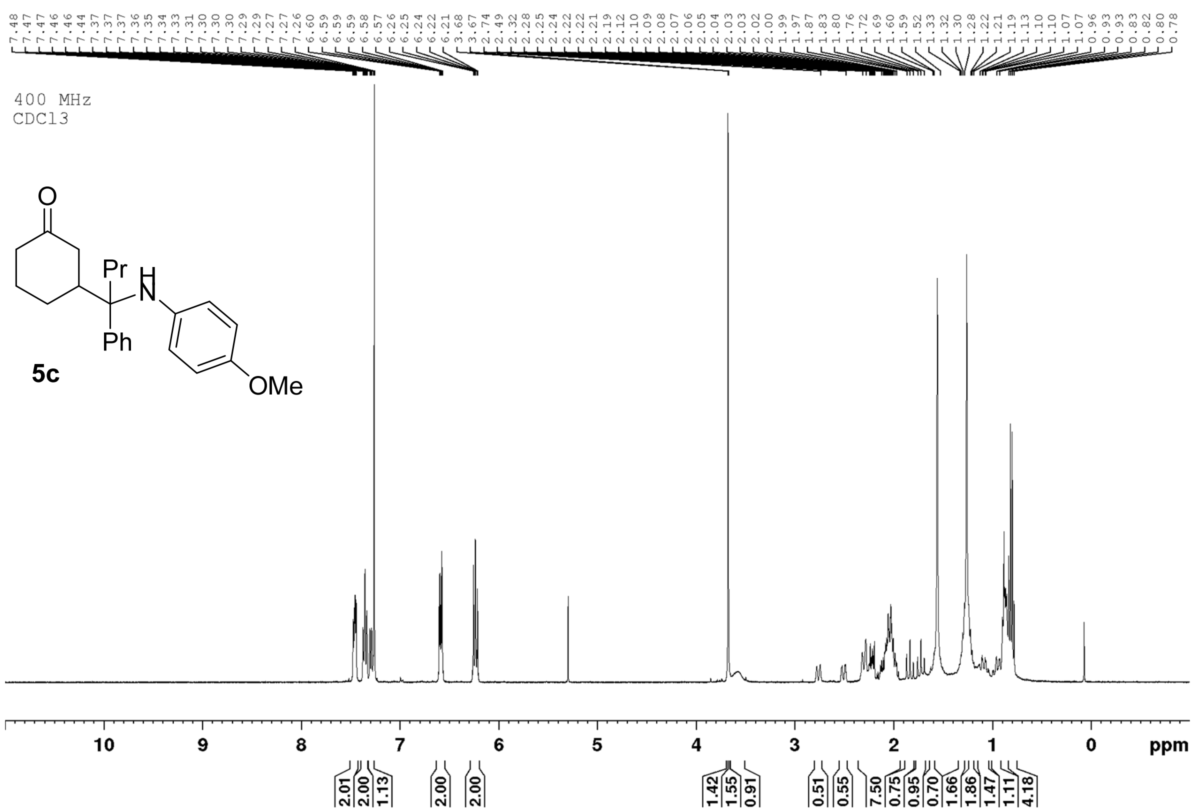
7.48 7.47 7.46 7.44 7.37 7.37 7.36 7.35 7.34 7.33 7.31 7.30 7.30 7.29 7.29 7.27 7.26 6.60 6.59 6.59 6.58 6.57 6.26 6.25 6.24 6.22 6.21 3.68 3.67 2.74 2.49 2.32 2.28 2.25 2.24 2.22 2.22 2.21 2.19 2.12 2.10 2.09 2.08 2.07 2.06 2.05 2.04 2.03 2.03 2.02 2.00 1.99 1.97 1.87 1.83 1.80 1.76 1.72 1.69 1.60 1.59 1.52 1.33 1.32 1.30 1.28 1.22 1.21 1.19 1.13 1.10 1.07 0.96 0.93 0.83 0.82 0.80 0.78
400 MHz
CDCl3
O
Pr
H
N
Ph
OMe
10 9 8 7 6 5 4 3 2 1 0 ppm
2.01 2.00 1.13 2.00 2.00 1.42 1.55 0.91 0.51 0.55 7.50 0.75 0.95 0.70 1.66 1.86 1.47 1.11 4.18

5c

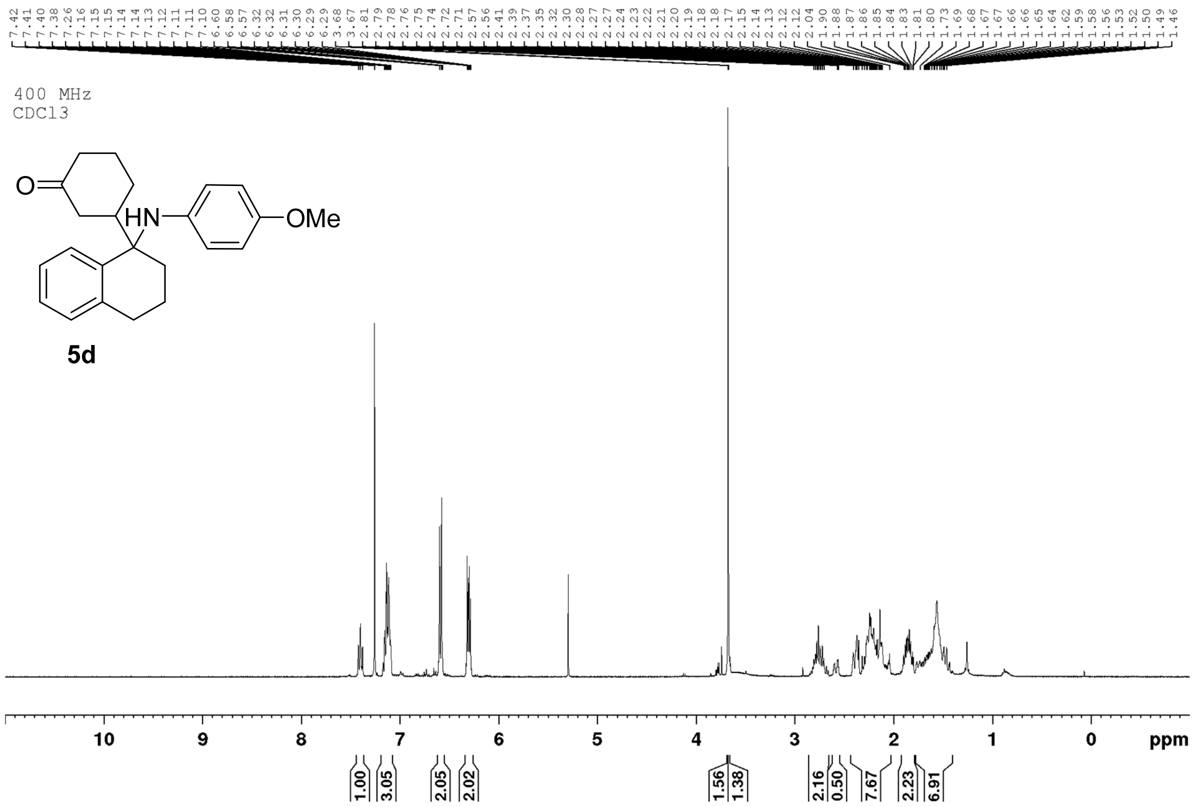
7.42 7.41 7.40 7.38 7.26 7.16 7.15 7.15 7.14 7.14 7.13 7.12 7.11 7.11 7.10 6.60 6.58 6.57 6.32 6.32 6.31 6.30 6.29 6.29 3.68 3.67 2.81 2.79 2.78 2.76 2.75 2.74 2.72 2.71 2.57 2.56 2.41 2.39 2.37 2.35 2.32 2.30 2.28 2.27 2.27 2.24 2.23 2.22 2.21 2.20 2.19 2.18 2.18 2.17 2.15 2.14 2.13 2.12 2.12 2.04 1.90 1.88 1.87 1.86 1.85 1.84 1.83 1.81 1.80 1.73 1.69 1.68 1.67 1.67 1.66 1.66 1.65 1.64 1.62 1.59 1.58 1.56 1.53 1.52 1.50 1.49 1.46
400 MHz
CDCl3
O
HN
OMe
5d
10 9 8 7 6 5 4 3 2 1 0 ppm
1.00 3.05 2.05 2.02 1.56 1.38 2.16 0.50 7.67 2.23 6.91

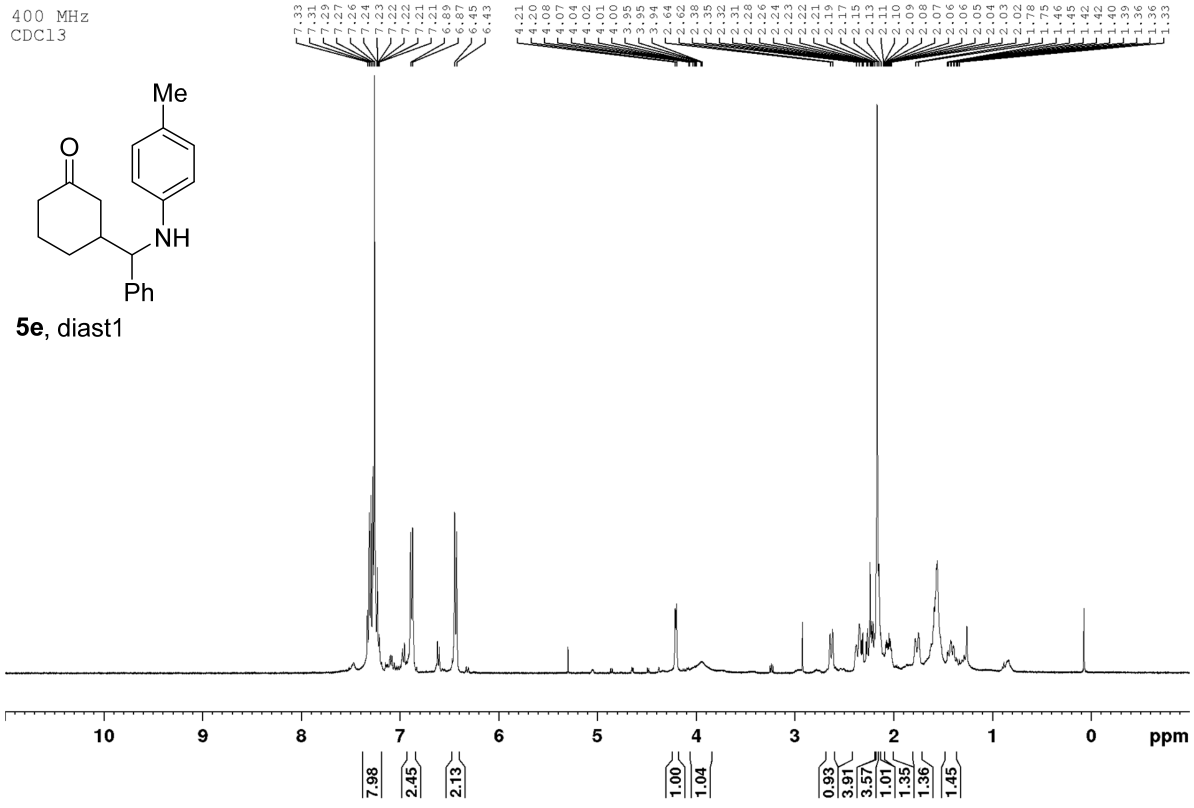
400 MHz
CDCl3
7.33 7.31 7.29 7.27 7.26 7.24 7.23 7.22 7.22 7.21 7.21 6.89 6.87 6.45 6.43
4.21 4.20 4.08 4.07 4.04 4.02 4.01 4.00 3.95 3.94 2.64 2.62 2.38 2.35 2.32 2.31 2.28 2.26 2.24 2.23 2.22 2.21 2.19 2.17 2.15 2.13 2.11 2.10 2.09 2.08 2.07 2.06 2.06 2.05 2.04 2.03 2.02 1.78 1.75 1.46 1.45 1.42 1.40 1.39 1.36 1.36 1.33
Me
O
NH
Ph
5e, diast1
10 9 8 7 6 5 4 3 2 1 0 ppm
7.98 2.45 2.13 1.00 1.04 0.93 3.91 3.57 1.01 1.35 1.36 1.45

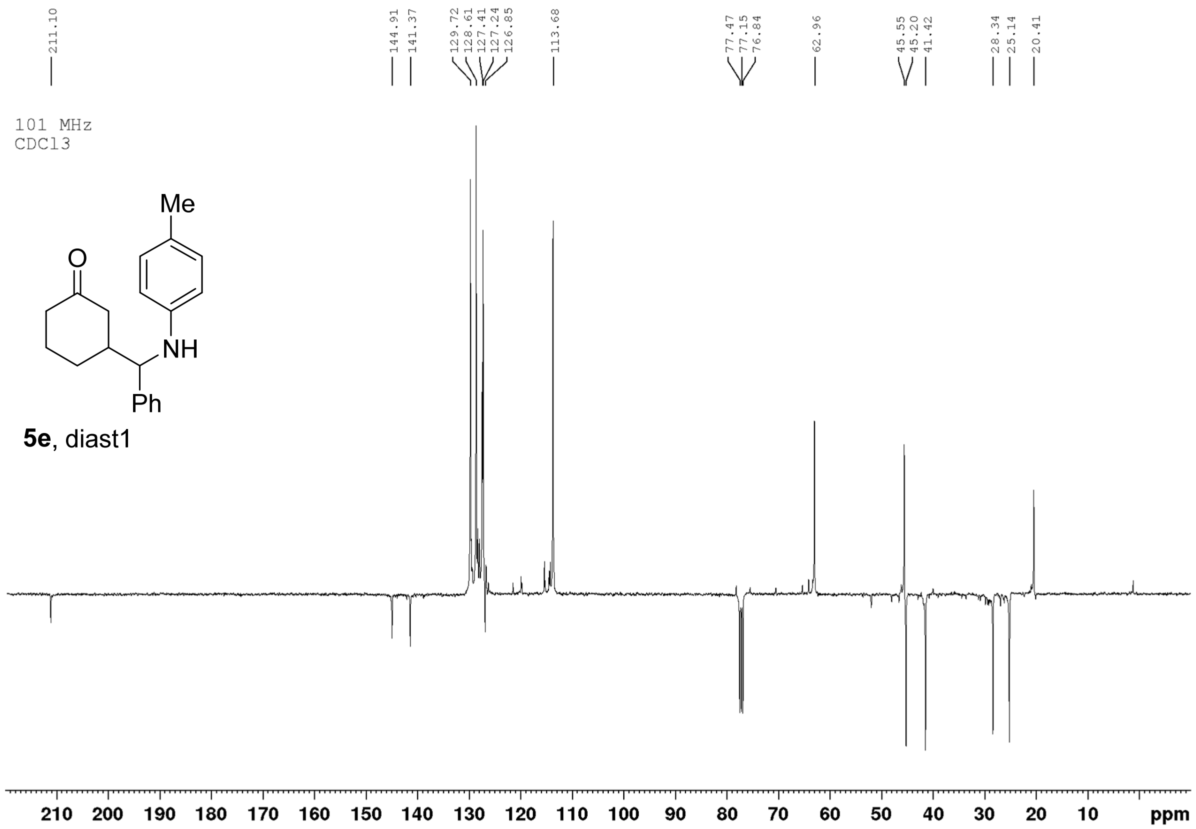

5e, diast1

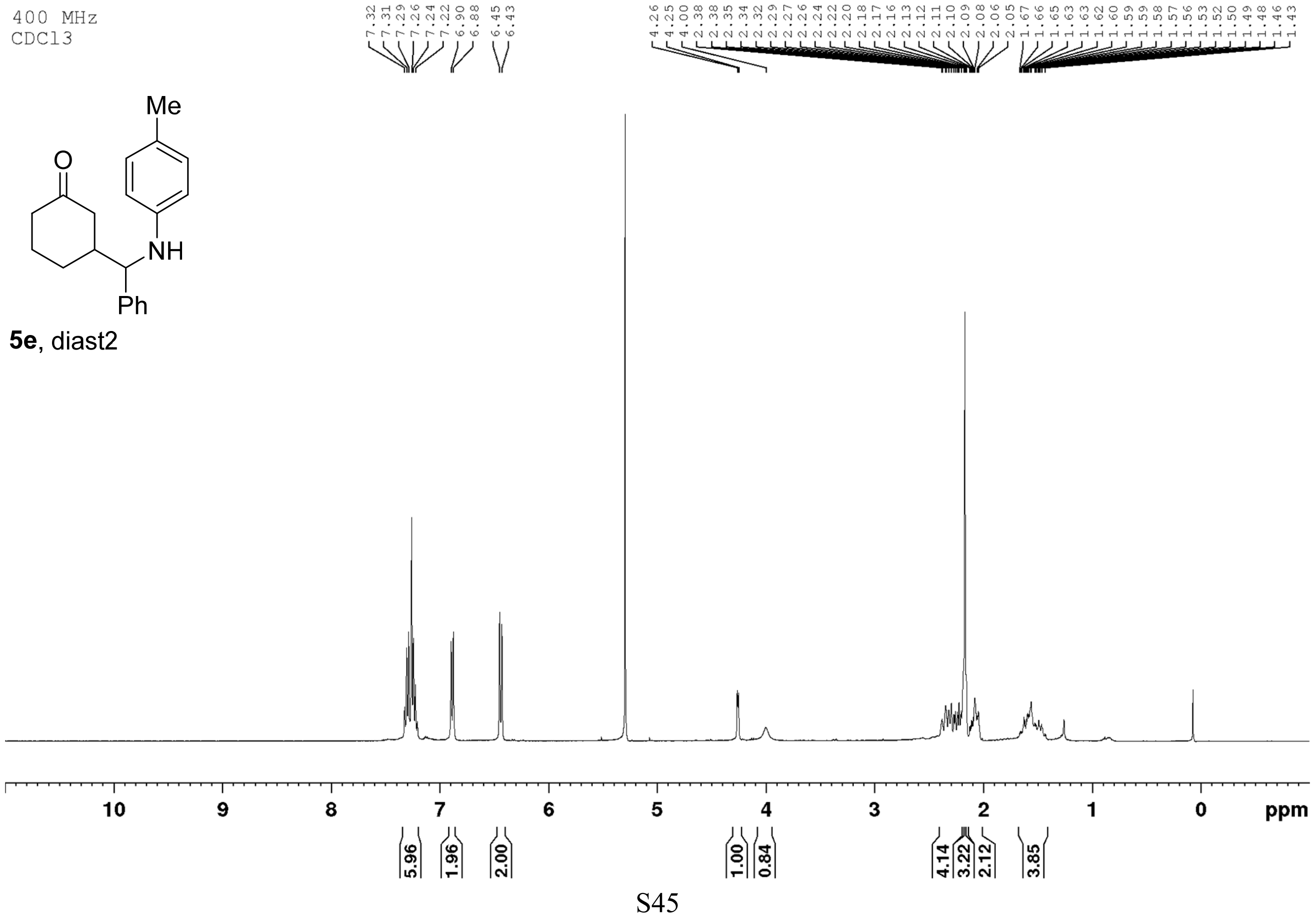

5e, diast2

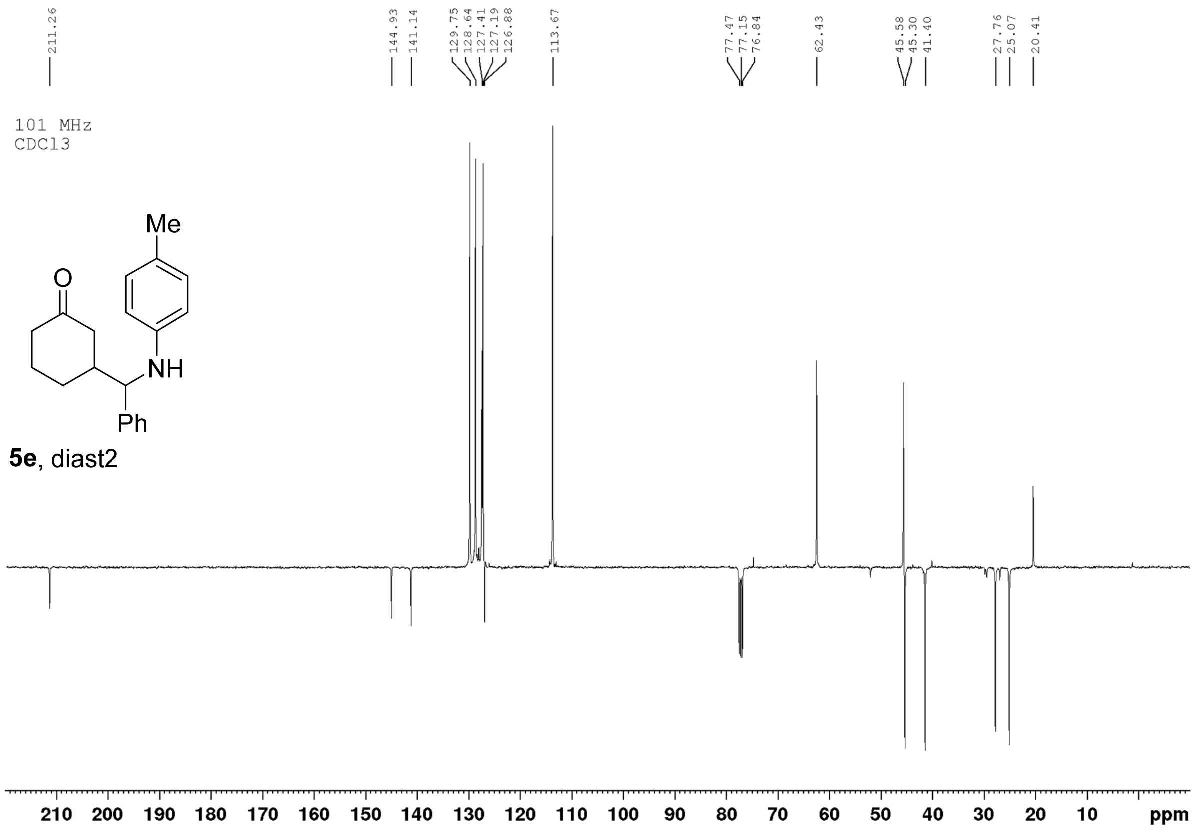
101 MHz
CDCl3
Me
O
NH
Ph
5e, diast2
ppm

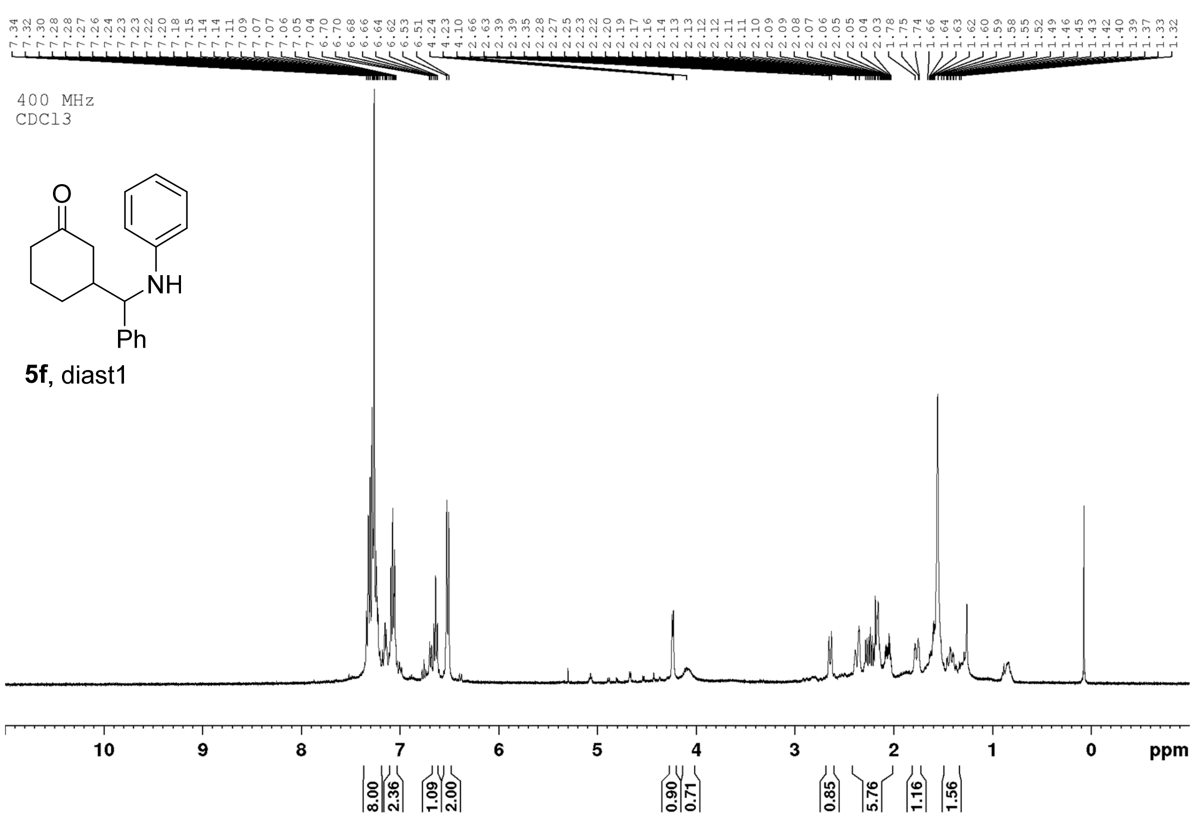
400 MHz
CDCl3
O
NH
Ph
5f, diast1
ppm

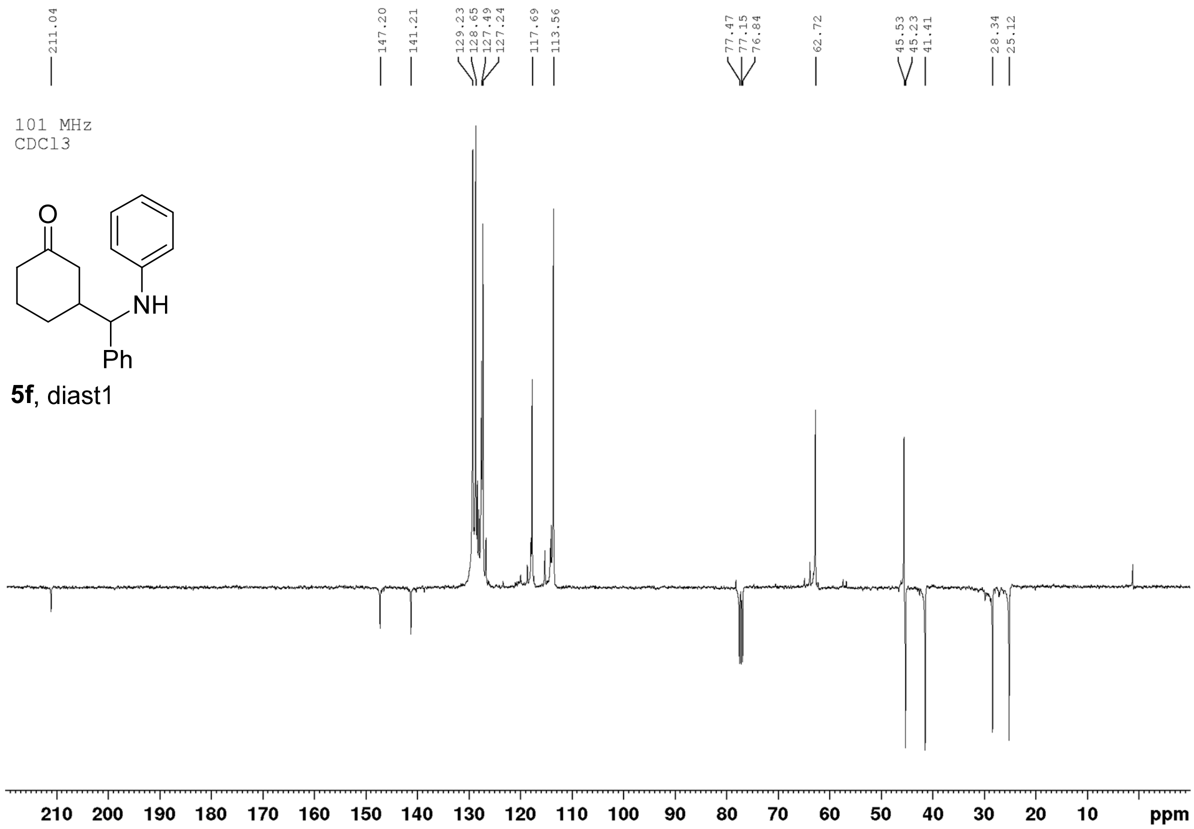
211.04
147.20
141.21
129.23
128.65
127.49
127.24
117.69
113.56
77.47
77.15
76.84
62.72
45.53
45.23
41.41
28.34
25.12
101 MHz
CDCl3
O
NH
Ph
5f, diast1
210 200 190 180 170 160 150 140 130 120 110 100 90 80 70 60 50 40 30 20 10 ppm

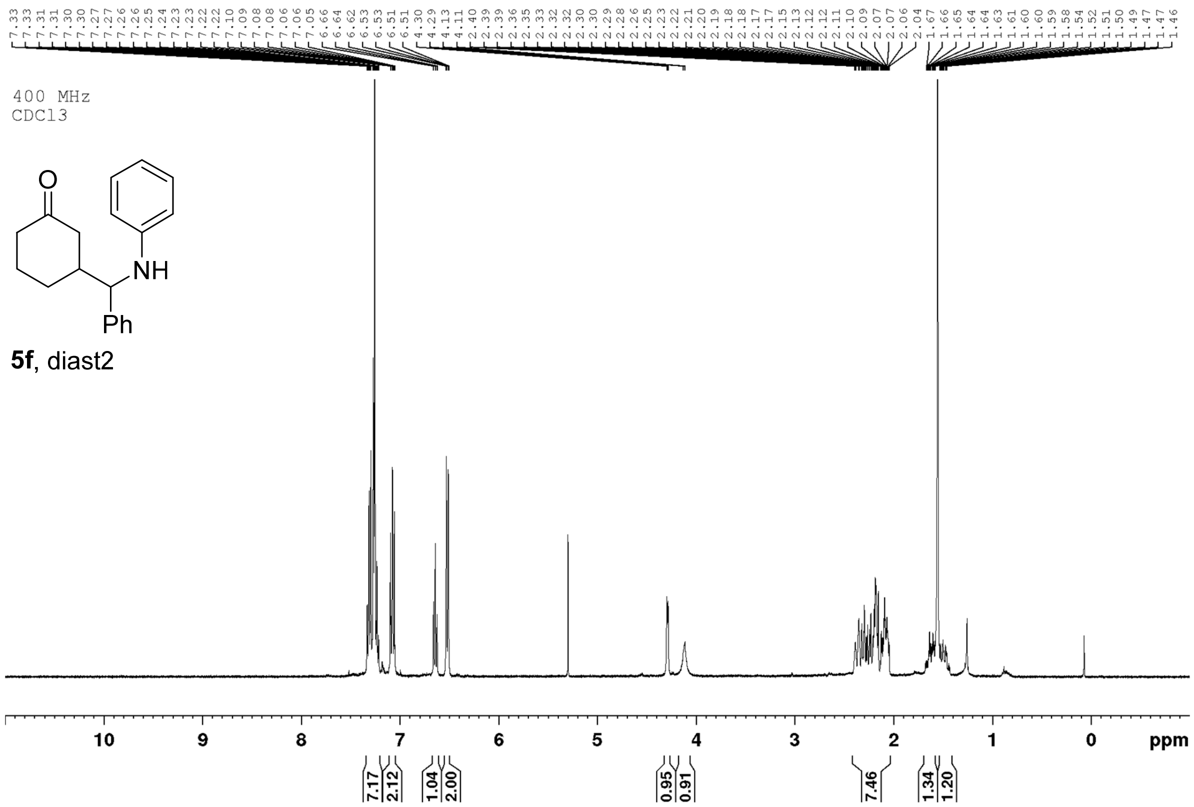
7.33 7.31 7.31 7.30 7.27 7.26 7.25 7.24 7.23 7.22 7.22 7.10 7.09 7.08 7.06 7.05 6.66 6.64 6.62 6.53 6.53 6.51 4.30 4.29 4.13 4.11 2.40 2.39 2.36 2.35 2.33 2.32 2.32 2.30 2.30 2.29 2.28 2.26 2.25 2.23 2.22 2.21 2.20 2.19 2.18 2.18 2.17 2.17 2.15 2.13 2.12 2.12 2.11 2.10 2.09 2.07 2.06 2.04 1.67 1.66 1.65 1.64 1.63 1.61 1.60 1.59 1.58 1.54 1.52 1.51 1.50 1.49 1.47 1.46
400 MHz
CDCl3
O
NH
Ph
5f, diast2
10 9 8 7 6 5 4 3 2 1 0 ppm
7.17 2.12 1.04 2.00 0.95 0.91 7.46 1.34 1.20

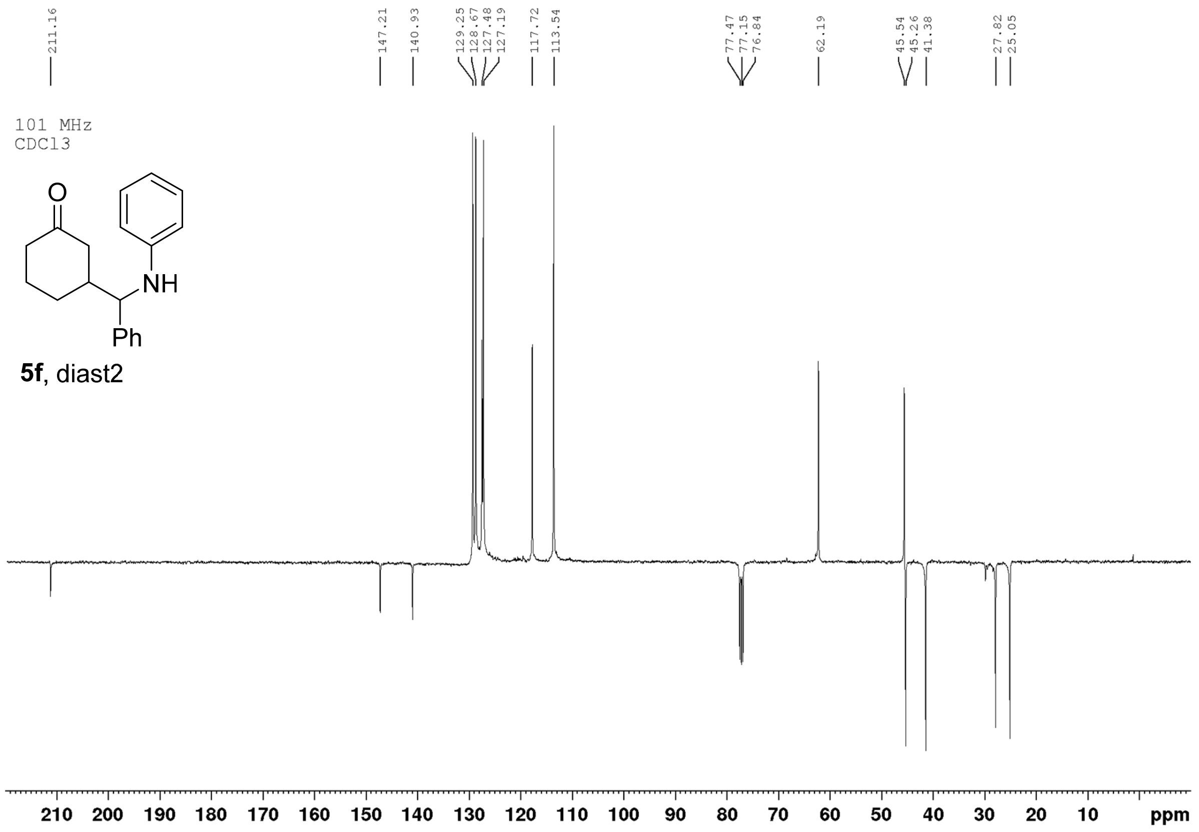
211.16
147.21
140.93
129.25
128.67
127.48
127.19
117.72
113.54
77.47
77.15
76.84
62.19
45.54
45.26
41.38
27.82
25.05
101 MHz
CDCl3
O
NH
Ph
5f, diast2
210 200 190 180 170 160 150 140 130 120 110 100 90 80 70 60 50 40 30 20 10 ppm

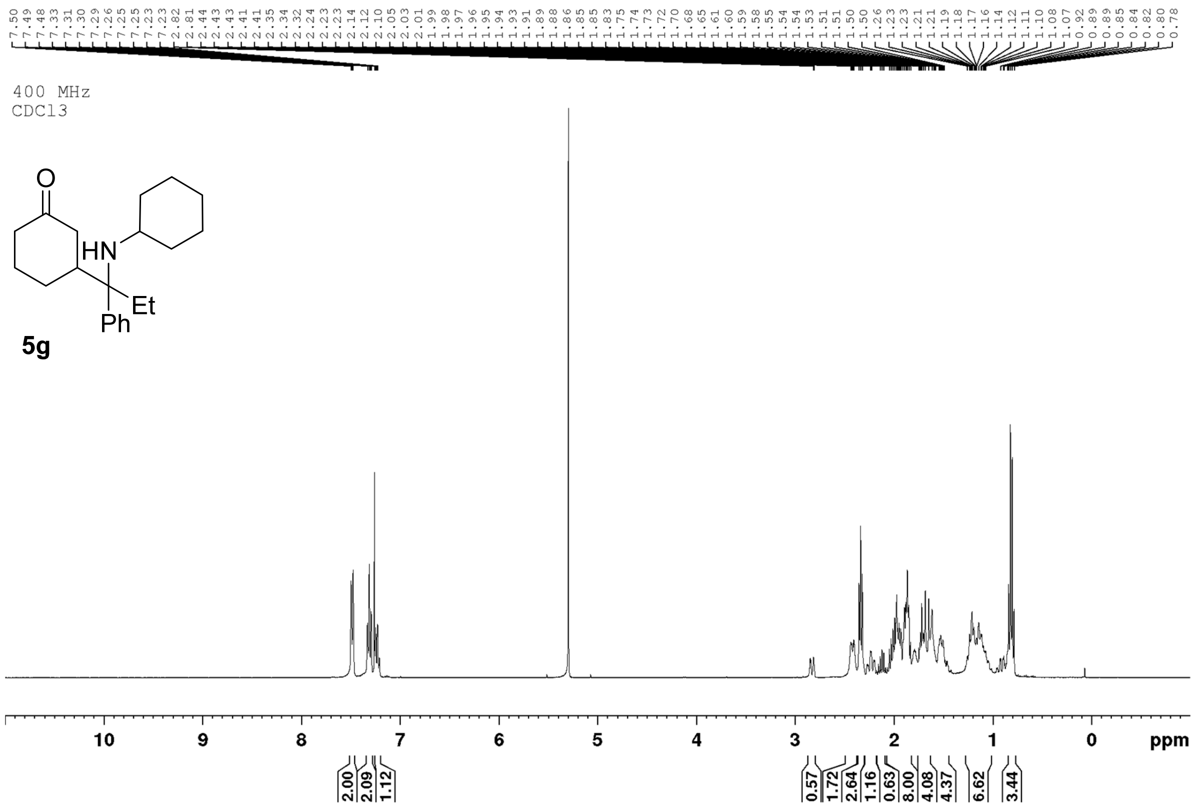
400 MHz
CDCl3
O
HN
Et
Ph
5g
10 9 8 7 6 5 4 3 2 1 0 ppm
2.00
2.09
1.12
0.57
1.72
2.64
1.16
0.63
8.00
4.08
4.37
6.62
3.44

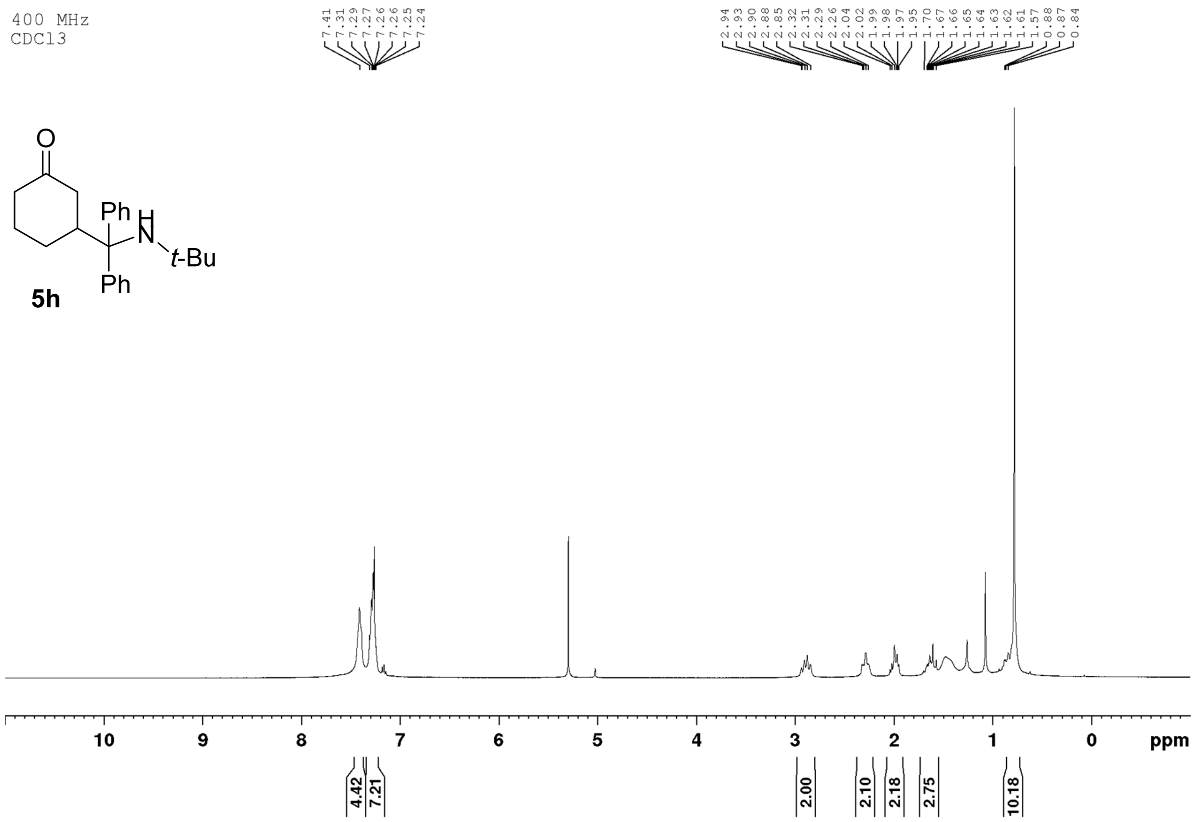
400 MHz
CDCl3
7.41 7.31 7.29 7.27 7.26 7.26 7.25 7.24
2.94 2.93 2.90 2.88 2.85 2.32 2.31 2.29 2.26 2.04 2.02 1.99 1.98 1.97 1.95 1.70 1.67 1.66 1.65 1.64 1.63 1.62 1.61 1.57 0.88 0.87 0.84
O
Ph
H
N
t-Bu
Ph
5h
10 9 8 7 6 5 4 3 2 1 0 ppm
4.42 7.21 2.00 2.10 2.18 2.75 10.18

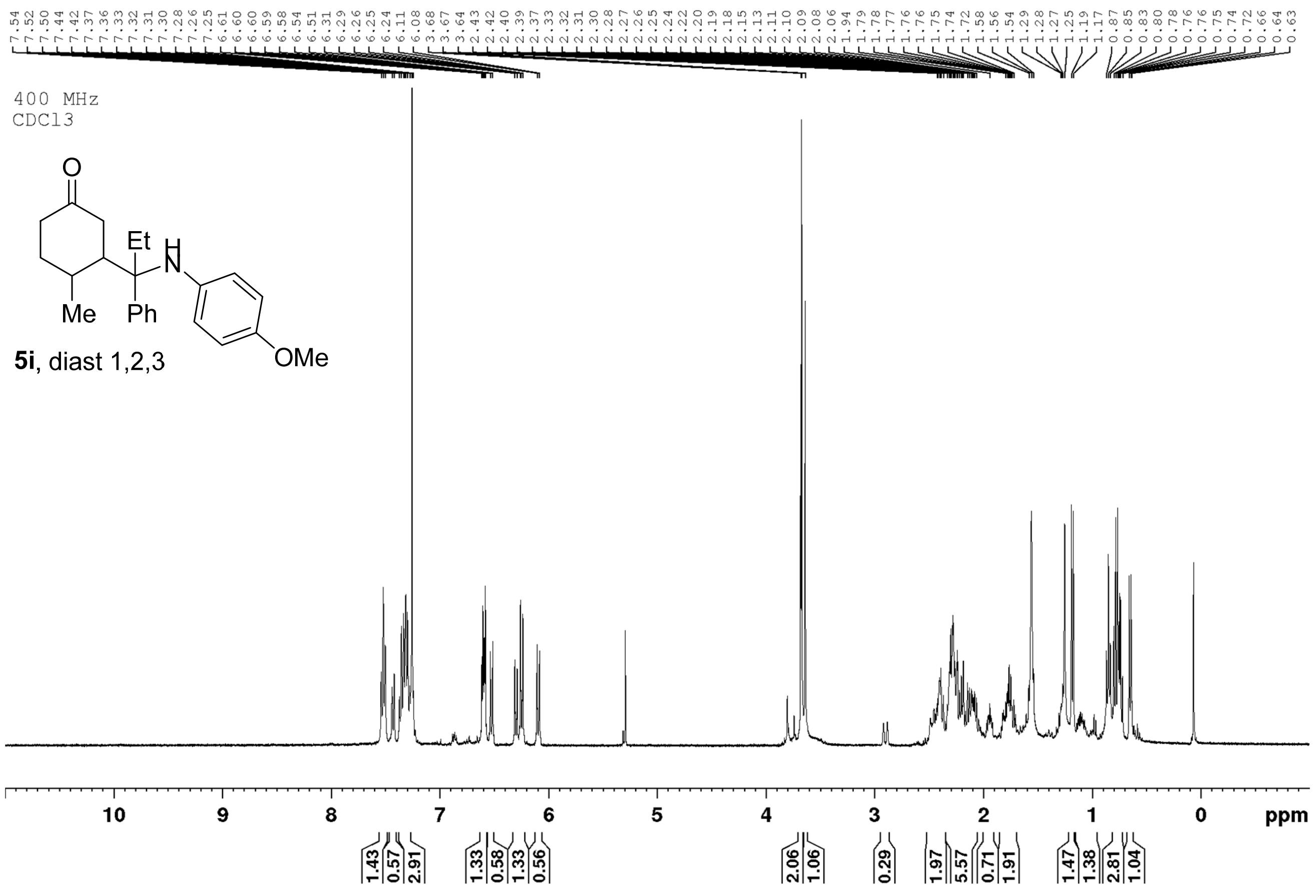
7.54 7.52 7.50 7.44 7.42 7.37 7.36 7.33 7.32 7.31 7.30 7.28 7.26 7.25 6.61 6.60 6.59 6.58 6.54 6.51 6.31 6.29 6.26 6.25 6.24 6.11 6.08 3.68 3.67 3.64 2.43 2.42 2.40 2.39 2.37 2.33 2.32 2.31 2.30 2.28 2.27 2.26 2.25 2.24 2.22 2.20 2.19 2.18 2.15 2.13 2.11 2.10 2.09 2.08 2.06 1.94 1.79 1.78 1.77 1.76 1.75 1.74 1.72 1.58 1.56 1.54 1.29 1.28 1.27 1.25 1.19 1.17 0.87 0.85 0.83 0.80 0.78 0.76 0.76 0.75 0.74 0.72 0.66 0.64 0.63
400 MHz
CDCl3
O
Et
H
N
Me
Ph
OMe
5i, diast 1,2,3
10 9 8 7 6 5 4 3 2 1 0 ppm
1.43 0.57 2.91 1.33 0.58 1.33 0.56 2.06 1.06 0.29 1.97 5.57 0.71 1.91 1.47 1.38 2.81 1.04

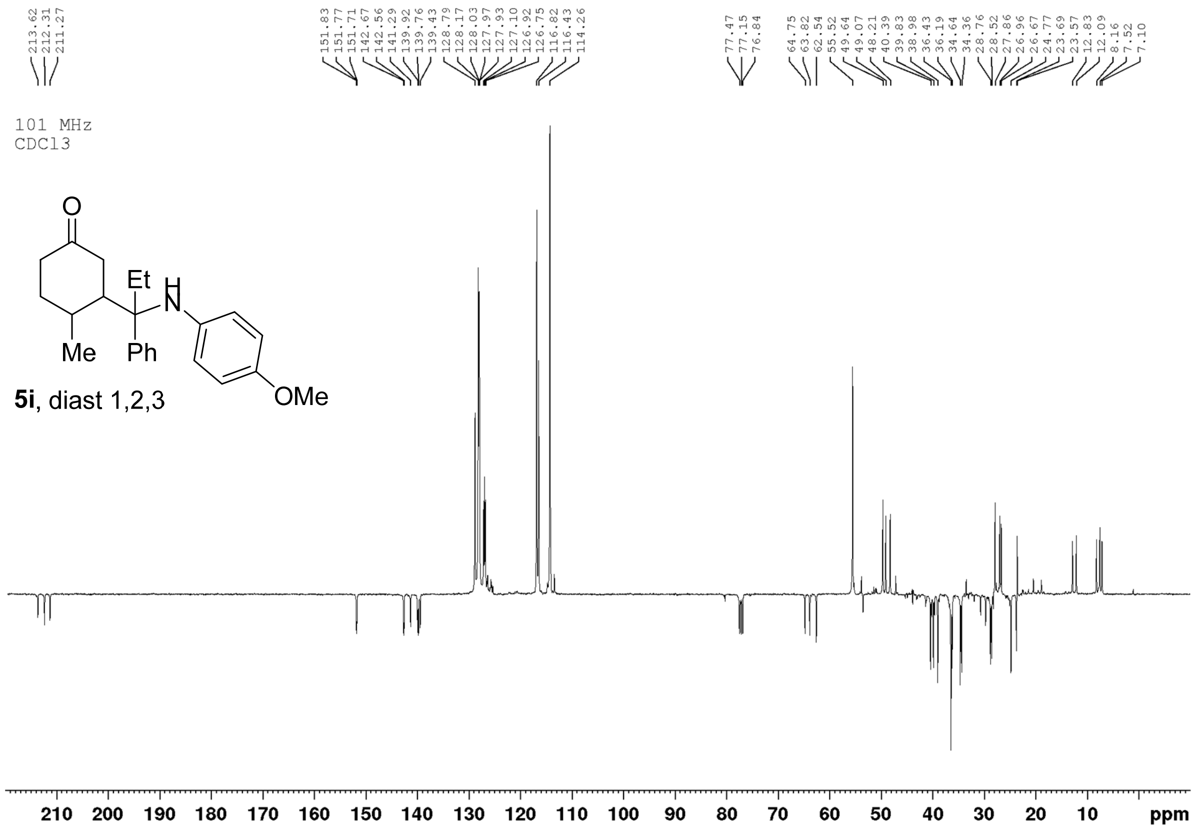
213.62
212.31
211.27
151.83
151.77
151.71
142.67
142.56
141.29
139.92
139.76
139.43
128.79
128.17
128.03
127.97
127.93
127.10
126.92
126.75
116.82
116.43
114.26
77.47
77.15
76.84
64.75
63.82
62.54
55.52
49.64
49.07
48.21
40.39
39.83
38.98
36.43
36.19
34.64
34.36
28.76
28.52
27.86
26.96
26.67
24.77
23.69
23.57
12.83
12.09
8.16
7.52
7.10
101 MHz
CDCl3
O
Et
H
N
Me
Ph
OMe
5i, diast 1,2,3
210 200 190 180 170 160 150 140 130 120 110 100 90 80 70 60 50 40 30 20 10 ppm

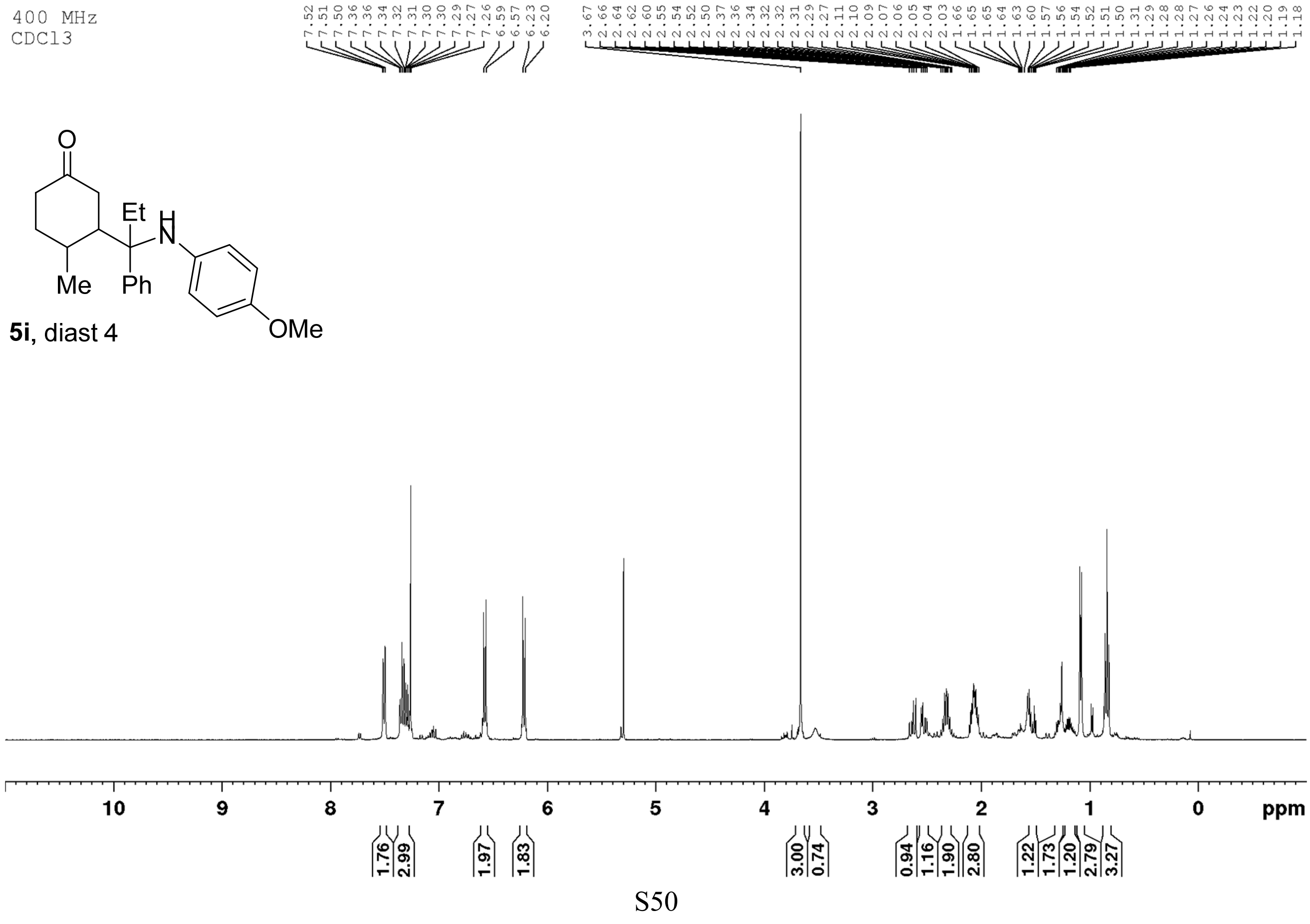
400 MHz
CDCl3
7.52
7.51
7.50
7.36
7.34
7.32
7.31
7.30
7.29
7.27
7.26
6.59
6.57
6.23
6.20
3.67
2.66
2.64
2.62
2.60
2.55
2.54
2.52
2.50
2.37
2.36
2.34
2.32
2.31
2.29
2.27
2.11
2.10
2.09
2.07
2.06
2.05
2.04
2.03
1.66
1.65
1.64
1.63
1.60
1.57
1.56
1.54
1.52
1.51
1.50
1.31
1.29
1.28
1.28
1.27
1.26
1.24
1.23
1.22
1.20
1.19
1.18
O
Et
H
N
Me
Ph
OMe
5i, diast 4
10 9 8 7 6 5 4 3 2 1 0 ppm
1.76
2.99
1.97
1.83
3.00
0.74
0.94
1.16
1.90
2.80
1.22
1.73
1.20
2.79
3.27

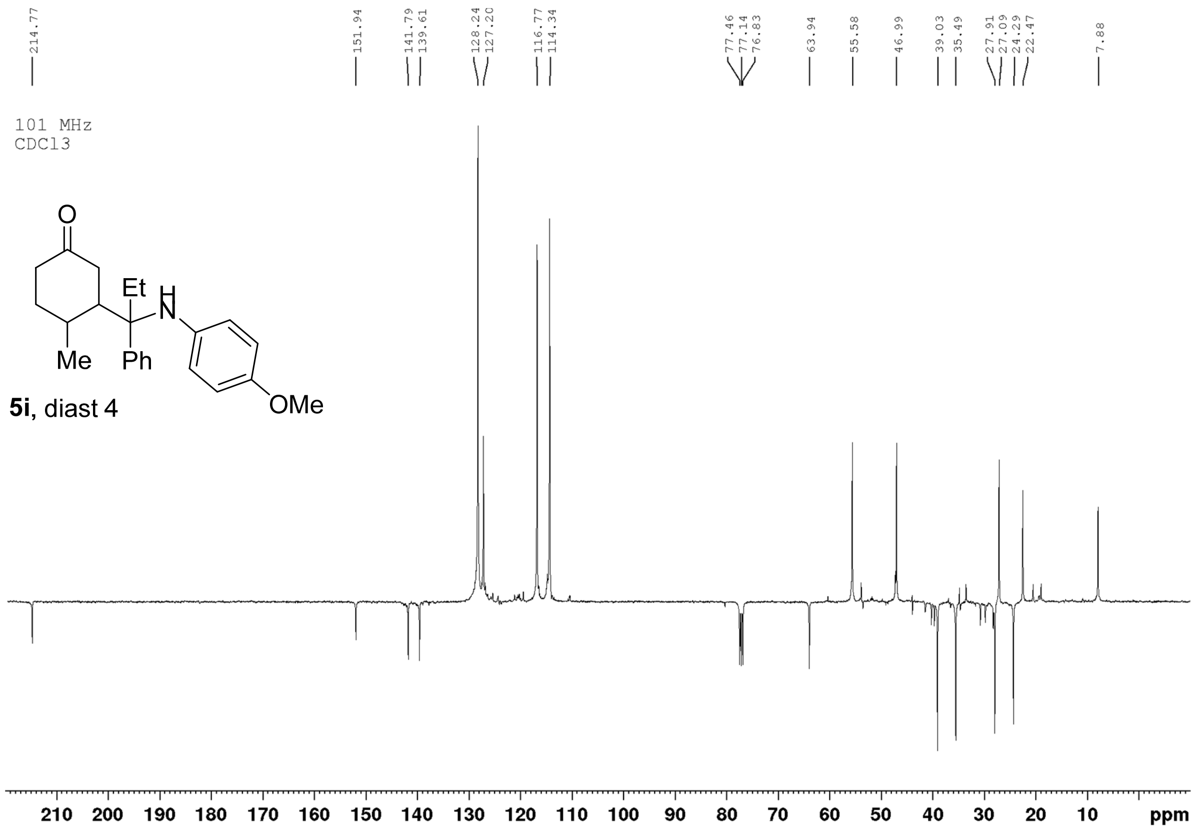

214.77
151.94
141.79
139.61
128.24
127.20
116.77
114.34
77.46
77.14
76.83
63.94
55.58
46.99
39.03
35.49
27.91
27.09
24.29
22.47
7.88
101 MHz
CDCl3
O
Et
H
N
Me
Ph
OMe
5i, diast 4
210 200 190 180 170 160 150 140 130 120 110 100 90 80 70 60 50 40 30 20 10 ppm

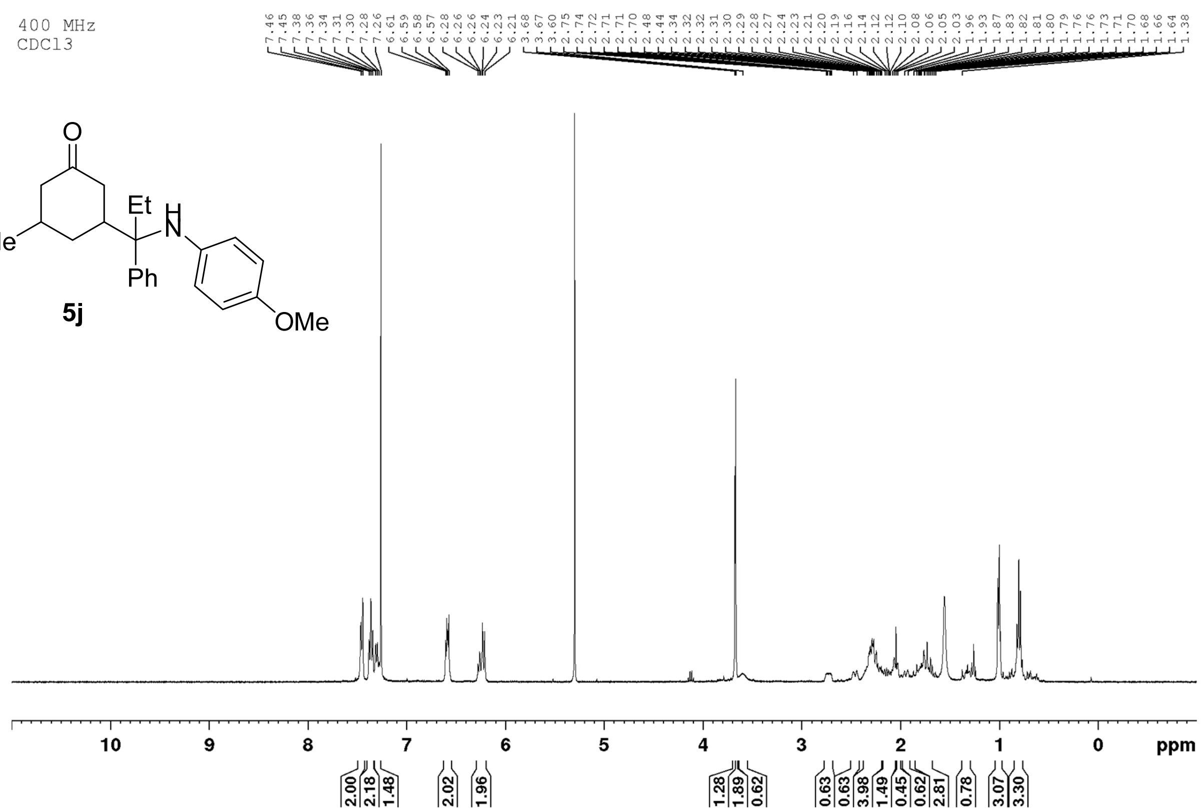

400 MHz
CDCl3
7.46 7.45 7.38 7.36 7.34 7.31 7.30 7.28 7.26 6.61 6.59 6.58 6.57 6.28 6.26 6.24 6.23 6.21 3.68 3.67 3.60 2.75 2.74 2.72 2.71 2.70 2.48 2.44 2.34 2.32 2.31 2.30 2.29 2.28 2.27 2.24 2.23 2.21 2.20 2.19 2.16 2.14 2.12 2.10 2.08 2.06 2.05 2.03 1.96 1.93 1.87 1.83 1.82 1.81 1.80 1.79 1.76 1.73 1.71 1.70 1.68 1.66 1.64 1.38
O
Et
H
N
Me
Ph
OMe
5j
10 9 8 7 6 5 4 3 2 1 0 ppm
2.00 2.18 1.48 2.02 1.96 1.28 1.89 0.62 0.63 0.63 3.98 1.49 0.45 0.62 2.81 0.78 3.07 3.30

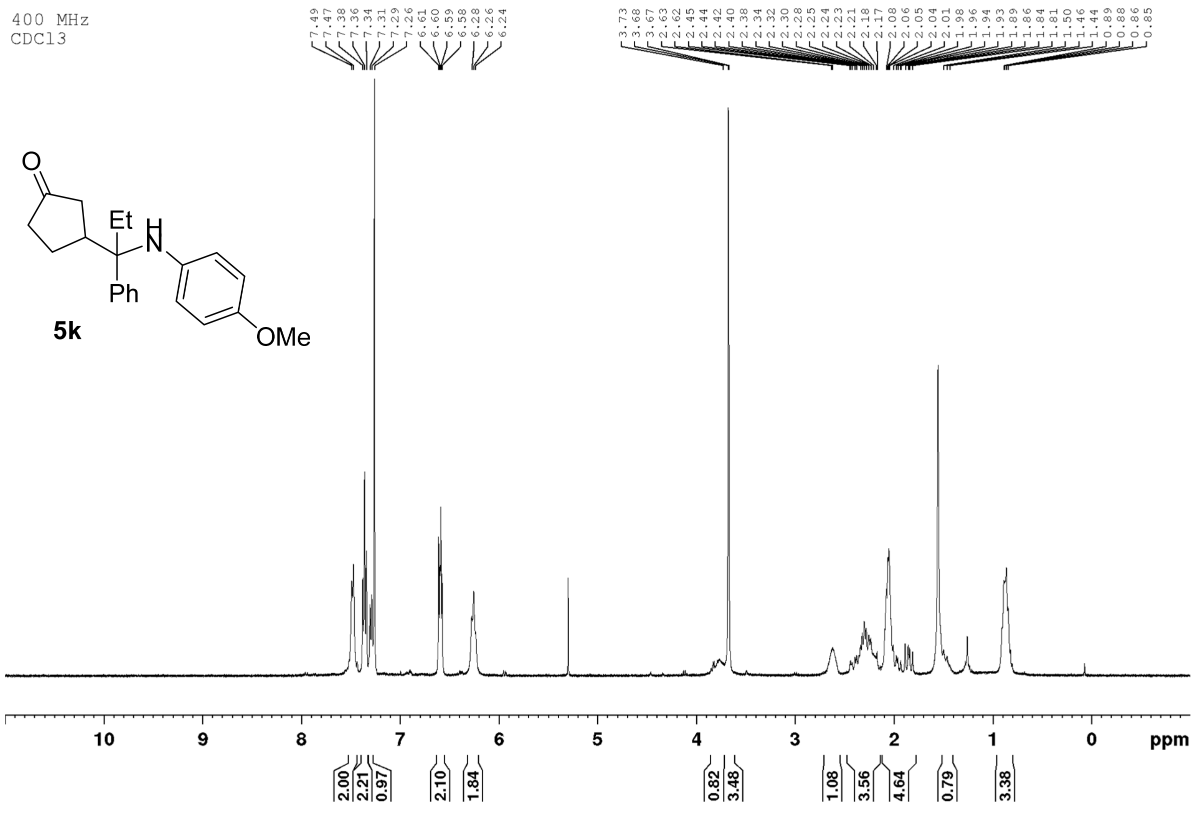
400 MHz
CDCl3
7.49 7.47 7.38 7.36 7.34 7.31 7.29 7.26 6.61 6.60 6.59 6.58 6.28 6.26 6.24
3.73 3.68 3.67 2.63 2.62 2.45 2.44 2.42 2.40 2.38 2.34 2.32 2.30 2.28 2.25 2.24 2.23 2.21 2.18 2.17 2.08 2.06 2.05 2.04 2.01 1.98 1.96 1.94 1.93 1.89 1.86 1.84 1.81 1.50 1.46 1.44 0.89 0.88 0.86 0.85
O
Et
H
N
Ph
OMe
5k
10 9 8 7 6 5 4 3 2 1 0 ppm
2.00 2.21 0.97 2.10 1.84 0.82 3.48 1.08 3.56 4.64 0.79 3.38

References

[1] Boris, A. M. *Organometallics* **2023**, *42*, 182.
[2] Still, W.C.; Kahn, M.; Mitra, A. *J. Org. Chem.* **1978**, *43*, 2923.
[3] Fulmer, G. R.; Miller , A. J. M.; Sherden, N. H.; Gottlieb, H. E.; Nudelman, A.; Stoltz, B. M.; Bercaw, J. E.; Goldberg, K. I. *Organometallics* **2010**, *29*, 2176.
[4] Luo, J.; Zhang, J. *ACS Catal.* **2016**, *6*, 873.
[5] Rasu, L.; Amiri, M.; Bergens, S. H. *ACS Appl. Mater. Interfaces* **2021**, *13*, 17745.
[6] Speckmeier, E.; Fischer, T. G.; Zeitler, K. *J. Am. Chem. Soc.* **2018**, *140*, 15353.
[7] Chernowsky, C. P.; Chmiel, A. F.; Wickens, Z. K. *Angew. Chem. Int. Ed.* **2021**, *60*, 21418
[8] Pezzetta, C.; Folli, A.; Matuszewska, O.; Murphy, D.; Davidson, R. W. M.; Bonifazi, D. *Adv. Synth. Catal.* **2021**, *363*, 4740.
[9] Terrett, J. A.; Clift, M. D.; MacMillan, D. W. C. *J. Am. Chem. Soc.* **2014**, *136*, 6858.
[10] Buxton, C. S.; Blakemore, D. C.; Bower, J. F. *Angew. Chem. Int. Ed.* **2017**, *56*, 13824.
[11] Grünenfelder, C. E.; Kisunzu, J. K.; Wennemers, H. *Angew. Chem. Int. Ed.* **2016**, *55*, 8571.
[12] Known compound (Di Sabato, A.; D'Acunzo, F.; Filippini, D.; Vetica, F.; Brasiello, A.; Corinti, D.; Bodo, E.; Michenzi, C.; Panzetta, E.; Gentili, P. *J. Org. Chem.* **2022**, *87*, 13803) prepared following the procedure described in Ref. 11.
[13] Known compound (Tsukamoto, Y.; Itoh, S.; Kobayashi, M.; Obora, Y. *Org. Lett.* **2019**, *21*, 3299) prepared following the procedure described in Ref. 10.
[14] Known compound (Zhu, Q.; Nocera, D. G. *J. Am. Chem. Soc.* **2020**, *142*, 17913) prepared according to the procedures described in: Stivanin, M. L.; Fernandes, A. A. G.; da Silva, A. F.; Okada Jr, C.Y; Jurberg, I. D. *Adv. Synth. Catal.* **2020**, *362*, 1106 (methylation of arylacetic acids); Jiang, B.; Zhao, M.; Li, S.; Xu, Y.-H.; Loh, T.-P. *Angew. Chem. Int. Ed.* **2018**, *57*, 555 (acrylate synthesis).
[15] Known compound (Hu, T.-J.; Zhang, G.; Chen, Y.-H.; Feng, C.-G.; Lin, G.-Q. *J. Am. Chem. Soc.* **2016**, *138*, 2897) prepared following the procedures described in Ref. 14.
[16] Petronijević, F. R.; Nappi, M.; MacMillan, D. W. C. *J. Am. Chem. Soc.* **2013**, *135*, 18323.
[17] Known compound (Wiedenhoeft, D.; Benoit, A. R.; Wu, Y., Porter, J. D.; Meyle, E.; Yeung, T. H. W.; Huff, R.; Lindeman, S. V.; Dockendorff, C. *Tetrahedron* **2016**, *72*, 3905) prepared following the procedure described in Ref. 14.
[18] Jeffrey, J. L.; Petronijević, F. R.; MacMillan, D. W. C. *J. Am. Chem. Soc.* **2015**, *137*, 8404.
[19] Vailati Facchini, S.; Cettolin, M.; Bai, X.; Casamassima, G.; Pignataro, L.; Gennari, C.; Piarulli, U. *Adv. Synth. Catal.* **2018**, *360*, 1054.
[20] Known compound (Leitch, J. A.; Rogova, T.; Duarte, F.; Dixon, D. J. *Angew. Chem. Int. Ed.* **2020**, *59*, 4121) prepared following the procedure described in Ref. 19.
[21] Known compound (Saito, K.; Horiguchi, K.; Shibata, Y.; Yamanaka, M.; Akiyama, T. *Chem. Eur. J.* **2014**, *20*, 7616) prepared following the procedure described in Ref. 19.
[22] Known compound (Monopoli, A.; Cotugno, P.; Iannone, F.; Ciminale, F.; Dell'Anna, M. M.; Mastrorilli, P.; Nacci; A. *Eur. J. Org. Chem.* **2014**, 5925) prepared following the procedure described in Ref. 19.
[23] Known compound (Mercea, D. M.; Howlett, M. G.; Piascik, A. D.; Scott, D. J.; Steven, A.; Ashley, A. E.; Fuchter, M. J. *Chem. Comm.* **2019**, *55*, 7077) prepared following the procedure described in Ref. 19.
[24] Known compound (Barzanò, G.; Mao, R.; Garreau, M.; Waser, J.; Hu, X. *Org. Lett.* **2020**, *22*, 5412) prepared following the procedure described in: Dai, W.; Srinivasan, R.; Katzenellenbogen, J. A. *J. Org. Chem.* **1989**, *54*, 2204.
